# Validation of an individualized reduction of falls intervention program among wheelchair and scooter users with multiple sclerosis

**DOI:** 10.1097/MD.0000000000015418

**Published:** 2019-05-13

**Authors:** Laura A. Rice, Elizabeth W. Peterson, Deborah Backus, JongHun Sung, Rebecca Yarnot, Libak Abou, Toni Van Denend, Sa Shen, Jacob J. Sosnoff

**Affiliations:** a Department of Kinesiology and Community Health, College of Applied Health Sciences, University of Illinois at Urbana-Champaign; b Department of Occupational Therapy, College of Applied Health Sciences, University of Illinois at Chicago, Chicago; c Crawford Research Institute, Shepherd Center, Atlanta, Georgia; d Center for Health, Aging and Disability, College of Applied Health Sciences, University of Illinois at Urbana-Champaign, Illinois.

**Keywords:** accidental falls, education, wheelchair

## Abstract

**Introduction:**

Falls are a serious concern for wheelchair and scooter users with multiple sclerosis (MS). Approximately, 75% of the population reports at least one fall in a 6-month period and nearly half report frequent falls. Falls can result in physical injuries and contribute to activity curtailment. Despite the negative consequences, limited evidenced-based fall prevention programs designed specifically for wheelchair and scooter users with MS exist.

**Purpose:**

Recognizing the threat falls pose to health and well-being and the dearth of fall prevention programs, the purpose of this study is to perform a structured process evaluation and examine the feasibility and efficacy of a community-based intervention specifically designed to reduce fall incidence among wheelchair and scooter users with MS. Secondary aims of the intervention are to improve functional mobility skills associated with fall risk (e.g., transfer and wheelchair skills, balance), increase knowledge of fall risk factors, decrease fear of falling, and enhance quality of life and community participation.

**Methods:**

To evaluate our specific aims, a clinical trial will be performed with 160 wheelchair and scooter users with MS. (ClinicalTrials.gov Identifier: NCT03705364). Participants will be recruited to participate in a small group-style community-based program. The content of the program will be based on factors found to be associated with falls among wheelchair and scooter users with MS. These factors include but are not limited to, wheelchair/scooter related characteristics, transfer activities, impaired seated balance, and environmental factors. A physical or occupational therapist, will implement the intervention, which is comprised of 6 sessions that occur once weekly. The incidence of falls, along with an examination of wheelchair/scooter and transfer skills, seated postural control and knowledge of fall related risk factors will be compared between intervention and control participants, with assessment periods occurring prior to the intervention, 1 to 2 weeks after completion of the 6-week intervention session, and 12 weeks after the intervention period is complete.

**Conclusion:**

Results from this study will guide the refinement of the intervention program and inform future research among a large and diverse group of wheelchair and scooter users living with MS.

## Abstract 2

Supplemental Digital Content is available in the text

## 1 Introduction

Approximately 25% of individuals living with multiple sclerosis (MS) are unable to ambulate functional distances in their home and are considered to be “non-ambulatory.”^[1,2]^ Emerging evidence indicates that falls are highly prevalent among non-ambulatory people with MS that can result in debilitating injuries. Rice et al^[3]^ studied 44 full-time wheelchair and scooter users living with MS, and found that 75% reported at least one fall in a period of 6 months, and 48% of those who fell sustained an injury. Approximately, 75% of wheelchair and scooter users living with MS report a fear of falling and 66% limit their activities due to these fears.^[3]^ The cycle of disuse and deconditioning has the potential to increase fall risk in this population.

Previous research among ambulatory individuals with MS indicates that falls and concerns about falling can have a substantial impact on community participation and quality of life.^[4]^ Among non-ambulatory individuals with neurological impairments, previous studies have found that being an active member of society is a key factor in preventing health deterioration.^[5]^ Therefore, self-imposed activity restrictions associated with falls and concerns about falling can result in significant consequences beyond any physical injury.^[6,7]^

Our research team recently concluded a pilot fall prevention study to examine the impact of a one-time therapeutic intervention designed to reduce fall incidence among wheelchair and scooter users living with MS.^[8]^ After completion of the intervention, fall incidence significantly decreased (*P* < 0.001), however a small effect size was noted (*d*_*z*_ = 0.26) and no significant difference was found among concerns about falling (*P* = 0.728, *d*_*z*_ = 0.01). Although encouraging, the small effect sizes indicate that the brief intervention may not be comprehensive, and other fall related risks factors need to be addressed. We seek to expand our pilot study by pursuing a multifaceted approach to comprehensively manage fall risk among full-time wheelchair and scooter users living with MS.

The purpose of this study is to perform a structured process evaluation^[9]^ and examine the efficacy and feasibility of a community-based intervention to reduce fall incidence among full-time wheelchair and scooter users with MS. Secondary aims of the intervention are to improve functional mobility skills associated with fall risk (e.g., transfer and wheelchair/scooter skills, balance), increase knowledge of fall risk factors, decrease fear of falling, and enhance quality of life and community participation. We hypothesize that compared with participants who do not have access to the intervention (control group), individuals who have been exposed to the intervention will report decreased fall incidence and present with higher quality transfer and wheelchair/scooter skills, improved sitting balance abilities, increased knowledge of fall risk factors, decreased fear of falling, and report better quality of life and community participation.

## 2 Methods

### 2.1 Study setting

The study will be implemented in 3 diverse settings in an effort to recruit a wide range of participants and examine the feasibility of the study in various settings. Testing will be performed in 2 urban environments: Chicago, IL in association with the University of Illinois at Chicago, and Atlanta, GA in association with Shepherd Center between June 2018 and September 2020. Testing will also be performed in a rural environment, Champaign, IL, in association with the University of Illinois at Urbana-Champaign (UIUC). UIUC will be the coordinating center.

### 2.2 Recruitment

Recruitment will occur through face-to-face interactions during MS events in the community and health care facilities, and by placing advertisements in places and periodicals frequented by individuals living with MS (e.g., doctor's offices, community centers, Momentum Magazine). In addition, participants registered with the North American Research Committee on Multiple Sclerosis (NARCOMS) Research Registry, Shepherd Center clinical research databases, and the Disability Resources and Educational Services Research Registry will be sent information about the study. Over the course of 3 years, we will recruit approximately 20 participants per year in Atlanta and Chicago and 14 per year in Champaign-Urbana.

### 2.3 Participants

Individuals will be recruited for the study if they meet the following inclusion criteria: a diagnosis of MS; age more than 18 years old; patient determined disease steps level of 7 (i.e., main form of mobility is via a wheelchair or scooter); self-reported ability to transfer independently or with moderate or minimal assistance^[10]^ and self-report at least 1 fall in the past 12 months. Participants will be excluded if they have had an MS exacerbation in the past 30 days, receive a score of 10 or above to the short blessed test^[11]^ (a cognition screening tool) and are unable to remain in an upright position for an hour.

### 2.4 Ethics and confidentiality

All study procedures have been independently reviewed and approved by the Institutional Review Board at each study location. Prior to engaging in any research activities, all study participants will have the opportunity to review an informed consent document and ask questions to a trained research assistant about the study details. If in agreement, participants are asked to sign the informed consent document. Participants will be compensated $30 for each of the three study assessments ($90 total). After study participants return their final fall calendar, as described below, he/she will receive an additional $10.

All data will be kept confidential and stored on a password protected computer connected to UIUC's secure server. Paper based documents will be kept in a locked file cabinet at each individual study location.

### 2.5 Trial design

**Figure 1:**
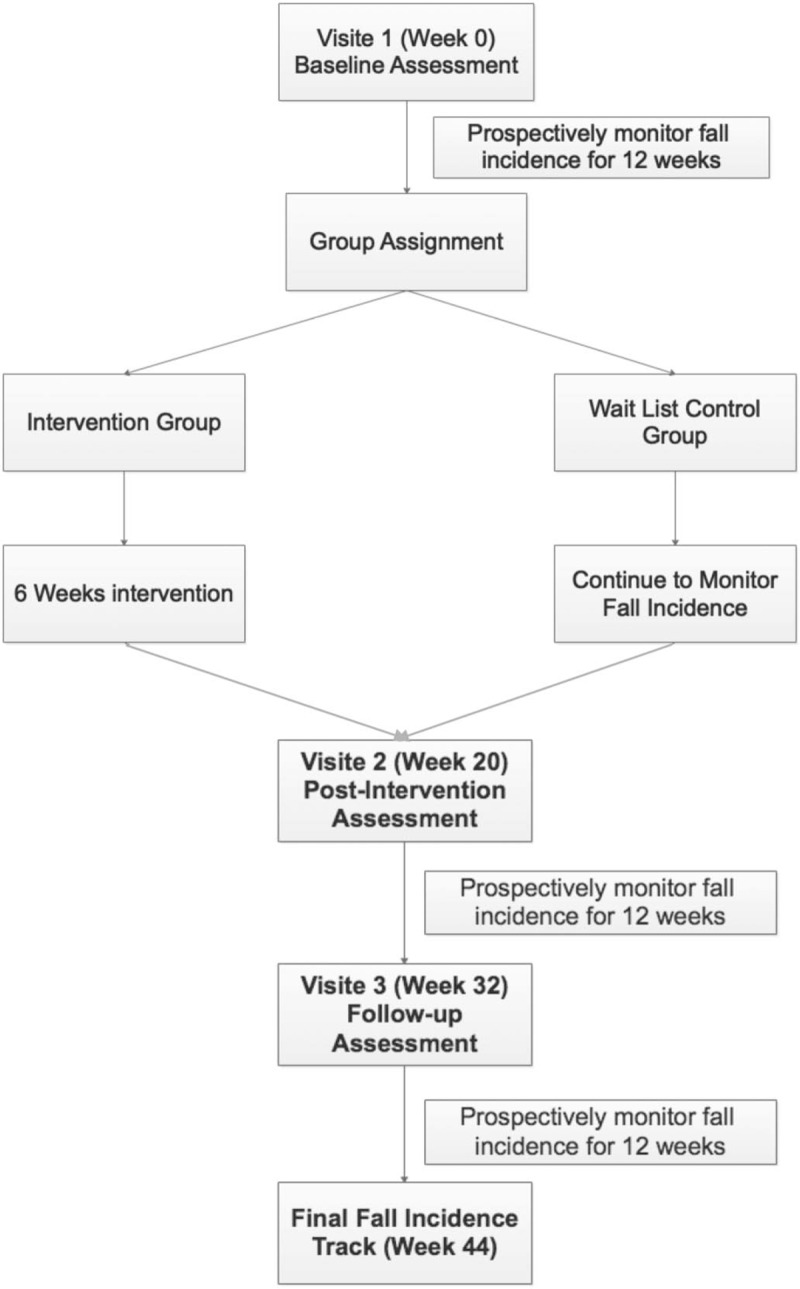
iROLL study design scheme. iROLL = individualized reduction of falls.

To achieve our specific aims, we will implement a non-randomized clinical trial to examine the feasibility and efficacy of the individualized reduction of falls (iROLL) intervention program (ClinicalTrials.gov Identifier: NCT03705364). This trial has been funded by the National Multiple Sclerosis Society (RG1701-26862). The National Multiple Sclerosis Society had no role in the study design. This 6-week community-based intervention program (iROLL), is designed to reduce fall incidence, improve modifiable factors associated with falls, reduce fear of falling, and enhance quality of life and community participation. To evaluate the impact of the iROLL program, participants will be asked to attend 3 study visits over 8 months (Fig. 1). A detailed protocol describing the study is provided in Appendix A. To the greatest extent possible, our research team will follow the standards set by the Standard Protocol Items: Recommendations for Interventional Trials (SPIRIT) statement.

During visit 1, all participants will undergo a baseline assessment. A trained research assistant blinded to study group assignment will ask participants to complete surveys related to fall management, quality of life, and community participation. Participants will also be assessed on wheelchair/scooter and transfer skills and seated postural control. After the first study visit, all participants will be asked to prospectively monitor fall frequency for a period of 12 weeks. After tracking falls for approximately 4 weeks, participants will be assigned to an intervention group (IG) or wait list control group (WLCG). The participant's availability will be collected and a time and dates for the intervention sessions will be established. If the participant's schedule allows for participation, he/she will be assigned to the IG. If not, he/she will be assigned to the WLCG. Participants will be notified over the phone of their group assignments. After assignment, all participants will continue to track fall frequency.

IG participants will be invited to participate in the iROLL program delivered by a physical or occupational therapist and supported by trained research assistants. The iROLL interventionist (trainer) will provide instruction on a variety of topics related to the management of fall risk. Course sessions will be held over 6 consecutive weeks for a period of 2 hours each. All participants will be asked to continue to monitor fall frequency during and after completion of the intervention period. All participants will be re-evaluated 20 weeks (Visit 2) after the baseline assessment utilizing the same protocol to examine the immediate impact of the intervention compared with the WLCG. We will repeat the assessment 32 weeks (Visit 3) after baseline to examine the long-term impact of the intervention. Finally, participants will be asked to track fall frequency for an additional 12 weeks to allow researchers to examine the long-term impact of the program. Fall incidence will be tracked for a total of 24 weeks post intervention, 44 weeks total. The entire study protocol will be implemented in multiple waves over the course of 3 years. Participants allocated to the WLCG during the first 2 and a half years of the study will be given the opportunity to transition into the IG in the subsequent wave to maximize participant recruitment.

### 2.6 Outcomes

#### 2.6.1 Visit 1 (baseline assessment)

The following outcomes will be assessed during Visit 1 by a trained research assistant blinded to study group assignment:

Transfer skills: The ability of a person to transfer from their wheelchair or scooter to an exam table will be evaluated utilizing the transfer assessment instrument (TAI). The TAI has been validated among individuals with neurological impairments, including MS.^[12]^ Participants will be asked to perform up to 4 transfers to/from their wheelchair or scooter to a mat table. Participants will be instructed to perform the transfer in their typical manner and may utilize assistive devices or human assistance, as needed. If human assistance is required, the participant's primary caregiver will also be asked to attend the assessment.
Wheelchair skills: Participants’ ability to control their wheelchair or scooter will be evaluated using the wheelchair skills test (WST).^[13]^ The WST is a well-established measure validated among individuals living with various neurological impairments, including MS. Separate versions of the tool are available for manual, power and scooter users. Participants will be evaluated on a variety of skills ranging from rolling forward and backward to ascending and descending curbs.
Seated postural control: The function in sitting test (FIST) will be used to assess seated postural control. The FIST is a validated clinical measure that rates participants’ ability to perform 14-seated postural tasks.^[14,15]^
Fear of falling: To assess fear of falling, the Spinal Cord Injury Fall Concern Scale (SCI-FCS)^[16]^ will be administered. The SCI-FCS is a reliable and validated measure to evaluate fear of falling in wheelchair users living with spinal cord injury (SCI) and a useful tool to examine the effectiveness of a program to minimize fall incidence.^[16]^ Although originally developed for individuals living with SCI, the items assessed are commonly performed by full-time wheelchair and scooter users with various disabilities. Our research team has utilized this outcome measure during previous research involving individuals with MS.^[17]^ Study participants will also complete a direct assessment of fear of falling.^[18,19]^ Participants are asked: “In general, are you worried or afraid you might fall?” A 4-point scale from “very worried” to “not at all worried” will be used.
Fall prevention strategies: We will evaluate the participant's ability to understand ways to manage fall risk and avoid dangerous environmental situations associated with falls. Participants will be asked to complete the fall management scale^[20]^ and the fall prevention and management questionnaire (FPMQ). The FPMQ specifically evaluates 12 areas of knowledge addressed during the proposed intervention program, such as “I know how to safely get up after a fall.” Items are scored from 0 (“strongly disagree”) to 4 (“strongly agree”). As a knowledge questionnaire matching the program content, this tool captures treatment receipt, an important component of treatment implementation.^[19]^
Community participation and quality of life: Community participation and quality of life will be evaluated to gain an understanding of the impact of the intervention on the day to day lives of study participants. As per the recommendation of the International MS Falls Prevention Research Network, the community participation indicators^[21,22]^ will be used to evaluate participation. The multiple sclerosis quality of life-54^[23]^ measure will be utilized to evaluate quality of life.
Cognition: Cognition will be indexed at each assessment utilizing the brief international cognitive assessment for MS (BICAMS) to examine the influence of cognitive function on a participant's ability to benefit from the iROLL intervention. The BICAMS is composed of the symbol digit modalities test, California verbal learning test-II, and the revised brief visuospatial memory test.^[24]^ Respectively, these validated tests quantify cognitive processing speed and memory which has been linked to frequency of falls in persons with MS.^[25,26]^ Importantly, BICAMS was designed to be used and interpreted by non-psychologists.

#### 2.6.2 Prospective fall monitoring

After completion of Visit No. 1, all participants will be asked to prospectively record fall incidence for 12 weeks prior to the start of the intervention period. Prospective monitoring of fall incidence will be performed to avoid recall bias associated with retrospective reporting of fall incidence. Using the Word Health Organization's (WHO) definition,^[27]^ a fall will be defined as “Inadvertently coming to rest on the ground, floor or other lower level, excluding intentional change in position to rest on furniture, walls, or other objects.” In accordance with the International MS Falls Prevention Research Network guidelines,^[28]^ prospective fall frequency will be captured utilizing a fall diary system for 44 weeks, starting at the first study visit. Participants will be provided a paper calendar and asked to place an “X” on any day they sustain a fall. The definitions of a “fall” will be provided to participants on the calendar. Participants will be asked to provide a short description of the circumstances associated with the fall, if an injury occurred, and if a medical professional was contacted. Participants will be provided with a self-addressed/postage paid enveloped and asked to return the diary on a monthly basis. To assure compliance and diminish the effect of recall bias, follow up phone calls will be made to all study participants every other week to assure they are staying up to date with their fall diary.

#### 2.6.3 Visits 2 and 3 (follow assessments)

All participants will be asked to return to the laboratory 20 and 32 weeks after the first study visit to be re-evaluated utilizing the same protocol. All participants will be asked to continue to complete fall calendars for an additional 12 weeks after the final study visit (44 weeks total) to capture the long-term impact of the program.

### 2.7 Intervention

#### 2.7.1 Program structure

The intervention, implemented by either a physical or occupational therapist and supported by research assistants, involves didactic presentations, interactive group discussions, and practice opportunities utilizing a variety of learning styles (visual, auditory, and kinesthetic). The program has been manualized to support fidelity. A complete listing of the education materials is provided in Appendix B. Each trainer will receive approximately 3 hours of training prior to implementation of the education from the principal investigator. A variety of presentation techniques are integrated, including action planning,^[29]^ handouts informed by health literacy guidelines,^[30]^ videos and pictures to maximize modeling, understanding, and long-term retention of information.^[31–35]^ Although a standardized protocol has been established, consistent with other evidenced-based programs designed to reduce fall risk,^[20,25]^ the goals of individual participants will be discussed and taken into consideration during the education program. In order to assure that participants receive an individualized program, the ratio of participants to instructors will be no >5 participants to 1 instructor. To ensure that participants benefit from the group style approach, a minimum of 2 participants will be required for each group. If a participant requires assistance to perform activities of daily living, his/her caregiver will also be invited to attend the education sessions. Each session will last approximately 2 hours. If a participant misses a study session, materials will be provided to the participant at the next study session. The repetition built into the program will support learning and help participants who have missed a session catch up. However, if a participant misses >3 sessions, he/she will be withdrawn from the study.

### 2.8 Program content

Table 1 provides a description of the topics to be covered during each session. The content of the program is based on peer-reviewed literature describing risk factors associated with falls.

**Table 1:**
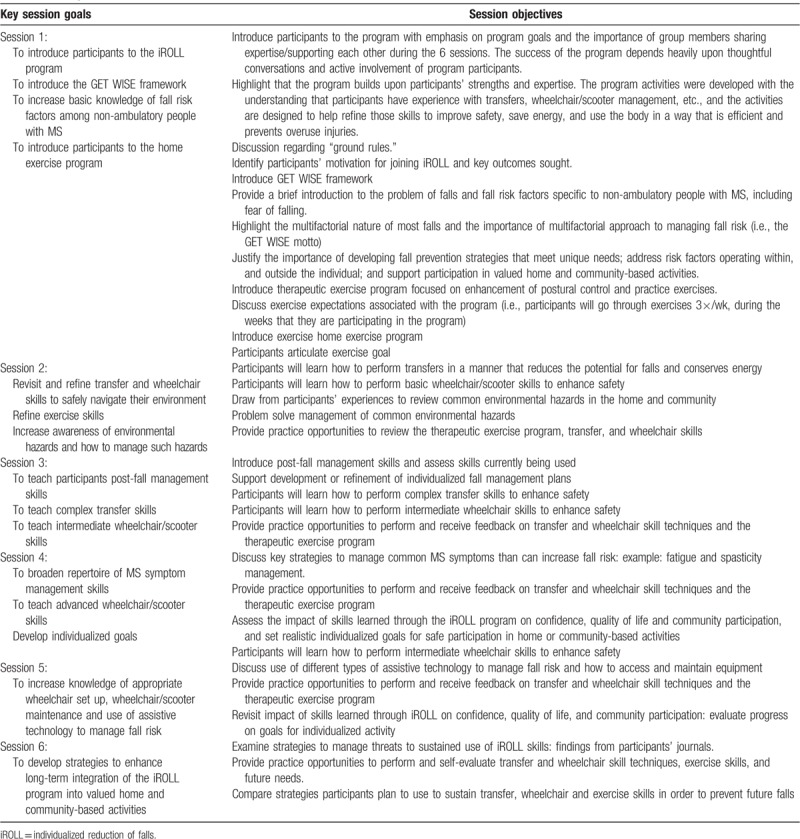
Intervention goals.

#### 2.8.1 Seated postural control

Impairments in seated postural control are frequently associated with falls among wheelchair and scooter users. Individuals commonly fall when trying to reach for an item outside their base of support^[26]^ or shifting their weight on an unstable surface.^[36]^ To manage this aspect of fall risk, participants will engage in a therapeutic exercise program. The program has been designed to strengthen core musculature through the performance of functional activities such as reaching, scooting, shifting weight, etc. For each exercise, participants will learn how to perform the exercise and ways to modify the exercise to make them easier or more difficult based on their own fitness levels or how they are feeling on a particular day. The trainer will provide individualized instructions on the frequency of exercise performance based on the participant's current level of fitness, and participants will be given a video showing how to perform the exercises. Throughout the course of the program, participants will set goals for exercise performance at home and collaboratively work with the trainer to understand and address barriers for correct and consistent exercise performance, with the goal of building participants’ confidence in their exercise skills and sustaining exercise habits. Participants will be asked to keep a log of their exercise frequency.

#### 2.8.2 Transfer skills

Falls during transfer activities are common and often occur due to a lack of knowledge of how to correctly perform the activity.^[37]^ Participants will learn how to perform transfers to and from their wheelchair or scooter in a manner that reduces the potential for falls and conserves energy. Transfer techniques taught to participants will be individualized to the participant's specific needs and functional abilities. After the initial instructions are provided, a video for use at home that models ideal transfer techniques will be provided. Subsequent intervention sessions will provide participants with practice, feedback, and the opportunity to refine transfer performance. Participants will also have an opportunity to discuss challenging transfers and work with the trainer to develop solutions.

#### 2.8.3 Wheelchair skills

Lack of knowledge on how to effectively navigate difficult terrain and obstacles in a wheelchair or scooter is also a common cause of falls.^[38,39]^ Participants will learn how to perform basic and complex wheelchair skills to enhance their safety while navigating both their home and communities. The skills taught will be based on the wheelchair skills program.^[40]^ Participants build skills including, but are not limited to: how to ascend and descent ramps, manage curbs, and navigate rough terrain. Such skills are important to enhance mobility and manage environmental hazards in both an individual's home and community.^[41,42]^ Participants will be encouraged to discuss how the wheelchair skills featured in the iROLL program were used at home and in the community and their impact on confidence and ability to perform desired activities. Video and live demonstrations will be used for initial instruction and practice opportunities will be available for participants to learn and refine skills.

#### 2.8.4 Managing environmental fall hazards

There are many environmental hazards that are exogenous to an individual that can influence fall incidence. Thus, participants will learn strategies to minimize the impact of such hazards, with emphasis placed on strategies that can be employed during transfers and during wheelchair or scooter use in the home and community. For example, participants will be educated on the appropriate use of structural items such as grab bars and handrails.^[43]^ Additionally, participants will draw from their own fall experiences to discuss common environmental hazards. Mitigation strategies that feature development of realistic action plans to address environmental hazards that utilize available resources and evaluation of goal attainment will be utilized.^[44]^ Participants will also be asked about specific environmental hazards they encounter, and the group trainer will help the individual problem solve through these specific challenges.

#### 2.8.5 Management of falls

While the focus of the program is to prevent falls, previous research indicates that delayed initial recovery (i.e., lying on the ground for ≥10 minutes after a fall occurs) is common.^[45,46]^ Therefore, participants will develop post-fall management skills. Specifically, participants will learn how to safely get up off of the ground, communicate with care partners during emergency situations, and develop “check-in” systems involving friends and family in order to prevent an individual from lying on the floor for an extended period of time after a fall occurs. The importance of seeking medical services when an injury to the head occurs will be emphasized. Participants will have an opportunity to develop an action plan for fall management and will have an opportunity to discuss their plans with the trainer and group.

#### 2.8.6 Impact of MS symptoms on falls

The unique symptoms associated with MS including fatigue, muscle spasticity, muscle weakness, impaired vision, etc. have an impact on fall risk.^[19]^ The iROLL program is designed to increase participants’ ability to identify and manage MS symptoms that can increase fall risk during transfers, exercise, and wheelchair use and other activities. For example, participants will learn how to conserve their energy and utilize assistive technology whenever possible to prevent extreme fatigue. Discussions will be held to allow group members to discuss challenges and share strategies.

#### 2.8.7 Assistive technology to manage fall risk

Improper set up^[39,47]^ and a lack of appropriate maintenance^[25,37,38]^ of assistive technology are known risk factors for falls among wheelchair and scooter users. Participants will learn about a variety of assistive technology resources available to them, from simple, and inexpensive devices, such as transfer boards to more complex items such as power wheelchair seat elevators. Participants will also learn how to: access the technology (including funding options), utilize key pieces of technology appropriately (e.g., appropriate placement of a transfer board during a level transfer), and how to maintain the technology correctly in order to prevent falls associated with equipment.^[48]^

### 2.9 Process evaluation

A comprehensive process evaluation informed by Moore et al,^[9]^ and focusing on implementation, mechanism of impact, and context has been planned.

To examine implementation of the intervention, which includes fidelity, dose, adaptation, and reach,^[9]^ several procedures have been put into place. Specifically, the trainers will be asked to complete a form after each intervention session to document the start and end times of the session (dose), indicate which of the session activities essential to the program were covered during the session (fidelity), and provide feedback, including details about any changes made to planned content or process (adaptation). The research team members who led development of the intervention came to consensus on the intervention activities essential to program fidelity through an iterative series of conversations. The “core” activities identified were then included on the form completed by trainers at the end of each intervention session. Fidelity will be assessed by a trained research assistant by calculating the percentage of completed “core” activities out of the total number of “core” activities for each session. Fidelity will also be evaluated through post-session feedback from iROLL participants. After each of the 6 iROLL sessions, study participants will be asked to complete a feedback form to evaluate the extent to which specific objectives associated with each individual session were met.

Dose will be summarized by calculating the duration of each session based on start and end times. Adaptations to the iROLL program, captured through documentation provided by trainers after each session, will be monitored, logged and summarized by a trained research assistant throughout the duration of program delivery. Reach will be monitored by a study coordinator with a log providing a record of interested participants, those completing the program and those dropping out of the program. When available, reasons for attrition will be documented by study staff.

Mechanisms of impact will be examined via participant and trainer feedback obtained over time, through several purposeful strategies. For example, upon the conclusion of the final program session, study participants will be asked to complete a comprehensive evaluation of the intervention that includes open ended questions to examine strengths and weaknesses of the program. All participants who have completed at least 3 intervention sessions will also be asked to participate in a post-intervention semi-structured interview to qualitatively explore how the iROLL program influenced participants’ fall prevention behaviors. The interview will be conducted by a member of the study team within 1 month of the study conclusion for the specific purpose of gaining participant insights into how the program worked.

In addition to documenting insights after each session and completing a comprehensive post-intervention evaluation of the program that includes items designed to yield insight regarding how the iROLL program worked or did not work, a subset of trainers (selected by convenience) will participate in a semi-structured phone interview. The interview will be conducted by a member of the study team within 1 month of the study conclusion for the specific purpose of gaining trainers’ insights into how the program worked. The post-course evaluation is a key opportunity for trainers to document their insights regarding barriers and facilitators and how context impacted the iROLL program.

The data gathered from both study participants and trainers will be used to develop a comprehensive summary of program strengths, weaknesses, and recommendations for improvement in future iterations of the program.

#### 2.9.1 Wait list control group participants

During the intervention period, participants allocated to the WLCG will continue to receive phone calls from the research team every other week. During those calls, research team members will inquire about fall incidence, and remind participants to keep up with their fall diaries. Otherwise, WLCG participants will be asked to continue their normal activities, as per the typical standard of care.

### 2.10 Feasibility

To examine the feasibility of delivering and evaluating the intervention, as recommended by Thabane et al,^[49]^ our research team will examine recruitment and retention rates of study participants, adherence to the proposed program, safety of the program, and the ability to collect primary and secondary outcomes.

### 2.11 Data collection and management

Data collected during study visits 1, 2, and 3 will be collected by trained research assistants who will be blinded to each participant's study group assignment. All research assistants will receive training on the proper implementation of the assessment protocol from the principal investigator and be asked to demonstrate their skills during a mock trial in order to ensure consistency and accuracy. Study protocols, scripts, check-lists for study visits, and follow up phone calls and consent forms have been included in the iROLL manual of procedure (Appendix A) and will be provided to each research assistant.

All data collected during assessment visits 1, 2, and 3 will be collected using paper forms and entered into a database maintained at UIUC. Data will be entered by a trained research assistant and independently verified by a second assistant. Data analysis will be performed by a statistician blinded to group assignment.

### 2.12 Sample size estimation

To examine the influence of the iROLL program on our primary aim, reduction in fall incidence, we will recruit 160 participants over a period of 3 years. We have based our recruitment goal on a power analysis utilizing pilot data collected by our research team. Power analysis for an independent two sample *t* test was conducted in G∗Power^[50]^ using an alpha level of 0.05 and a power of 0.8. Results indicate that a total of 128 participants (64 per group) are needed to detect a significant difference between participants who have received the intervention and those who did not with a medium effect size (Cohen *d* = 0.50) and two tails. An additional 32 participants will be recruited to account for an approximate 20% participant drop-out rate. The 20% drop-out rate is a conservative estimate based on previous studies performed by our research team. Higher effect sizes are expected for other variables of interest, including transfer quality, seated postural control. Subsequent power analysis of these variables indicates that our trial will be appropriately powered.

### 2.13 Data analysis

The data will be examined for normality violations using a Shapiro-Wilks test. The assumptions of homogeneity of variance and homogeneity of covariance will be examined with Box M and Mauchly W tests, respectively. To examine the presence of outliers, histograms and Q–Q plots will be developed.

To examine the differences between IG and WLCG participants, propensity score analysis will be utilized.^[51,52]^ Propensity score analysis is a method to adjust for non-randomized bias in clinical trials. Propensity score stratification method seeks to create subgroups that include carefully matched subjects such that confounding covariates are equally distributed among study participants. Specifically, for each participant, a logistic regression model will be performed with the participant's intervention being the dependent variable and the baseline characteristics being the predictors. Propensity score will be calculated as the predicted probability of getting IG from the logistic regression. Participants will then be divided into 5 equal-size mutually exclusive subclasses using quintiles of the estimated propensity score.^[53]^ While the first subclass includes participants who are least likely to be in IG, the last subclass are participants who are most likely to receive IG. To examine the homogeneity of the selected confounding covariates between intervention groups among subclasses, 2-way analysis of variance will be used for continuous variables and chi-square tests will be used for categorical variables. The non-significance of the interaction between subclass and intervention will indicate that using propensity score to place participant into groups of similar likelihood is successful. The intervention effect will be examined separately within each stratum and the confounding effect of baseline covariates will be eliminated from the analysis, using the mixed-effect method described below. Heterogeneous intervention effects across these 5 propensity strata will be testing using *Q*-statistics in which each estimate is weighted by the reciprocal of the square of standard errors. The stratum-specific intervention differences will then be combined and the overall treatment effect will be estimated by computing the Cochran-Mantel-Haenszel weighted average.

To examine the impact of the proposed intervention using quantitative data, a linear mixed-effects model will be used to determine predicted mean values at each assessment point and to test the study hypotheses with respect to between-group differences over the entire study period. BICAMS data will be utilized as a covariable to examine the influence of cognition on the participant's ability to benefit from the iROLL program. A customized PROC MIXED program will be developed (SAS, Inc.; Cary, NC) and will be applied to the continuous outcomes (e.g., fall incidence, seated postural control, etc.). To evaluate categorical outcomes (e.g., yes/no fear of falling questions, etc.), a customized PROC GLIMMIX program will be developed and carried out. PROC MIXED/GLIMMIX account for within-subject covariability and will utilize all available data. In each linear mixed-effects model, time (coded as 0, 1, and 2 for week 0, 20, and 32, respectively) and study group (IG and WLCG) will be included as fixed effects, with linear time and time-by-intervention group interaction terms. In all models, random effects included intercept and linear slope terms, and an unstructured covariance will be used to account for within-subject correlation over time. A significant interaction term will indicate that a difference exists among the outcomes across time between intervention groups. If the coefficient of the interaction term is not significant, a model omitting this term will be fitted and the main effect of time and of the intervention will be evaluated.

Effect size (Cohen *f*^[2]^) will be calculated and utilized to power future large-scale interventions. To prevent a Type I error, post-hoc analyses with a correction of alpha will be performed when appropriate. The data collected here will allow for power calculations to determine sample sizes for future work on the association between falls and activity curtailment among older adult full-time wheelchair and scooter users.

To examine the qualitative data yielded through the participant and trainer post-course interviews, a thematic analysis will be conducted as outlined by Braun and Clarke.^[54]^ This step-by-step process includes familiarization with the raw data, generating initial codes, searching for and identifying themes, reviewing and refining themes, and defining and naming the themes. The interviews will be audio recorded and a verbatim transcript created. Two members of the research team (JS and TV) will read all the interviews and then individually code the responses independently. After performing the initial coding, the research team members will discuss the codes until a consensus is reached on meaning. Summary reports will be developed and will be used by the research team to inform future program improvements and better understanding of how the intervention led to, or did not lead to, intended outcomes.

## 3 Discussion

We propose to examine the feasibility and efficacy of a community-based intervention to reduce fall incidence among full time wheelchair and scooter users with MS. The intervention is unique in its careful attention to addressing wheelchair and transfer-related influences on fall risk. This study will establish a foundation for evidence-based fall prevention practice in community-based or inpatient rehabilitation settings specifically for full-time wheelchair and scooter users living with MS, and will directly address limitations in the existing MS fall prevention literature.

The proposed intervention has good potential to improve the health and well-being of a frequently underserved segment of the MS population with the ultimate goal of enhancing quality of life and community participation. Given the adverse impact of falls, a considerable amount of research has focused on fall prevention in recent years. There is emerging evidence that falls can be minimized in persons with MS with targeted interventions.^[55–58]^ However, this research has almost exclusively focused on individuals who are ambulatory. This focus ignores the approximate 25% of the MS population^[1,2]^ who are non-ambulatory. Due to the differences in physical characteristics (e.g., muscle strength and balance impairments) and functional mobility limitations (e.g., assistive devices utilized) risk factors for falls are distinct for individuals with MS who are non-ambulatory compared with those who ambulate. Minimal research has been performed to examine fall management strategies among wheelchair and scooter users living with MS.^[59]^

On both a physical and psychological level, falls can be extremely detrimental and have a significant impact on the health and well-being of people with MS.^[4]^ Decreasing fall incidence, improving functional mobility and enhancing fall related knowledge has strong potential to improve overall health, well-being, and quality of life among wheelchair and scooter users living with MS. This study also has the potential to provide a direct benefit to the MS community is a short time frame. During the course of this 3-year study, an evidenced based intervention program will be evaluated and refined to serve the needs of wheelchair and scooter users living with MS.

Through the course of this study, feedback will be obtained from both study participants and the trainers who are implementing the study intervention. Utilizing the guidelines set by the Medical Research Council,^[9]^ a formal process evaluation will be conducted to systematically examine implementation, mechanisms of impact and context. Given the novelty of the program, this comprehensive assessment will provide important insight into the quality of the program and areas in which improvements are necessary. Results will be utilized to make recommendations on global and location- specific changes. For example, the urban, hospital-based location may face different challenges compared with the university based rural location. Specific recommendations for improvements will also be made on a global scale and specific to the environment in which the intervention will be implemented.

### 3.1 Study limitations

This study is not without limitations. Most significantly, participants are not being randomly assigned to the IG and WLCG. While random assignment is the gold standard, after careful consideration of the challenges associated with recruiting wheelchair and scooter users with MS, our research team did not view random study group assignment to be feasible. Our previous experience in research and clinical settings made us aware that many of the potential study participants face challenges related to accessible transportation, and require support from others (e.g., family members, community-based transportation services serving people with disabilities) to attend an intervention program of this nature. Participants also indicated that fatigue limited their engagement in intervention programs during specific times of the day. In addition, to facilitate access to peer support, which was essential to the intervention, at least 2 participants must be randomized to the IG group at the same time to form a group. Due to the challenges related to recruitment, achieving this threshold is difficult. In an effort to make the program available to as many participants as possible, gather sufficient data to examine the influence of the intervention, and conduct a process evaluation, non-random group assignment was found to be the most logical protocol. Strengths of the study however, such as the 3-month prospective fall monitoring period prior to the start of the intervention and continued prospective fall monitoring 6-months post intervention will further help our research team to gain a good understanding of the strengths and weaknesses of the intervention program and the influence on management of falls. A matched pair analysis will be conducted to examine the influence of the intervention compared with the standard of care.

In addition, study participants will be asked to perform the exercise program at home without oversight and will be expected to perform a variety of study related activities (e.g., implementation of fall recovery plans and home modifications) independently. Finally, our research team will rely on self-report of study participants to collect fall frequency data and determine if the home-based activities assigned to participants were performed.

## 4 Summary

This study serves to examine the feasibility and efficacy of a community-based intervention to reduce fall risk among full-time wheeled mobility users with MS. Careful examination of the intervention is a critical step needed to support evidence-based fall prevention efforts for wheelchair and scooter users with MS to promote safe use of wheelchairs and scooters for everyday functioning.

## Author contributions

**Conceptualization:** Laura Rice, Elizabeth W. Peterson, Deborah Backus, Jacob J. Sosnoff.

**Data curation:** Sa Shen.

**Formal analysis:** Sa Shen.

**Funding acquisition:** Laura Rice, Elizabeth W. Peterson, Deborah Backus, Sa Shen, Jacob J. Sosnoff.

**Investigation:** Laura Rice.

**Methodology:** Laura Rice, Elizabeth W. Peterson, Deborah Backus, JongHun Sung, Toni Van Denend, Sa Shen, Jacob J. Sosnoff.

**Project administration:** Laura Rice, Elizabeth W. Peterson, Deborah Backus, Jacob J. Sosnoff.

**Resources:** Laura Rice.

**Software:** Sa Shen.

**Supervision:** Laura Rice, Elizabeth W. Peterson, Deborah Backus, Jacob J. Sosnoff.

**Writing – original draft:** Laura Rice, Toni Van Denend, Sa Shen.

**Writing – review & editing:** Elizabeth W. Peterson, Deborah Backus, JongHun Sung, Rebecca Yarnot, Libak Abou, Jacob J. Sosnoff.

Laura Rice orcid: https://orcid.org/0000-0003-3902-1151.

## Supplementary Material

Supplemental Digital Content

## Supplementary Material

Supplemental Digital Content
