# Supplementary material for: Validation of an individualized reduction of falls intervention program among wheelchair and scooter users with multiple sclerosis
Source: Medicine (Baltimore). 2019 May 13;98(19):e15418. doi: 10.1097/MD.0000000000015418 (PMC6531239; doi:10.1097/MD.0000000000015418)
Supplement: Supplemental Digital Content [file medi-98-e15418-s001.doc]

**Appendix A: iROLL Study Protocol**

As study participants express an interest in the study an effort will be made to create cohorts of participants of 2 or more individuals. As a result, a participant may have to wait to begin the study. If after 2 months, if no other participants in the same geographic area express an interest, the participant will be automatically assigned to the weight list control group (WLCG). The single participant will proceed as noted below, however he/she will be not assigned to a study group.

If 2 or more participants express an interest in the study during a period of 2 months, the procedures as noted below will be followed.

Prior to Participant Assessments:

1. Obtain an informed consent document, assessment packet, BVMT booklet, participant survey packet, and post-assessment materials
2. Assessment packets will include,
   1. Cover Page (title, participant ID, date, Manual/ Power)
   2. BICAMS
      1. Symbol digit modalities test (SDMT)
      2. California Verbal Learning test-II (CLVT-II)
      3. Revised Brief Visuospatial Memory Test (BVMT)
   3. Transfer Assessment Instrument
   4. Function in Seating test (FIST)
   5. Wheelchair skill test (WST-M or WST-P)
   6. Post Assessment Tasks Page

*note, separate packets will need to be made for WST-M and WST-P, distinctions will be made clear on cover page

1. Survey packets will include,
   1. Demographic information
   2. Spinal Cord Injury- Fall Concern Scale (SCI-FCS)
   3. Fear of Falling Outcome Measures
   4. Fall Management Scale (Fall Incidence and injury survey)
   5. Fall Prevention and Management Questionnaire (FPMQ)
   6. Community Participation Indicator (CPI)
   7. Multiple Sclerosis Quality of Life-54
2. Post-Assessment materials will include, in order:
   1. Fall diary (x8)
   2. Self-addressed envelope with postage (x8)
   3. Manella envelope for contents

Assessment Preparations

1. Retrieve informed consent document, appropriate assessment packet (WST-M or WST-P), BVMT booklet, participant survey packet, and post-assessment materials
2. Ensure all pages in packets are accounted for (see list above) prior to participant arrival.
3. Ensure gait belt, a clipboard, blank paper, and a pencil are ready and available.
4. Ensure that a taped square is ready on the floor for WST.

Baseline Assessment- Visit #1

1. Once participant has arrived, prior to assessment:
   1. Explain Informed Consent and if the participant agrees to participate, have them sign informed consent.
   2. Research assistant will make a copy of the consent and give to study participant if participant desires a hard copy.
2. Using the assessment packet, ensure the cover page is completed with participant ID date, and visit number.
3. Remind participant that if at any time they are not comfortable with completing a task they are asked to do, just inform us and we can skip that task.
4. Explain to the participant that next, we will be completing a series of short tests that will assess their cognition and memory.
5. Ask participant to pull up to the table for the BICAMS.
6. Move to the BICAMS in assessment packet.
7. Beginning with the SDMT follow script on administration page.
8. Get a timer ready with 90 seconds.
9. Cue participant to begin.
10. Record participant responses indicating whether they were correct or incorrect in investigator packet.
11. Once timer goes off, cue participant to stop.
12. Once the SDMT has been completed, return all papers to packet.
13. Tell participant that the next test is meant to test their verbal learning/ memory.
14. Turning to the CVLT-II, follow instructions to complete assessment.
15. Notify participants that an audio recording device will be turned on.
16. Ensure audio recording device is on prior to beginning trial 1.
17. Play recording of words for participant, cue them to begin after the list is read.
18. Record participant’s responses directly on scoring paper. Cue for more only once.
19. Repeat list 5 times, following instructions on CVLT-II.
20. End audio recording and ensure that it is saved.
21. Notify participants that an audio recording device is turned off.
22. Once the CVLT-II is complete, turn to the next page for BVMT.
23. Based on randomization sheet, note which trial the participant will be using.
24. Following the BVMT script, explain to the participant that the next task is meant to test their visual/special memory and they will be shown a page with 6 shapes on it for 10 seconds. During that 10 seconds they should try to memorize both the shapes and their locations on the page. After the 10 seconds, they will be asked to recreate the image by drawing it out to the best of their ability. They will have no time limit so please draw carefully and to the best of their ability. They will be doing a total of 3 trials using the same form.
25. After explaining how the test works, tell participant you will begin with the first trial.
26. Get timer ready with 10 seconds.
27. Cue participant to begin by showing the shapes page and starting the timer.
28. When timer goes off, hide form and give participant the trial 1 form. Tell participant to recreate the images using the provided paper.
29. Once the participant has completed their recreation, collect response and put it in the correct section of the packet
30. Repeat steps 27-32 two more times using the same form.
31. Once all trials are completed, ensure all participant BICAMS papers are returned to packet.
32. Once the BICAMS is completed, we will ask them to complete the following surveys:
    1. Demographic Survey
    2. Patient Determined Disease Steps *(Note: this should be gone through with participants to ensure correct selection)*
    3. Spinal Cord Injury Fall Concerns Scale (*Note: Tell participants that although this was developed for individuals with SCI, the items are applicable to individuals with MS and no other appropriate measure is currently validated)*
    4. Fear of Falling Outcome Measure
    5. Fall Management Scale
    6. Fall Prevention and Management Questionnaire
    7. Community Participation Indicators
    8. MS Quality of Life 54
33. Indicate that we would like them to complete all surveys on their own, but if needed, they can ask for help.
34. Provide participant with survey packet, pencil, and clipboard
35. Once all surveys are completed, take back packet and review to ensure completion.
36. While reviewing, an assistant of the same gender should help the participant properly put the gait belt of appropriate size on. Indicate that this has been completed in investigator packet.
37. Explaining that this is to make spotting the participant as they complete transfers easier and more efficient. (Spotter will be near participant during all physical tasks)
38. Next, tell participants that we will be completing a series of transfer and postural control assessments.
39. While using TAI assessment, ask participant to transfer themselves to mat table.
40. Once the participant is on the table, begin the FIST.
41. Explain to participant that the next assessment will be used to test postural control.
42. Conduct FIST assessment using scripted dialog and scoring sheet.
43. After the FIST is completed, refer back to TAI part 1 and ask the participant to transfer back into their chair following script.
44. While transfer is happening, complete second transfer measure on TAI.
45. After transfer, offer participant a break for as long as necessary.
46. Once participant indicates that they are ready, ask the participant to again transfer to the mat table.
47. While transfer is happening, complete third transfer measure on TAI.
48. Briefly refer to the WST and ask participant if they can breakdown their chair. If yes, ask them to demonstrate and denote score on WST. If no, tell them “no problem” and move on with assessment. Denote score on WST.
49. Return to TAI to complete final transfer.
50. Once participant indicates that they are ready, ask the participant to again transfer to their chair using TAI script.
51. While transfer is happening, complete fourth transfer measure on TAI.
52. Offer participant to break for as long as necessary before moving on.
53. During this break, complete part 2 of the TAI (once all part 1 scoring is complete).
54. Tell participant that we will be moving around the building to complete the next task.
55. Explain to participant that the task will assess their wheelchair skills. Explain that they will be asked to complete a series of wheelchair tasks and if they do not feel comfortable or are unable to complete any of the tasks, then to let us know and the task can be skipped.
56. Using the modified WST (manual/power) and script, asked participant to complete the listed tasks.
57. While participant is completing tasks, fill out WST scoring sheet.

*end of assessment measures*

1. Explain to the participant that for the next (approximately) 8-12 weeks they will be asked to keep a “fall diary”.
2. Give participant the fall diaries, which will be monthly paper calendars with directions, as well as self-addressed/postage paid envelopes for the duration of the 12 weeks.
3. Explain to participants that they will need to keep track of their falls throughout the duration of the study utilizing this “diary” by placing an “X” on any day during which they sustain a fall and an “O” on any day during which they. Sustain a near fall.
4. Review with participant our definition of a fall: an event where you unintentionally come to rest on the ground or a lower level, and near fall: an occasion in which you felt that you were about to fall but did not actually fall.
5. Explain to participant that on days which falls or near falls occur, they will also need to provide a short description of the circumstances associated with the fall, if an injury occurred, and if a medical professional was contacted.
6. Explain to participants that the envelopes provided are for them to return the fall calendar/diary to the lab on a monthly basis.
7. Inform participant that they should be expecting follow-up calls every other week from a member of the lab to check in with them and make sure they are continuing to complete and return their fall diary.
8. Ask the participant if they have any further questions before we finish.
9. Response to any question to best of ability using understanding of the study.
10. Explain that after 4 -8 weeks they will be sorted into either an intervention group or a wait list group and we will contact them via phone to inform them of which and the next steps for their designated group.
11. With participant, set up next in-person meeting for the re-assessment (range 16-24 weeks).
12. Thank participant for their involvement in the study, notify that they will receive a gift card/ code via email (verify email)
13. Escort participant them out of the testing area.
14. Once participant has left, ensure all materials are in order (both assessment packet, with all of participant’s additions for BICAMS, and survey packet)
15. Put packets in participant file folder and file in appropriate cabinet for later data entry.

Fall Diary Monitoring Call:

1. Prior to calling participant, ensure that you have a “fall assessment script” ready.
2. Following call schedule- every other week, call participant using preferred phone number.
3. Complete fall assessment script form while on the phone with the participant.
4. Remind participant to continue completing their fall diary and mail back at the end of each month.
5. Once off the phone, ensure participant ID and date are on the “fall assessment script”.
6. Put the “fall assessment script” in the participant’s file folder.
7. Return participant’s file folder to appropriate cabinet.

Approximately 2 weeks after Visit #1, the study coordinator will contact the participants to gather information on general availability for times/dates of the study intervention. The coordinator will suggest a series of dates for the intervention that will start approximately 3 months after the end of the first study visit. After the coordinator has gathered information from all study participants in the current cohort, he/she will find a time and dates that will be appropriate for the greatest number of participants.

After one month of tracking falls, participants will be notified of the time and dates of the intervention. Assignments will be made by an investigator not involved with the study assessments and will be based on the participant’s availability to attend the intervention program. If a participant is able to engage in the scheduled time/dates of the intervention, he/she will be enrolled in the intervention group (IG). After attempting to find an intervention cycle that accommodates the participant’s schedule, if a participant cannot participate in the intervention due to scheduling conflicts, the participant will be allocated to the wait-list control group (WLCG). Participants will be notified over the phone if they have been assigned to the intervention or control group.

For participants that have been placed in the IG:

1. Explain to participant that they will be receiving the individualized reduction of falls (iROLL) intervention.
2. Tell participate that they will need to continue to complete their fall diary as they have done for the previous 8-12 weeks.
3. Explain to participant that the iROLL program will require them to participate in 6 weekly group-based education courses at (location).
4. Tell the participant the date and time of the first meeting.
5. Confirm that this date and time works for the participant.
6. Have participant repeat back the date, time, and location to ensure that they know when they need to be at the education course.
7. Remind participant that they will need to return to the lab on (date) for their re-assessment (range 16-24 weeks) of the same tasks performed at their baseline assessment.
8. Confirm that this date and time still works for the participant.
9. Have participant repeat back the date, time, and location to ensure that they know when they need to be at for re-assessment.
10. Ask participant if they have any questions at this time.
11. Answer any questions to the best of ability based off knowledge of the study.

For participants who are not able to participate in the intervention and who are placed in the wait list control group (WLCG), a member of the research team will:

1. Explain to participant that they have been placed on a wait-list for the iROLL program.
2. During their time on the wait list, we will need them to continue to complete their fall diary as they have done for the previous 8-12 weeks.
3. Explain that they will continue to receive bi-weekly calls to check in and ensure they are continuing to complete and submit their fall diaries.
4. Advise participant that they should continue their normal activities during this wait list time.
5. Remind participant that they will need to return to the lab on (date) for their re-assessment (range 16-24 weeks) of the same tasks performed at their baseline assessment.
6. Confirm that this date and time still works for the participant.
7. Have participant repeat back the date, time, and location to ensure that they know when they need to be.
8. Ask participant if they have any questions at this time.
9. Answer any questions to the best of ability based off knowledge of the study.

IG participants will be invited to participate in iROLL intervention delivered by a licensed Physical or Occupational Therapist and supported by research assistants trained by Drs. Rice, Backus or Peterson. The interventional clinician (aka “Trainer”) will deliver the iROLL intervention, which includes instruction on a variety of topics related to the management of fall risk including transfer and wheelchair skills, exercises to improve seated balance, equipment management, environmental factors associated with falls, general knowledge related to MS and fall risk and fall recovery strategies. Intervention sessions will be held over six consecutive weeks for a period of 2 hours each. WLCG participants will be asked by a member of the research team to continue their normal activities in the community (current standard of care).

Intervention Quality Assurance /Fidelity Assurance Protocol

To examine the quality of the iROLL intervention, including fidelity, the following procedures will occur prior to, during and after the iROLL intervention, as noted below:

Prior to Session 1: The principal investigator (PI) based at UIUC will conduct training sessions with all the trainers (i.e., interventionists delivering iROLL).

The purpose of the training sessions is to prepare the trainers to deliver the iROLL intervention and to explain quality assurance procedures. As part of the training, the trainers are told that their roles as trainers includes (but is not limited to) a)completing the post-training Training Feedback Form; b) completing iROLL Trainer Fidelity Forms at the conclusion of each of the 6 iROLL sessions and c) completing an iROLL Trainer Feedback Form, to provide feedback on the overall iRoll intervention, at the conclusion of the final (6^th^) iROLL session.

The PI will be responsible for the training sessions with all the trainers regardless of where a trainer is based, i.e., University of Illinois at Urbana Champaign (UIUC), University of Illinois at Chicago (UIC) or the Shepard Center in Atlanta. The training sessions will be conducted remotely via video-chat or in person. After completion of the training sessions, the trainers will be asked by PI to complete a paper-based Training Feedback Form to examine the quality of the initial training. No identifiable information will be collected on the form, However the study location will be indicated (UIUC, UIC or Shepherd Center). The PI will review the form and make changes to future training sessions based on the feedback obtained. If significant issues are noted on the form, the PI will schedule another training session with all the trainers to address the concerns.

Procedures Occurring *During* the iROLL Intervention

1. Individual session participants & trainers program session evaluation protocols.

At the end of each intervention session (1, 2,3,4,5, & 6):

- Participants will be asked to complete a paper–based individual session evaluation form.
  - Trainer(s) will hand out the form and a pre-paid and pre-addressed envelope to the participants.
  - The trainer will explain what the form is, i.e. an evaluation of the session, and explain that completing the evaluation is voluntary.
  - The form will be filled out anonymously, however the study location (UIC, UIUC or Shepherd Center) will be noted on the form.
  - The trainer will stay in the room where the participants are, but step away from participants as they complete the form, in order for the participants to have privacy as they complete the session evaluations.
  - After filling out the form, participants will be asked by the trainers to put their forms in a pre-paid and pre-addressed envelope, and seal the envelope
  - Trainer(s) will collect all the sealed envelopes.
- Immediately following each iROLL session , each Trainer will complete a paper-based individual session fidelity form. Trainers were told about the fidelity form as part of the aforementioned training session (for trainers) conducted the study PI
  - The form will be filled out anonymously, however the study location (UIC, UIUC or Shepherd Center) will be noted on the form.
  - After filling out the form, the trainer(s) will put their forms in a pre-paid and pre-addressed envelope and seal the envelope.
- The trainer(s) will drop all the collected envelopes to a mail box and these forms will be sent to the Principal Investigator, Laura Rice.

1. Course Evaluations Completed by Participants and Trainers

At the end of the session 6:

- Participants will be asked to complete an iROLL Participant Course Evaluation upon the conclusion of Sessions 6 (i.e., the final iROLL session) after the Session 6 evaluation is completed.
  - The trainer will explain that (a)the course evaluations will be used by the Principal Investigator to learn about the strengths and limitations of the iROLL intervention, and inform decisions about program improvements; (b)a participant’s name will not occur on the form; c)completing the evaluation is voluntary.
  - Trainer(s) will hand out the forms and pre-paid and addressed envelopes to the participants.
  - The form will be filled out anonymously, however the study location (UIC, UIUC or Shepherd Center) will be noted on the form.
  - After filling out the form, participants will be asked to put their forms in a pre-paid and pre-addressed envelope and seal the envelope.
  - Trainer(s) will collect all the sealed envelopes.
  - Trainers will complete an iROLL Trainer Feedback Form. These forms are used to gather trainers’ overall impressions about the iROLL program.
  - After filling out the form, the trainer(s) will put their forms in a pre-paid and pre-addressed envelope and seal the envelope.
  - The form will be filled out anonymously, however the study location (UIC, UIUC or Shepherd Center) will be noted on the form.
  - The trainer(s) will drop all the collected envelopes in a mail box and these forms will be sent to the principal investigator.

Procedures Occurring *After* the iROLL intervention

Overview:

A UIUC-based research team member will invite all IG subjects from UIC, UIUC, and the Shepard Center who attended and completed at least 3 iROLL intervention sessions, to participate in a post-intervention interview that will be conducted either over the phone or in person. The interviews will be conducted by a trained member of the research team. The interview will take place within 3 weeks of completing the intervention, preferably 24 to 48 hours after completing the iRoll intervention.

Purpose:

The purpose of this post intervention interview study is twofold: 1) to investigate the participants’ thoughts and feedback about the intervention program and 2) to further examine the influence of the intervention program on the participant’s fall prevention behaviors. The information obtained from the interview will provide further insight into the areas of strength of the intervention and areas in which improvements are needed. In addition, the findings will help to inform future development of the intervention program.

The interview will take place within 3 weeks of completing the intervention, preferably 24 to 48 hours after completing the intervention.

A UIUC-based research team member will schedule a one-on-one interview on the phone. A member of the research team will contact the participants who have consented to the post-intervention interview by phone and schedule the time/date of the interview.

Interview and Post-Interview Procedures:

The interview will be recorded by a research assisstatant using a digital voice recorder. Phone interviews will take place in a private setting in order to ensure confidentiality. The interview will last approximately 20-30 minutes.

After completion of interview, all interviews that are audio recorded will be transcribed verbatim by a trained member of the UIUC research team.

The 2^nd^ re-assessment will utilize the same protocol, as described above except the informed consent will not be completed. After completion of the 2^nd^ re-assessment, schedule all participants for a 3^rd^ and final reassessment 28-36 weeks after the baseline assessment. All participants will be asked to continue to monitor their fall frequency, using the fall diary between the 2^nd^ and 3^rd^ study visit.

The 3^rd^ re-assessment will utilize the same protocol, as described above except the informed consent will not be completed. After completion of the 3^rd^ study visit (28-36 weeks after the baseline assessment), participants will be asked to continue to monitor their falls using the fall diary system for a final 12 weeks.
