# Supplementary material for: Validation of an individualized reduction of falls intervention program among wheelchair and scooter users with multiple sclerosis
Source: Medicine (Baltimore). 2019 May 13;98(19):e15418. doi: 10.1097/MD.0000000000015418 (PMC6531239; doi:10.1097/MD.0000000000015418)
Supplement: Supplemental Digital Content [file medi-98-e15418-s002.pdf]

Individualized Reduction of Falls (iROLL): A Multifaceted Fall Prevention Program for Wheelchair and Scooter Users Living with Multiple Sclerosis

Participant Manual

Funding Source: National Multiple Sclerosis Society
(RG-1701-26862)

Principal Investigator:
Laura A. Rice, PhD, MPT, ATP
Assistant Professor
Department of Kinesiology and Community Health
University of Illinois at Urbana-Champaign

Co-Investigators:
Deborah Backus, PT, PhD, FACRM
Director of Multiple Sclerosis Research
Shepherd Center, Atlanta, GA

Elizabeth W. Peterson, PhD, OTR/L, FAOTA
Clinical Professor & Director of Professional Education
Department of Occupational Therapy
University of Illinois at Chicago

Jacob J. Sosnoff, PhD
Professor
Department of Kinesiology and Community Health
Center on Health Aging and Disability
University of Illinois at Urbana-Champaign

Acknowledgement: Special thanks to JongHun Sung, MS, ATC, CES and Toni Van Denend, OTR/L for their assistance in the development of the education materials.

Thank you!

During the development of the iROLL program, our research team has received permission from the below organizations to use and reference their materials in our education program. Throughout the materials, the contributions of these organizations are noted.

Dalhousie University, Wheelchair Skills Program

MossRehab Outpatient SCI Therapy and Wheelchair Clinic

National Multiple Sclerosis Society

Permobil, AB

University of Pittsburgh, Model Center on Spinal Cord Injury, Wheelchair Maintenance Training Program

University of Washington, The Empowerment Project, Northwest Regional Spinal Cord Injury System

Rebuilding Together

Action Planning materials were adapted from ÓSelf-Management Resource Center, LLC*, 1980 - 2017*. All rights reserved. All or portions of this material includec opyrighted materials belonging to Self-Management Resource Center. To obtain a license please contact the Self-Management Resource Center." All Program materials must display the following subtitle "A Self-Management Resource Center Program (SMRC) developed by Dr. Kate Lorig, Virginia González and Diana Laurent."

Welcome!

Dear Participants,

Thank you so very much for your participation in the iROLL fall prevention education program! We are very excited to work with you!

This research study is funded by the National Multiple Sclerosis Society and is designed to evaluate the influence of a structured fall education program. Our hope is to use the findings from this research to develop high-quality education programs that can be used across the United States to help other individuals with Multiple Sclerosis prevent and manage falls.

Our research team has performed a preliminary (pilot) study to examine the influence of a similar fall prevention education program[1]. Results from this study found that after participants received the education, fall frequency decreased and transfer quality and seated postural control (balance) improved. Using the results from this study and feedback from the study participants, we have developed a more in-depth program that we are excited to share with you.

Thank you for your participation! Your involvement in this study may help improve the care provided to other wheelchair and scooter users with Multiple Sclerosis!

If you have any questions during the course of the study, you can talk with your trainer or reach out to Dr. Rice using the contact information below.

Laura A. Rice, PhD, MPT, ATP
219 Freer Hall
906 S. Goodwin Ave.
Urbana, IL 61801
217-333-4650
ricela@illinois.edu

If you would like to view any of the items in this manual online or view any of the videos, you can access them at: https://irolluiuc.wixsite.com/website
Thank you for your participation and we look forward to working with you!

Sincerely,

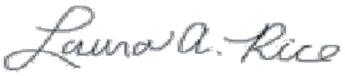

Laura A. Rice, PhD, MPT, ATP

1. Rice LA, Issacs Z, Ousley C, Sosnoff J. Preliminary Investigation of an Intervention to Manage Fall Risk and Enhance Functional Mobility Among Wheeled Mobility Device Users with Multiple Sclerosis. *International Journal of MS Care.* 2017;In Press.

Ground Rules

Below are the ground rules that will be followed during the iROLL program. Please look over these rules before the start of each session. Please feel free to suggest additional rules for the group to discuss.

1. Participants are free to ask questions at any time.
2. When another person is speaking, please be respectful of what the person has to say and do not interrupt.
3. All information disclosed by other participants should be kept confidential.
4. Please provide constructive criticism and highlight positives.
5. Additional ground rules:

GET WISE Framework

The iROLL education materials have been organized using the acronym 'GET WISE.' During the course of the program, we will be referring to this acronym to help orient you to the materials we are discussing. Below is a listing of the various components of GET WISE:

G-Goals
E-Exercise
T-Transfer Training

W-Wheelchair/scooter management
I-Individualized activity in the home & community
S-Symptom Management
E-Environmental safety

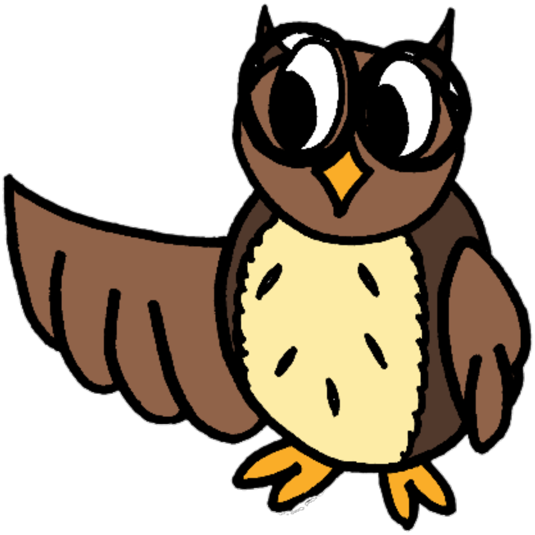

Falls 101- Introduction to falls

GET WISE Section: Individualized Activity in the Home and Community

Introduction:

Falling can have a large impact on the health and well-being of individuals with MS and a negative influence on community participation.

Recently, our research team surveyed full time wheelchair/scooter users living with MS[1] and found that 75% of the population reported at least one fall in a period of six-months. Of those individuals who reported a fall, almost 50% were injured. We also found that over 75% of participants had a fear of falling, and 65% limited the activities they did in their home and community due to this fear.

Due to the high frequency of falls experienced by individuals with MS and the negative consequences, it is very important that steps are taken to prevent falls from occurring! Today you will learn how we define a "fall", how falls most frequently occur, and how fear of falling can also have negative consequences.

What is a fall?

According the World Health Organization, a fall is defined as:

"An event which results in a person coming to rest inadvertently on the ground or floor or other lower level[2]"

Why is it important to prevent and manage falls?

- Falls can result in significant physical injuries (e.g. Fractures, concussion).
- A fear of falling can negatively influence quality of life, community participation, and performance of essential activities of daily living.
- Most wheelchair/scooter users living with MS need assistance to get up after a fall occurs. Individuals who need assistance to recover (get up) are at higher risk for additional physical injuries, a decline in performance of daily activities, an increased chance of a hospital admission, and an increased fear of falling.

How do falls occur?

Research has found falls commonly occur while people are doing the activities (actions) listed below in the locations listed below[1,3]. Please carefully take a look at the lists below and circle the actions that you were doing and locations where you were during a fall. Feel free to write in additional actions you were doing when you fell and locations where you fell.

Common actions being performed during falls

- Wheelchair Transfer
- Wheelchair propulsion (manual wheelchair)
- Driving wheelchair (power wheelchair)
- Reaching for an object
- Walking short distances
- ________________________________
- ________________________________

Common locations of falls

- Bathroom
- Street
- Bedroom
- Living room
- Garage
- Garden
- Kitchen
- Bus
- ______________________________
- ______________________________

Previous research has also found that other factors can contribute to falls[3]. Below is a list of other factors associated with falls among wheelchair and scooter users. It is important to note that most of these factors can be improved with education! Please take a look at the lists below and circle any of the factors that you think might have been associated with a fall you have experienced. Feel free to write in any additional factors.

The characteristics of wheelchair user

- Poor transfer skills
- Poor wheelchair skills
- Poor seated balance
- Muscle weakness and spasticity
- Intoxication
- Distraction/confusion
- Poor depth perception
- _______________________
- _______________________

Environmental hazards

- Wet floor
- Poor lighting
- Unexpected obstacles
- Crack or debris on the street
- Unfamiliar environment
- Uneven surface
- Inaccessible environment
- ____________________________
- ____________________________

Equipment (wheelchair)

- Footplate Design
- Forgetting to engage wheel locks or turning off chair
- __________________________
- __________________________

Point of discussion: *What actions, locations and factors did you circle or write down?*

It is also important to note that most falls are caused by many risk factors working together[1]. Very rarely does one single event cause a fall. For example, falls frequently occur in the bathroom while a person is transferring to the toilet or the shower. A wet floor is often involved. Another common association is a fall occurring while a person is pushing or driving the wheelchair/scooter on the sidewalk and hits a crack or piece of debris. Because there are so many risk factors for falls and they often occur together, this education program covers a wide variety of risk factors that can be modified.

Being aware of the risk factors for falls will help you to avoid dangerous situations. During this program, we will help you to learn how to manage these risk factors.

Fear of Falling

Another important aspect of prevention is managing fear of falling. While most of our understanding of fear of falling comes from research involving older adults, there is also research about fear of falling based on people with MS and full-time wheelchair/scooter users with MS.

Although limited, evidence points to two important facts about fear of falling among people with MS:

1) It is very common [4]
2) It can increase fall risk [5]

Point of discussion: *Why do you think fear of falling increases fall risk?*

Take a look at Figure 1 on page 9. Research involving older adults and people with MS tells us that a fall can lead to fear of falling OR fear of falling can lead to a fall [5-7].

Figure 1 also shows us that the cycle can be self-perpetuating, in other words a fall or fear of falling can turn into a bad cycle of having more fear, cutting back on activity, and having more falls.

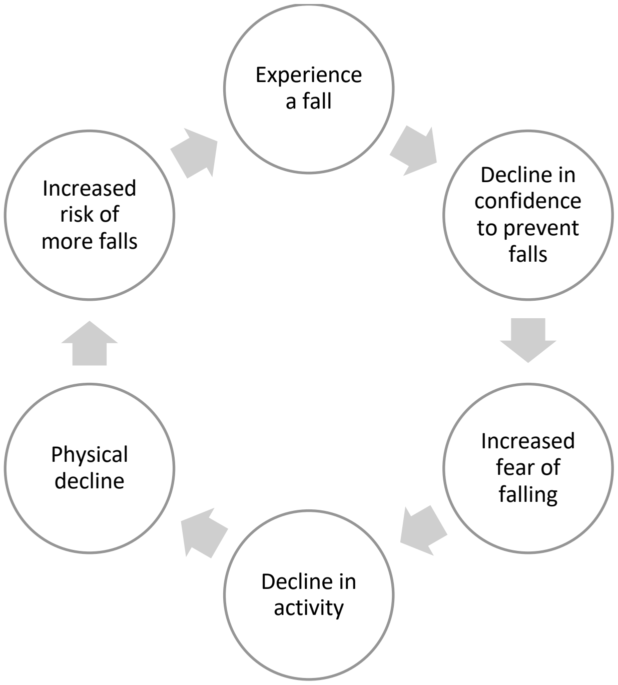

Figure 1 - Fear of Falling

The issue here is not only that fear of falling increases fall risk, but it can impact your quality of life. Fear of falling can lead you to give up activities that are fun or important to you.

We know that fear of falling is a big concern among wheelchair/scooter users, even if they haven't had a bad fall[1]. That probably does not surprise you!

Fear of falling is a rational response to a real concern for people with MS: the fact is, there are symptoms of MS that can lead to a higher risk of falling.

The key is to think about your own activity to determine if your concern about falling is keeping you from doing things that really are not safe for you to do or keeping you from doing things you **safely could be doing**. In other words, there is a difference between a fear of falling that is **protective** and a fear of falling that is greater than it needs to be, given your abilities [8]. The latter often leads to an **unnecessary** decline in activity.

Let's take a look at two examples: Jerry and Pam.

Jerry is 47 years old, diagnosed with secondary progressive MS. Three years ago, he began to have more trouble with his walking. He decided to start using a 4-wheeled walker around the home and a manual wheelchair for longer distances. He found the walker and the wheelchair at a yard sale.
About a month ago, Jerry had a fall. He was getting the mail, just as he usually does one Monday afternoon. He rolled his wheelchair to the mailbox, stood up to get the mail inside

the mailbox, and when he went to sit back down after retrieving the mail, he fell to the ground.
Ever since that fall, Jerry just cannot shake this fear of falling. He's decided it's best to no longer get the mail. He is also thinking he should cut out going on the weekly shopping trip with his wife.
Jerry sees himself as a self-reliant person. Rather than talk about his experience and fears with his family and/or doctor, he's decided to try to sort things out on his own and cut back on activities as he sees fit.

Pam is 53 years old, diagnosed with primary progressive MS. Early on in her MS journey, she began to have trouble walking due to weakness, fatigue and balance issues.
Pam and her doctor developed a plan for Pam to use a cane initially, which then transitioned to occasional walker usage.
The difficulty with walking continued, and Pam and her doctor recently decided using a manual wheelchair for daily mobility is a safe and appropriate choice. They are also starting to talk about a power chair for longer distances.
Last month, Pam fell while transferring from her wheelchair to the toilet. This fall really shook Pam up. She found herself worrying a week after the fall, and then two weeks after the fall. She was also mad because she knew she should be able to get on and off the toilet safely without worrying about it all the time.
Pam felt like her fear of falling of a low-grade hum that got louder when she was in the bathroom. Like Jerry, Pam felt strongly that she could take care of herself. Unlike Jerry, Pam believed that taking care of herself included building a team of supports as needed. In line with this view of self-sufficiency, Pam decided to talk to her doctor about her fear of falling. Her doctor listened carefully and referred her to outpatient physical therapy (PT) and occupational therapy (OT).
During her PT sessions, Pam worked on strengthening her leg muscles, improving balance for stand-pivot transfers, and safety during transfers. She also got a home program and now is dedicated to her exercise routine.
During her OT sessions, Pam learned more about safety in the bathroom in general and available tools to prevent falls, such as how to use a toilet safety frame, bath bench, and hand-held shower safely. She also learned how to better manage her fatigue and how to better assess the match between her physical abilities at a given time and the demands of an activity.
She discovered some her own habits might be adding to her fall risk. For example, Pam was wearing socks while transferring, and letting her dog, Cutie, into the bathroom while she was in there.
Breaking these habits and building safer approaches to activities in the bathroom helped Pam feel much more confident in her ability to prevent future falls. She has continued to transfer well, has some falls prevention strategies "in her pocket", and has a new awareness of her surroundings and daily symptoms.

Take home points:
Jerry and Pam both had a fall. They both experienced challenges with their daily mobility. They both, in fact, experienced some fear of falling, which was understandable. The difference is **that they choose different paths to deal with their concerns**. Jerry chose to **cut back on his activities and not communicate with those around him**. Pam choose to **talk to her doctor**. With her health care team, she came up with some strategies to **improve her overall strength, confidence with transfers and prevent future falls.**
A response like Jerry's can lead to a **physical decline, more fear of falling, and an increased fall risk.** You can take action to avoid having a story like Jerry's. Pam's happy ending started by **being aware** that she was experiencing concerns about an activity she should be able to do safely and talking to her doctor about it.

Point of Discussion:
Work in pairs to discuss the following:

1) Name one activity that raises your concerns about falling, even though you think you should be able to do it safely, without falling.

2) Name one thing you have done to address those worries OR one thing you could do.

References

1. Rice L, Kalron A, Berkowitz SH, Backus D, Sosnoff JJ. Fall prevalence in people with multiple sclerosis who use wheelchairs and scooters. *Medicine.* 2017;96(35):e7860.
2. Al-Faisal W, Beattie L, Fu H, et al. *WHO Global Report on Falls Prevention in Older Age.* 2007.
3. Sung J, Trace Y, Peterson EW, Sosnoff JJ, Rice LA. Falls among full-time wheelchair users with spinal cord injury and multiple sclerosis: a comparison of characteristics of fallers and circumstances of falls. *Disability and rehabilitation.* 2017:1-7.
4. Peterson EW, Cho CC, Finlayson ML. Fear of falling and associated activity curtailment among middle aged and older adults with multiple sclerosis. *Multiple sclerosis.* 2007;13(9):1168-1175.
5. Mazumder R, Lambert WE, Nguyen T, Bourdette DN, Cameron MH. Fear of Falling Is Associated with Recurrent Falls in People with Multiple Sclerosis: A Longitudinal Cohort Study. *International journal of MS care.* 2015;17(4):164-170.
6. Boswell-Ruys CL, Harvey LA, Delbaere K, Lord SR. A Falls Concern Scale for people with spinal cord injury (SCI-FCS). *Spinal cord.* 2010;48(9):704-709.
7. Friedman SM, Munoz B, West SK, Rubin GS, Fried LP. Falls and fear of falling: which comes first? A longitudinal prediction model suggests strategies for primary and secondary prevention. *Journal of the American Geriatrics Society.* 2002;50(8):1329-1335.
8. Delbaere K, Crombez G, Vanderstraeten G, Willems T, Cambier D. Fear-related avoidance of activities, falls and physical frailty. A prospective community-based cohort study. *Age and ageing.* 2004;33(4):368-373.

Session #1 Journal Entry

GET WISE Section: Goals

What will be done:

You will be asked to make a journal entry every week after completion of the session. Your journal entry will be tied to items discussed during the previous session. You will typically be asked to respond to a specific question regarding the content of the program from the previous session.

Why:

The journal entries will help you to think about the information that was presented during the education session and help you integrate what you have learned into your everyday life.

Prompt:

Please reflect on your most recent fall. Think about what you were doing when you fell, where you were, and if any other factors influenced your fall (i.e. slippery surfaces, poor lighting, etc.) Based on the information you learned today, how do you think you can decrease the frequency of your falls?

Response:

<u>Exercise Program Description</u>

GET WISE Section: Exercise

Exercise is a very important component of this education program. During the course of the program, we will discuss the importance of exercise almost every week and will refer back to this program.

Previous research has found that exercise is beneficial in MS. Exercise can:

- increase physical fitness (muscle strength and cardiorespiratory function)[1]
- enhance mobility [2]
- improve balance[3]
- enhance cognitive function [4]
- reduce fatigue[5]
- improve in depressive symptoms [6]

Exercise can also help prevent falls in MS.

- Exercise can help improve sitting balance by strengthening the core (stomach and back) muscles[7]. Good sitting balance can help maintain an upright posture and makes you more stable when reaching for items. Strong core muscles can also help make you more stable when doing transfers.
- Exercise can also strengthen your arm muscles, which will be helpful when transferring and performing wheelchair/scooter skills.

Point of discussion:

Do you exercise regularly?

- If yes, what types of exercises do you do? What helps you to exercise?
- If no, what prevents you from exercising?

During the course of the program, you will be asked to do a series of exercises designed to strengthen your core and arm muscles. These exercises will help improve your balance in a seated position and are used during many functional activities, such as transfers and wheelchair/scooter skills.

A complete listing of the exercises and detailed instructions are provided on page 16 (Exercise manual)

You can also find a video of the exercises on the study website.

Name of video: Exercise program

General notes:

- Most of these exercises are performed in a sitting position.
- Please sit on a firm, stable surface and place your hands on either side of your legs.
- You can use your hands to assist with balance to start with, but as you get stronger, try to put your hands in your lap during the exercises.
- Ideally, your back should not be supported.

Have a friend or family member stand next to you to assure that you don't fall during the exercises.

Most importantly**: Listen to your body!**

After you exercise you might feel soreness in your muscles but not pain or extreme fatigue. Soreness that goes away after about 24 hours is okay. This means that you worked the muscle enough to gain some benefits but not enough to cause damage. As you do the program more frequently, the soreness that you feel should reduce.

If you are feeling extreme fatigue or soreness, decrease the number of repetitions and/or reduce the number of days per week that you exercise.

If you do not feel that the exercises are difficult, increase the number of repetitions performed (increase by five repetitions each week, with a max of 20 repetitions each, five days per week).

You can discuss your program with your trainer during the course of the program.

References:

1. Platta ME, Ensari I, Motl RW, Pilutti LA. Effect of Exercise Training on Fitness in Multiple Sclerosis: A Meta-Analysis. *Archives of physical medicine and rehabilitation.* 2016;97(9):1564-1572.
2. Sosnoff JJ, Sung J. Reducing falls and improving mobility in multiple sclerosis. *Expert review of neurotherapeutics.* 2015;15(6):655-666.
3. Paltamaa J, Sjogren T, Peurala SH, Heinonen A. Effects of physiotherapy interventions on balance in multiple sclerosis: a systematic review and meta-analysis of randomized controlled trials. *Journal of rehabilitation medicine.* 2012;44(10):811-823.
4. Sandroff BM, Pilutti LA, Benedict RH, Motl RW. Association between physical fitness and cognitive function in multiple sclerosis: does disability status matter? *Neurorehabilitation and neural repair.* 2015;29(3):214-223.
5. Heine M, van de Port I, Rietberg MB, van Wegen EE, Kwakkel G. Exercise therapy for fatigue in multiple sclerosis. *The Cochrane database of systematic reviews.* 2015(9):Cd009956.
6. Ensari I, Motl RW, Pilutti LA. Exercise training improves depressive symptoms in people with multiple sclerosis: results of a meta-analysis. *Journal of psychosomatic research.* 2014;76(6):465-471.
7. Rice LA, Isaacs Z, Ousley C, Sosnoff J. Investigation of the Feasibility of an Intervention to Manage Fall Risk in Wheeled Mobility Device Users with Multiple Sclerosis. *Int J MS Care.* 2018;20(3):121-128.

<u>Exercise Manual</u>
GET WISE Section: Exercise

<u>Warm-Up:</u> Before you start your exercise routine, be sure to perform a short warm-up to get your muscles ready to go.

Round and Arch Spine*:* Round your shoulders forward and then arch your back. Each time you arch your back counts as one repetition. Start with a small movement and then try to go through a greater range of motion as your muscles warm up.

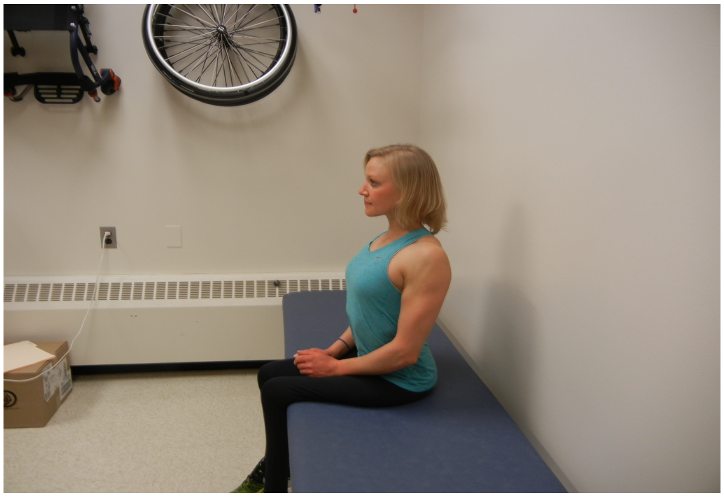

Start

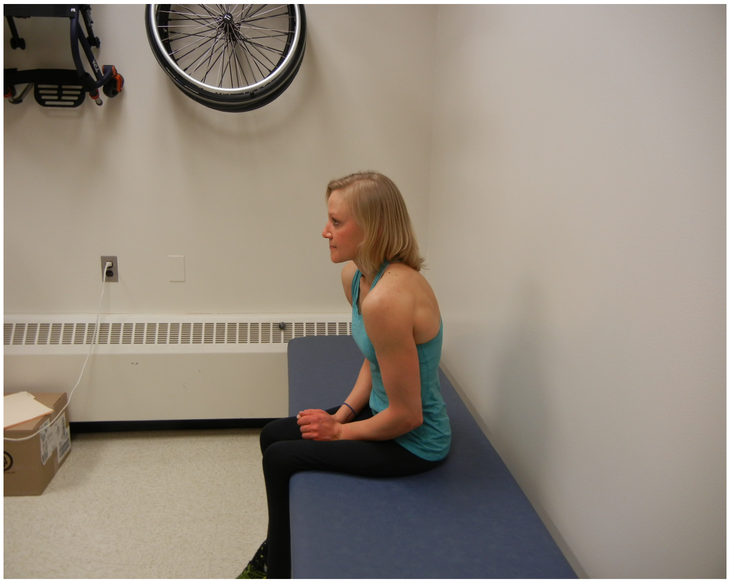

Finish

1) Lateral Spinal Flexion: While you are sitting, bend your body to the right side, pause, then to the left. Each time you bend to the left counts as one repetition.

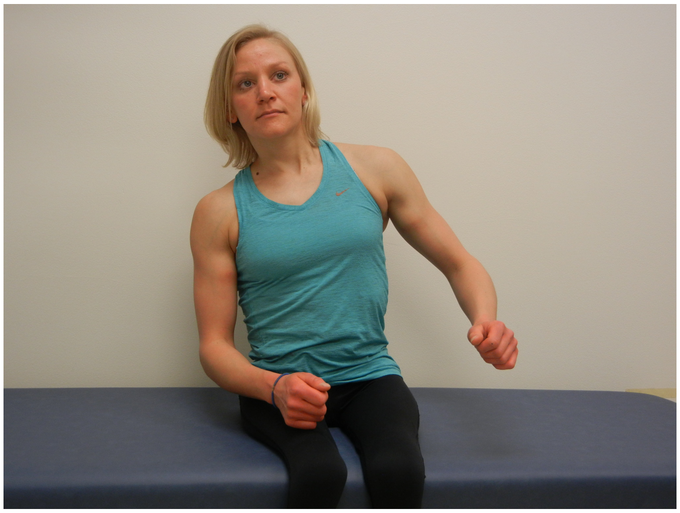

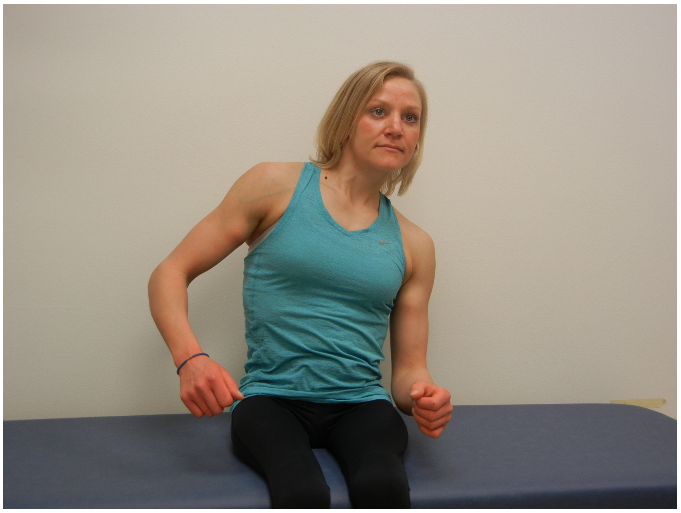

Start **Finish**

Challenge: Lift your arms over your head while performing the exercise.

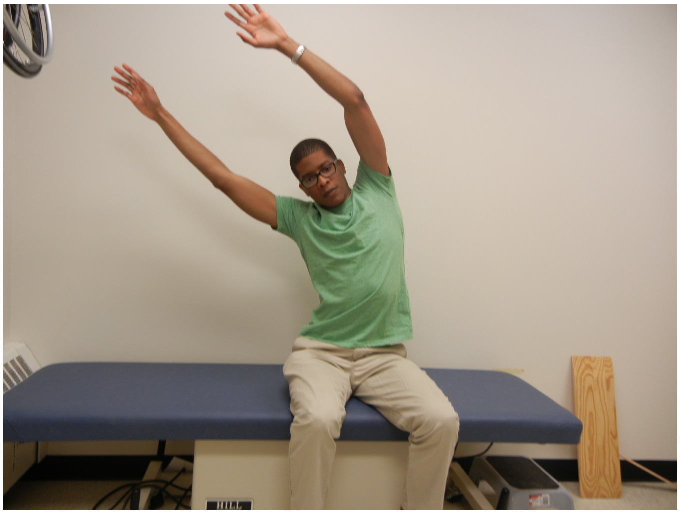

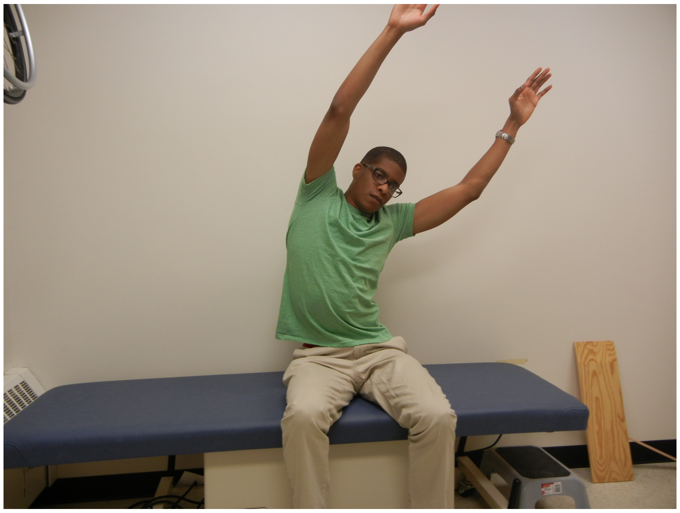

Start **Finish**

2) Lean Backs*:* Lean your body as far back as possible. Hold this position for approximately five seconds, and then return to an upright seated position. Try not to use your hands to support your body.

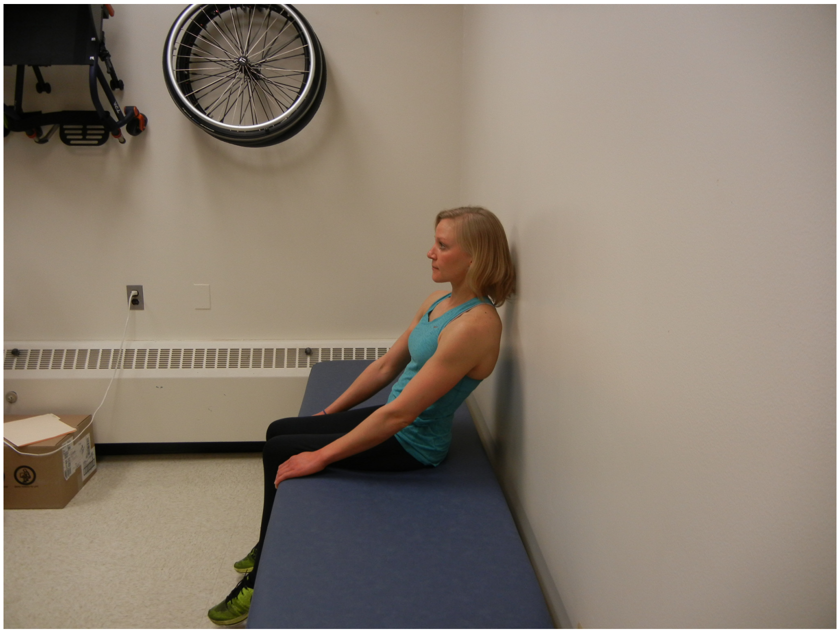

3) Scapular Retraction*:* Keeping your back straight, bend your elbow to 90 degrees; lift your arms approximately six inches away from the sides of your body. Squeeze your shoulder blades together and hold for approximately five seconds each time.

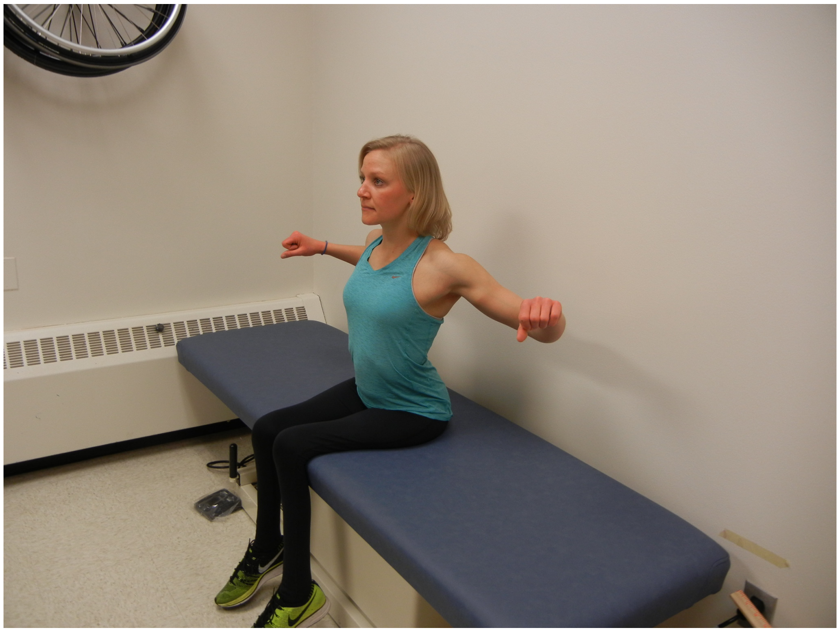

4) Scapular protraction*:* Lie on your back while holding a hand weight. If you don't have weights, you can use water bottles. Keeping your elbow straight, push your hand up towards the ceiling, hold, and return to starting position. Perform this exercise one arm at a time.

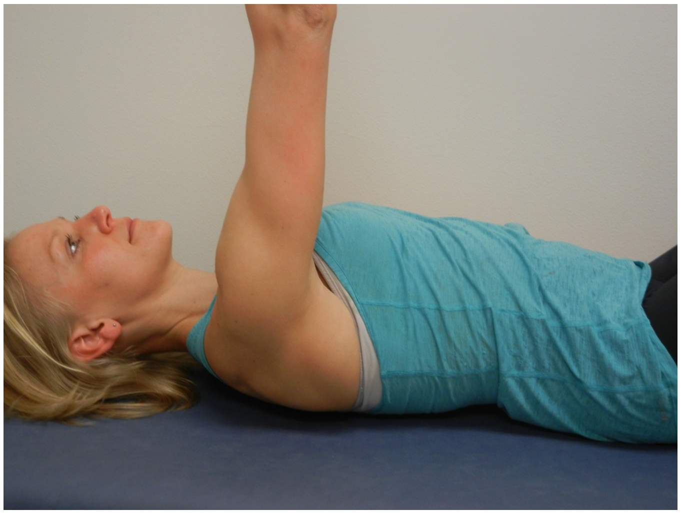

If you are uncomfortable on your back, you can also perform this exercise in a seated position.

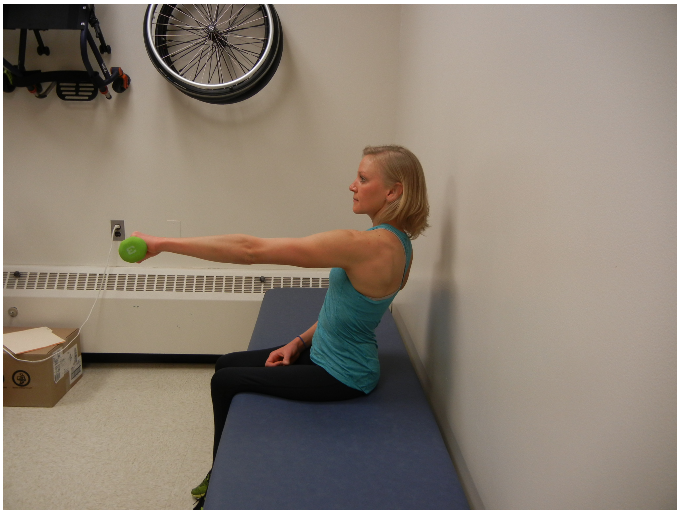

5) Forward/Lateral Reach: Reach forward to the right, and to the left. Each time you reach forward counts as one repetition.

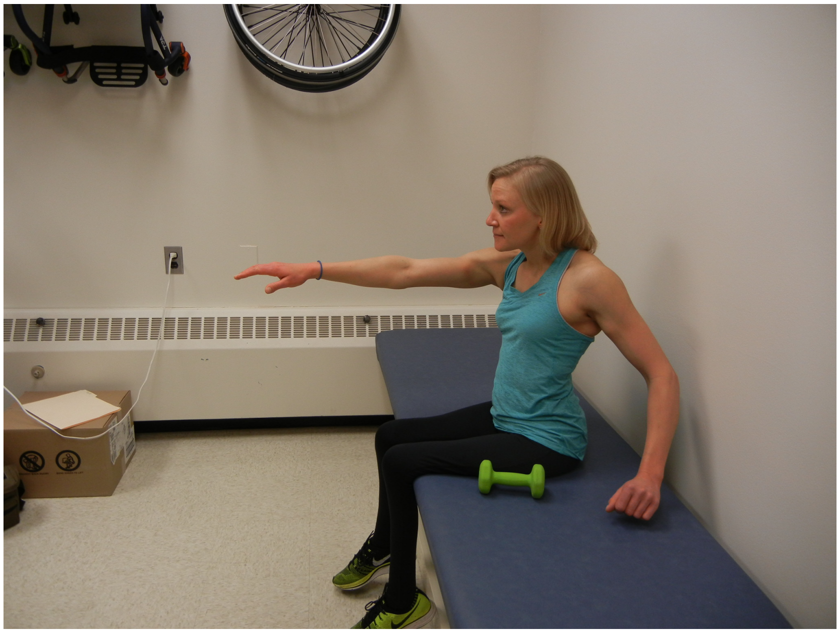

Reach Forward

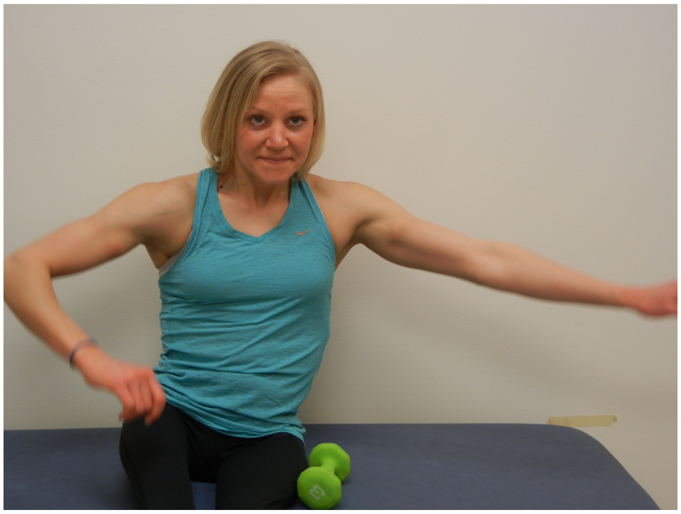

Reach Left

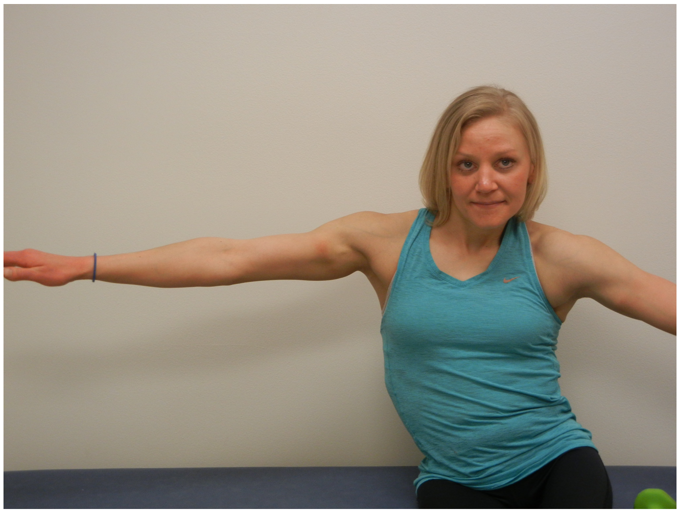

Reach Right

6) Scooting: Use your abdominal and hip muscles to scoot your bottom to the right two inches, backwards two inches, to the left two inches, and forward two inches. Please, try not to use your hands unless absolutely necessary.
You will end in the same position you started. Each time you scoot forward counts as one repetition.

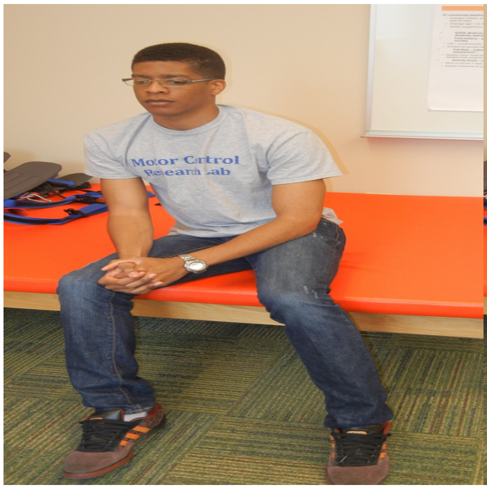

Scoot Right

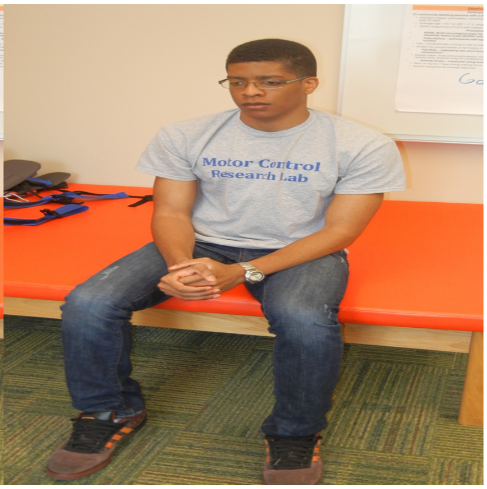

Scoot Backward

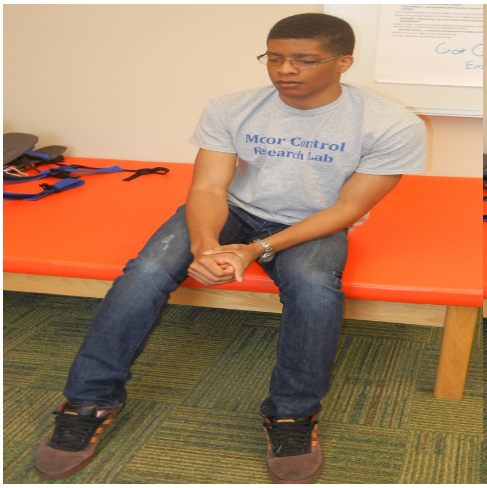

Scoot Left

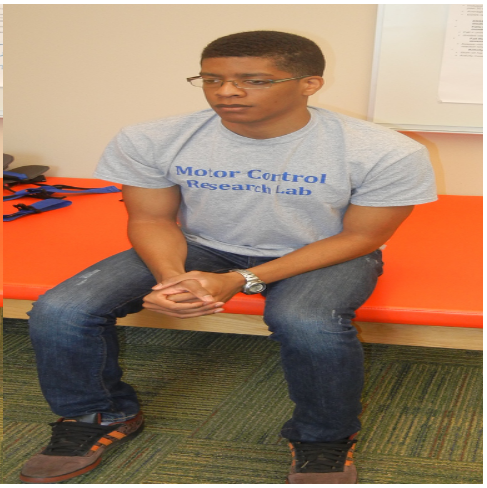

Scoot Forward

***7) Press up**:* With your hands on a firm surface, push yourself up using your arms and shoulders and hold for a few seconds. Slowly lower your body back into the seated position.

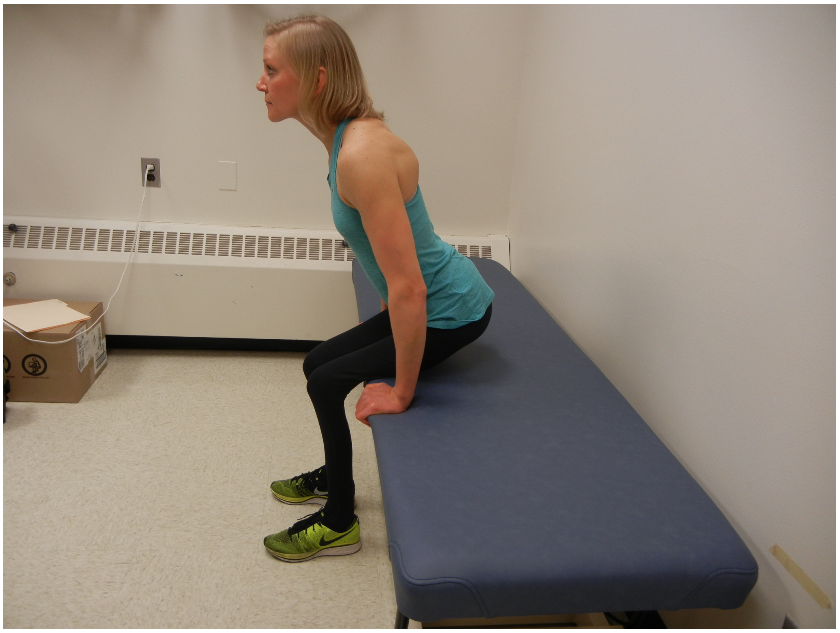

8) Shoulder press**:** Bend your elbows and raise your arms to a 90 degree position. Grasp the weights so your palms are facing forward with your hands slightly wider than your shoulders. Slowly straighten your elbows and raise the weights above you. Then, slowly lower the weights back down to starting position.

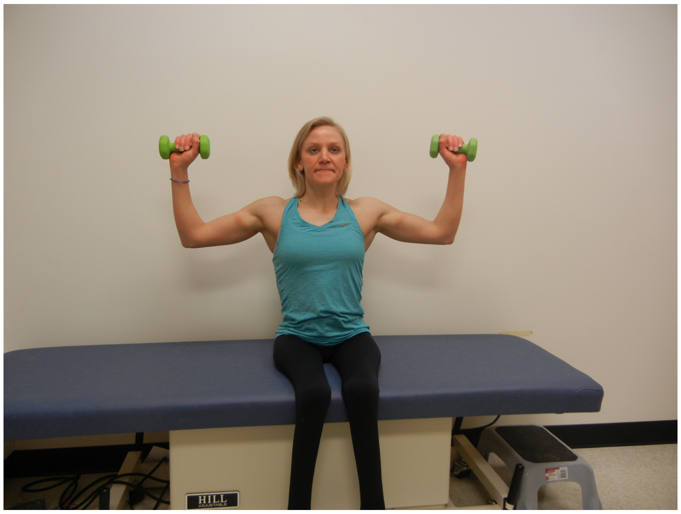
Start

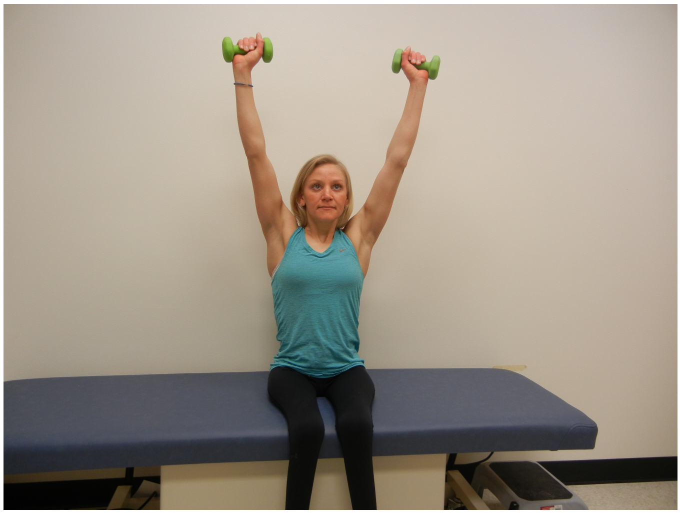
Finish

If you have trouble raising both arms at the same time, you can modify this exercise by raising only one arm at a time.

Start

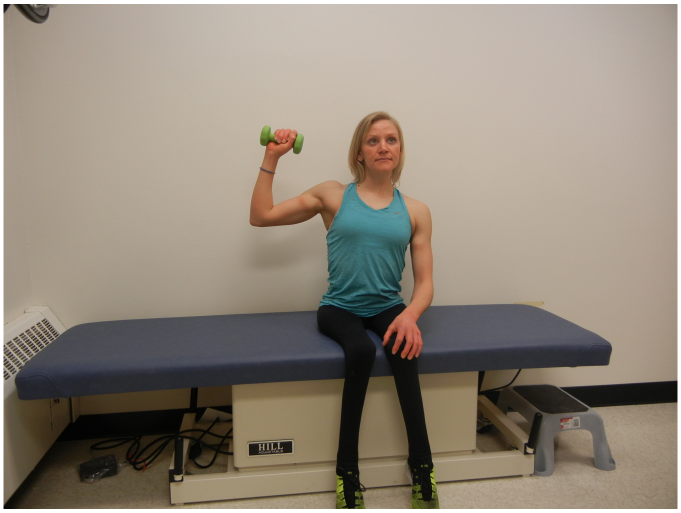

Finish

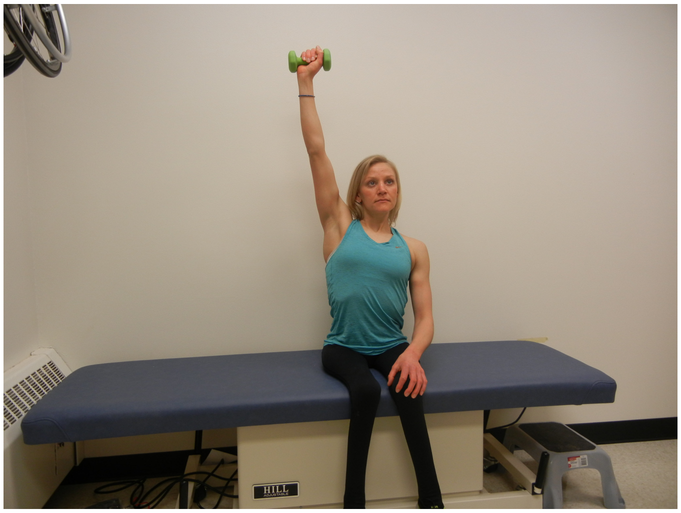

9) Shoulder Flexion and Abduction: Raise your arms in front of you at shoulder level and slowly lower your arms back down to starting position. Then, raise your arms out from your sides to shoulder level and lower your arms.

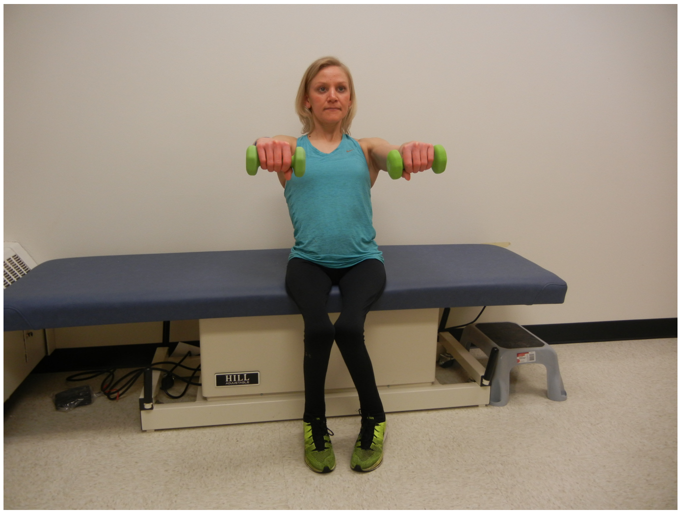
Flexion

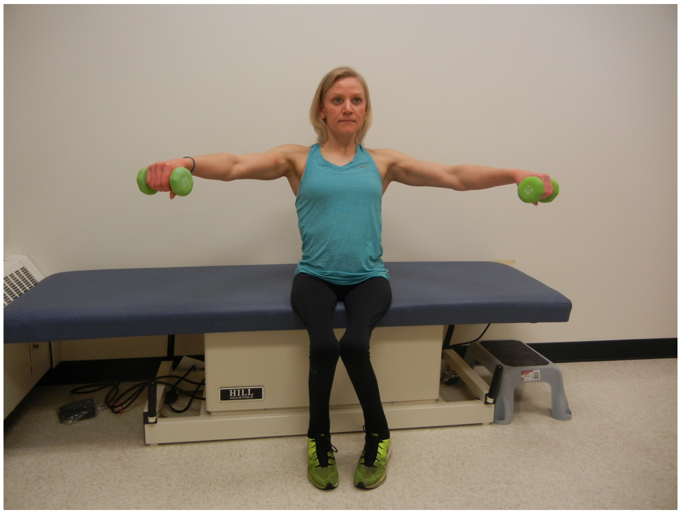
Abduction

This exercise can also be done using one arm at a time.

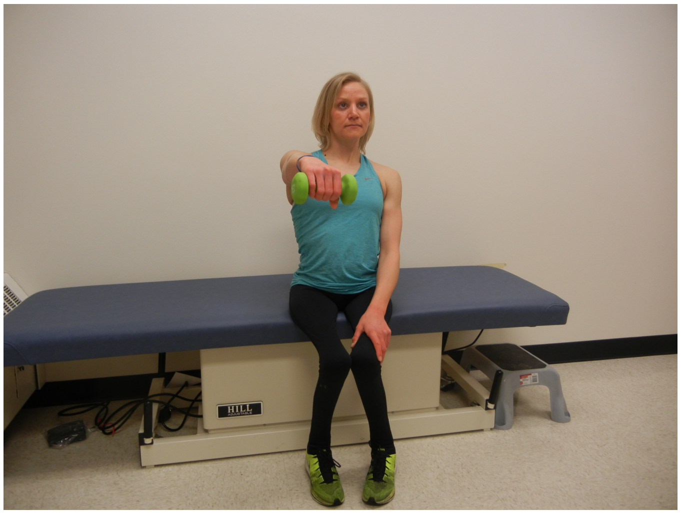
Flexion

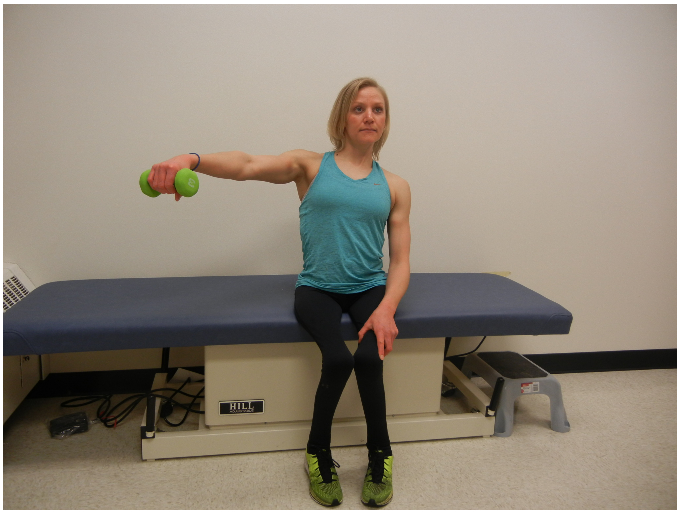
Abduction

<u>Cool Down</u>: To help you recover faster, finish up your exercise routine with a few stretches.

Rotational twist (lower back stretch): Gently twist your upper body to the right and hold for 20 to 30 seconds, and twist to the left and hold for 20 to 30 seconds.

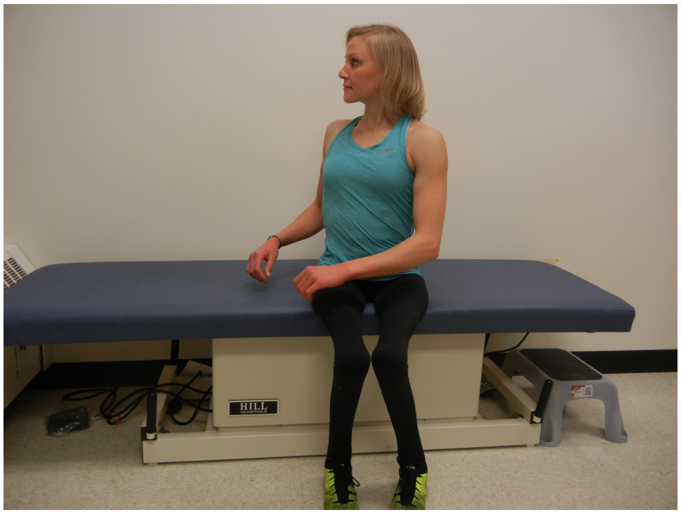

Right

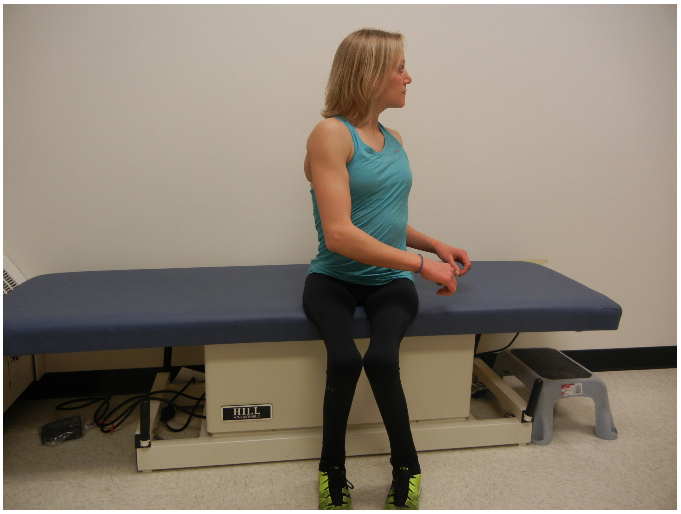

Left

***Side stretch*:** Gently lean to your right side and hold that position for 20 to 30 seconds. Repeat on left side.

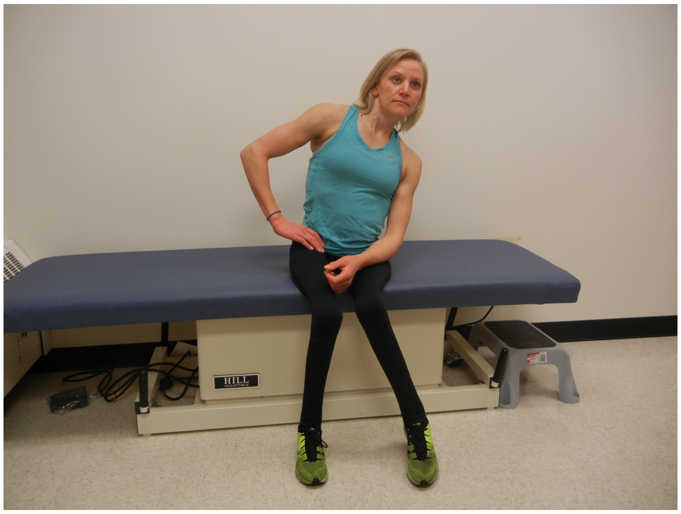

Right

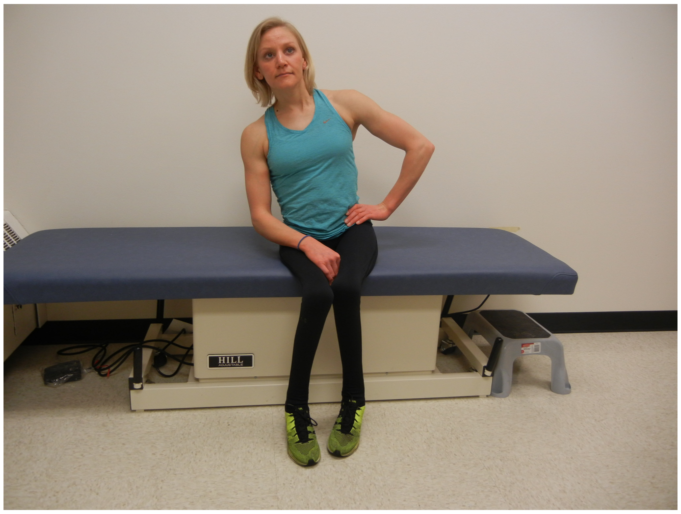

Left

You are done!

SMART Goals Description

GET WISE Section: Goals

During the course of this education program, we will be setting goals at the end of each session. Just like the journal, setting goals will help you make the most of what you are learning and help you to work towards doing things that are important to you. As much as possible, we will be using the SMART goal format listed below to help make the goals as useful as possible. Please look over each component of a SMART goal below. On the next page, we will use this format to set an actual goal. You can refer back to this page during the course of the education program to help develop your goals.

S	Specific.<br>This should be very specific to what you want to do. Example: *I want to engage in an exercise program to improve my abdominal and upper extremity strength.*
M	Measurable.<br>Attaching a measurement to the goal will help you track your progress and stay focused. Example: *I will do my exercise program 3 times per week.*
A	Achievable.<br>You should be able to achieve your goal in a reasonable time frame. Using the exercise example, think about how confident you are that you can do the program a certain number of times per week. Pick a number that you feel fairly confident that you can do.
R	Relevant.<br>The goal you set should be meaningful to you and something that you really want to do! Think about if the goal if worth your time and effort. If you don't feel it is, you will be less likely to achieve the goal.
T	Time bound.<br>Set a time limit on the goal. Example: *I will do my abdominal and upper extremity exercise program 3 days a week for the next 3 months.*

Session #1 Goal Sheet

During Session #1, the home exercise program discussed was designed to target core and arm muscles.

Please start doing each **exercise 10 times, three days per week.** Getting into a routine is very important in order to maintain an exercise program in the long term. It is also very important to pick specific days/times and locations that you think you will be able to complete the exercise program.

What days of the week do you think you will do your exercises?

Please circle three days that you think you will be able to do the exercise program. You can exercise more than three days, but please try to start exercising *at least* three days per week.

Sunday   Monday   Tuesday   Wednesday Thursday   Friday   Saturday

What time of the day do you think you will complete the exercise program?

You don't have to do your exercises the same time every day but it is often easier to establish a routine. For example, you could plan to do your exercise program Monday, Wednesday and Friday mornings after you get dressed. When thinking about what time of day to do the exercise program, it is best to wait approximately 30 minutes after you eat. Also, you may not want to do the program first thing in the morning as your body may need time to adjust to being awake. You should also plan to finish your exercise program about 1 hour before going to bed so your body has time to relax and unwind before going to sleep. Finally, you may also want to think about times during the day that your MS symptoms are at their worst. Avoid times that you think you might be very tired or have increased spasticity.

Potential times:

Where do you think you will do the exercise program?

These exercises are performed in a sitting or lying position. Please do the exercises on a firm, stable surface. A firm mattress or couch often works well. Ideally, your back should not be supported.

Potential locations:

What equipment or assistance will you need?

The program was developed to use minimal or no equipment. However, to progress the program, you might want to buy some light hand weights or resistance bands. You can buy many of these items on amazon.com or your local athletic store (Dick's Sporting Goods, Finish Line, etc.). When you first start, you should have a friend or family member stand next to you to assure that you don't fall during the exercises.

Equipment to use:

What challenges do you think you might face?

Setting the goal:

Please write out your exercise goal using the SMART goal framework and share your findings with the group:

S	Specific:
M	Measurable:
A	Achievable:
R	Relevant:
T	Time bound:

Make it a Habit!

One of the most difficult parts of exercising is getting started. Once you get started and have establish a routine, it is easier to stick with the plan.

Therefore, once you have decided when, where, and how you will exercise, try to stick with that plan. Even if you are unable to do the full exercise program, please try to do at least one to two reps of each exercise to keep your routine going!

On a scale of 0-10, how confident do you feel that you can do each exercise 10 times, three days per week? Please write your answer below and share this information with the trainer.
_____________/10

Exercise Log:

Please keep track of your exercise frequency using the provided exercise logs. On page 31 of the manual, you will see an example of the exercise log and how it should be completed. In your supplemental packet, you will also find blank exercise logs to be used during the course of the exercise program.

i-ROLL: Exercise log

Please place a check mark on each day you complete the various exercises listed below.

Exercise	Date & Day	Goal for a week	Monday	Wed	Friday			
Warm-up	Round and Arch Spine	20	20	20	20			
Routine	Lateral spinal flexion	10/2	8/2	9/2	10/2	/	/	/
	Lean backs	10/2	10/2	10/2	10/2	/	/	/
	Scapular retraction	10/2	10/2	10/2	10/2	/	/	/
	Scapular protraction	10/2	10/2	10/2	10/2	/	/	/
	Forward/lateral Reach	10/2	11/2	10/2	10/2	/	/	/
	Scooting	10/2	4/2	4/2	5/2	/	/	/
	Press up	10/2	11/2	10/2	10/2	/	/	/
	Shoulder Press	10/2	10/2	9/2	10/2	/	/	/
	Shoulder flexion and Abduction	10/2	10/2	10/2	10/2	/	/	/
Cool down	Rotational twist (20-30 seconds)	2	2	2	2			
	Side Stretch (20-30 seconds)	2	2	2	2			

Note:

Session #2 To Do's

Please complete the following items in the week between Session #1 and Session #2.

- Look through your program manual to familiarize yourself with the program.
- Respond to the journal question (Page 12) regarding your most recent fall.
- Look over your exercise goal sheet and make any additional revisions (Page 28).
- Begin doing the home exercise program three times per week (Page 16).
- Use your exercise log to track your exercises. (folder)

Transfers 101

GET WISE Section: Transfers

What is a transfer?

A transfer is a movement from a wheelchair or scooter to/from a desired surface. Transfers are critical for performance of necessary activities of daily living and doing desired activities in your home or community. A transfer can be performed either completely independently or with the assistance of assistive technology and/or a care partner.

Point of discussion:

- How many times do you transfer each day?
- Have you ever fallen during a transfer?
- Are there any transfer situations in which you frequently fall?

Why is learning how to properly perform a transfer important?

- Transfers are used frequently! You need to transfer to perform even very basic activities of daily living, such as getting out of bed in the morning, using the toilet, or getting into a car.
- Falls often occur during transfers, especially when people rush through the activity or transfer on slippery or unstable surfaces.
- In addition to preventing falls, using proper transfer techniques will help prevent the development of upper extremity injuries.

Tips for success:

Please see the information below for some tips to help make your transfers as safe and efficient as possible:

- **Take a time-out before your transfer!** It is easy to get into a routine with your transfers. On one hand, this is a good thing because it makes our movements more efficient, but it is still important to pause before each transfer to make sure everything is set up correctly and pay attention during each step of the transfer. Before you transfer, take a brief **'time-out'** to make note of any potential problems. This step is very important to prevent falls!
- **Set up the transfer!** If you set yourself up to transfer correctly, the rest of the transfer will be easier and go smoothly. Instructions on how to set up the transfer will be provided in the next section.
- **Think about how your body is feeling.** Are you tired or have increased spasticity? If so, ask yourself if you can wait to perform the transfer until you are feeling better or if you can perform the activity in another way.
- **Check the surface your feet are on and where you will be transferring to.** Is it wet or slippery? Are there any obstacles that might get in your way? If possible, try

to change the conditions of the surfaces (dry and stable). If not possible, try to find another place to perform your transfer.

In the next sections we will show you how to properly perform a transfer. We have two different versions of the education materials:

1) **Independent transfer**: This material provides information on how you properly perform a transfer without assistance. If you perform transfers independently, please see page 35.

2) **Assisted transfer**: It is very important to learn how to effectively work with a care partner if you receive assistance during transfers. This material provides information on how to perform a transfer and how care partners can properly and safely provide assistance. If you need someone's help when you perform a transfer, please see page 39.

You can find transfer education videos for both "independent transfer" and "assisted transfer" on the study website. The video will help you visualize the proper transfer techniques.

Please note: A transfer is a complex skill to perform which requires a lot of practice. If you feel you would like to get additional practice and/or feedback on your skills, please talk to your physician about how to continue working on the transfer skills after the education program. He/she may provide a referral to a physical or occupational therapist, who will further help you with the transfer training.

Independent Transfer Education

GET WISE Section: Transfer

Learning how to properly perform a transfer is very important, as transfers are used frequently and are needed to perform even very basic activities of daily living. Learning the proper techniques will help keep you safe, prevent falls, and prevent the development of upper extremity injuries.

A transfer is divided into three phases: the set-up phase, flight phase and end phase. The set-up phase is the longest portion of a transfer and involves the placement of your wheelchair/scooter and your body. Setting up the transfer correctly will make the flight and end phase of the transfer very easy and is vital to preventing falls. The flight phase of the transfer is the portion in which your body is being lifted and moved to the new surface. Finally, the end phase involves safely landing on the target surface and adjusting your body.

Please review the points below about each phase of the transfer.

- **General Tips to Reduce the Frequency of Falls and Injury**

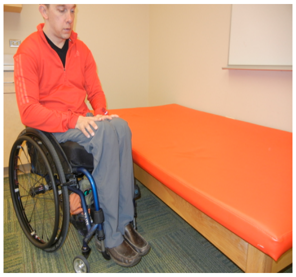

Level Transfer

  - Perform a level transfer whenever possible.
    - Look for alternative surfaces to transfer to (not just the most convenient place), or adjust the height of the surface (if possible). *Example*: Adjust the height of your tub seat or bedside commode so you do not need to perform an uphill transfer every day.
  - Alternate which arm you use to push off with (Example: don't always use your right arm as your push off arm – alternate between left and right.)
  - Use a transfer board or other assistive technology if you have difficulty performing transfers.
  - Try to make the transfer as easy as possible.

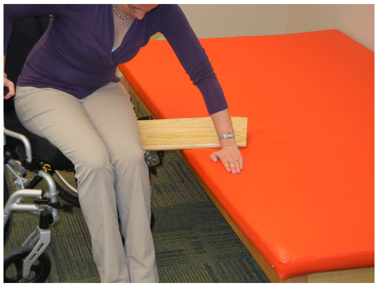

Transfer Board

SET UP PHASE: Position your chair and body properly for a safe and easy transfer.

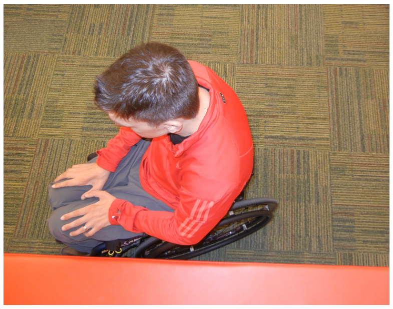

Proper Chair Position

- **Step 1: Position Your Chair**
  - Your chair should be as close to the surface you are transferring to as possible (Ideally, the chair should be touching the surface).
    - Shorter distances decrease the potential for falls.
    - Shorter distances decrease the amount of time you have a significant amount of weight on your arms.

- Position your chair with a 20-45 degree angle between your chair and the surface.
  - This position will help put your shoulder in the best alignment for the correct transfer position.
- Put on your **wheelchair/scooter brakes** when you are happy with your position.

- **Step 2: Chair Set Up**
  - If you have an armrest or sideguards on your chair, take them out of the way before you transfer (if possible).

- **Step 3: Feet and Body Positioning**
  - Place your feet in the most stable position (on the floor if possible) before you transfer. Even if you have significant weakness in your legs, putting them in the proper alignment will provide a small amount of support and stability.
  - Sit on the front 2/3 of your chair (or close to the edge of the surface you are transferring from).

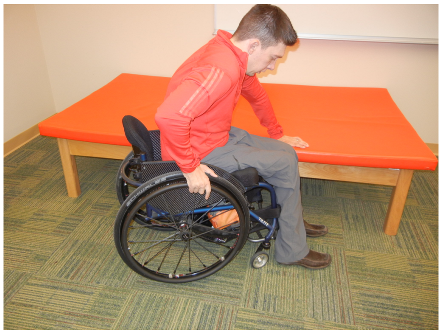

Feet and Body Position

- **Step 4: Hand and Shoulder Positioning**
  - Place your hands in a stable position prior to the start of the transfer.
    - The hand that you will be pushing from is close to your body and holding onto a stable object.
    - Your other hand should be positioned close to where you intend to land. The angle between your arm and body should be approximately 45 degrees.
  - Use a handgrip, if possible.
    - You can use the armrest on your chair, the edge of a mat table/bed, etc.
    - If no handgrip is available, place your hand flat on the surface you are transferring from.

→ Don't reach for a handgrip in a position that will make you unstable!

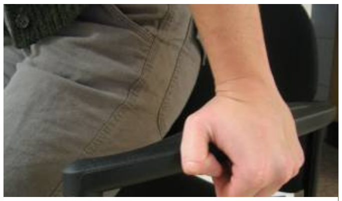

Best!

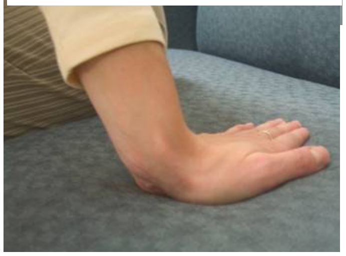

Try to Avoid

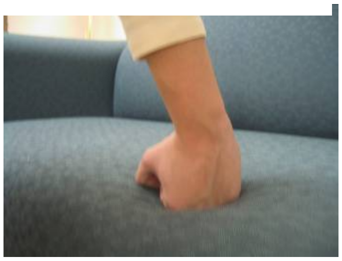

Try to Avoid

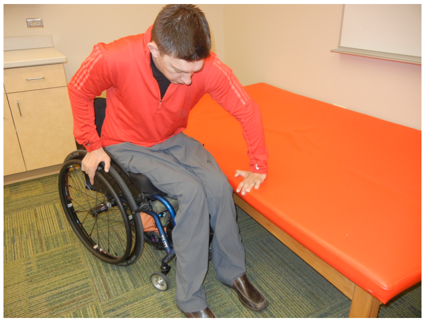

Proper Hand and Shoulder Position

FLIGHT PHASE: Movement from point A to point B. If you have taken time to set up the transfer properly, this will be easy.

- Use the "head-hips" relationship.
    - Move your head in the opposite direction that you want your hips to move.This makes the transfer easier and reduces the potential for falls.
- The flight should feel very smooth and well-controlled.

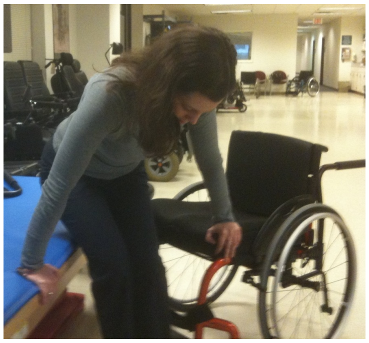
Head Hips Relationship

END PHASE: Gently landing on the target surface and adjusting yourself to where you want to be seated.

- When you finish your transfer, you should feel that it was very smooth and well controlled.
- Both of your hands should be in contact with both surfaces at the end of the transfer.

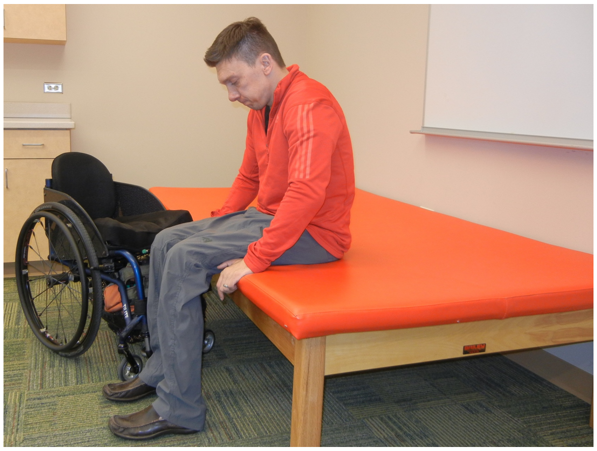
End Phase

Stand-Pivot Transfers

GET WISE Section: Transfers

If you are able to stand up while doing a transfer, you can use the same general instructions provided in the document "Independent Transfer Education," page 35. To modify the transfer, please talk with your trainer and follow these instructions:

Set Up Phase:

Step 1: Position Your Chair

- While you still want to get close to the surface you are transferring to, make sure that you have adequate room to maneuver your feet if you take a few steps when you transfer.

Step 2: Chair Set Up

- If you use your arm rests to push up from the chair, please don't remove them. You will have to decide if the arm rests are more useful to push up from or if they potentially might get in your way during the transfer. Please talk with the trainer to make this determination.

Step 3: Feet and Body Positioning

- No change.

Step 4: Hand and Shoulder Positioning

- Depending on how you perform the transfer, it might be safer for you to keep both hands on your chair to help you push up from. Then move one hand to the surface you are transferring toward.
- It is still critical to use a handgrip if one is available.

Flight Phase:

- You will not need to use the head hips relationship.

End Phase:

- No changes. Continue to exercise care when sitting down.

Assisted Transfer Education

GET WISE Section: Transfers

Learning how to properly perform transfers activities is very important, as transfers are performed on a frequent basis throughout the day and are needed to perform even very basic activities of daily living. Learning the proper techniques will help keep you safe and prevent falls and the development of upper extremity injuries. It is also very important to learn how to effectively work with a care partner if you receive assistance during transfers.

A transfer is divided into three phases: the set-up phase, flight phase, and end phase. The set--up phase is the longest portion of a transfer and involves the placement of your wheelchair/scooter and your body and getting your care partner in the proper position. Setting up the transfer well will make the flight and end phase of the transfer very easy. The flight phase of the transfer is the portion in which your care partner lifts your body and moves you to the new surface. Finally, the end phase involves safely landing on the target surface and adjusting your body.

Please review the points below about each phase of the transfer.

General Tips to Reduce the Frequency of Falls and Injury

- Perform a level transfer whenever possible.
  - Look for alternative surfaces to transfer to (not just the most convenient place), or adjust the surface (if possible).
  - Example: Adjust the height of your tub seat or bedside commode so you do not need to perform an uphill transfer every day.
- Alternate which arm you use to push off with.
  - Example: Don't always use your right arm as your push off arm – alternate between left and right.
- Use a transfer board or other assistive technology if you have difficulty performing transfers.
- Use a gait belt to give your care partner a solid surface to provide assistance.
- Try to make the transfer as easy as possible.

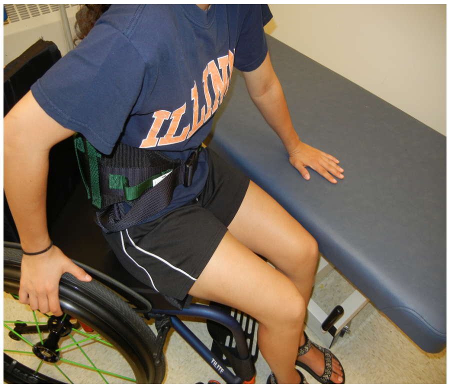

Gait Belt

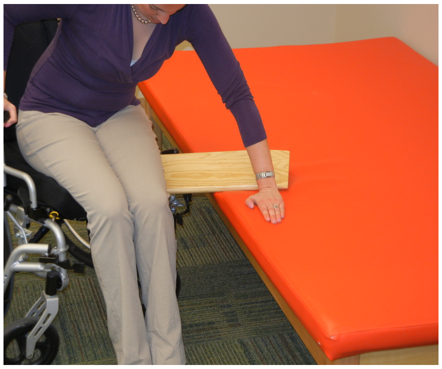

Transfer Board

SET UP PHASE: Position your chair and body properly for a safe and easy transfer.

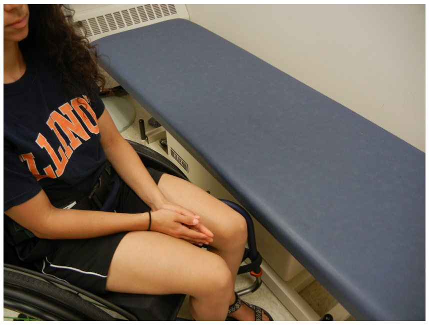

Position Your Chair

- **Step 1: Position Your Chair**
  - Your chair should be as close to the surface you are transferring to as possible. Ideally, the chair should be touching the surface.
    - Shorter distances decrease the potential for falls.
    - Shorter distances decrease the amount of time you have a significant amount of weight on your arms.
  - Position your chair with a 20-45 degree angle between your chair and the surface you are transferring to.
    - This position will help put your shoulder in the best alignment for the correct transfer position.
  - Put on your wheelchair/scooter brakes when you are happy with your position.

Step 2: Chair Set Up

- If you have an armrest or sideguards on your chair, take them out of the way before you transfer (if possible).

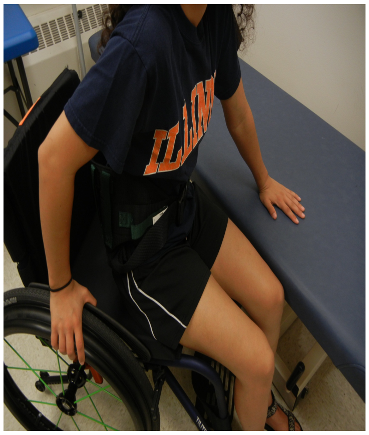
Body Position

- **Step 3: Body Positioning**
  - Place your feet in the most stable position, on the floor if possible, before you transfer. Even if you do not have control of your legs, putting them in the proper alignment will provide a small amount of support and stability.
  - Sit on the front 2/3 of your chair (or close to the edge of the surface you are transferring from). If necessary, your care partner can assist you to scoot forward in your chair by moving one side of your body forward at a time.
    - Once you are properly positioned, make sure your care partner provides you with support if you have trouble with balance.

- **Step 4: Hand and Arm Placement**
  - Place your hands in a stable position prior to the start of the transfer.
    - The hand that you will be pushing from is close to your body and holding onto a stable object.
    - Your other hand should be positioned close to where you intend to land. The angle between your arm and body should be approximately 45 degrees.
  - Use a handgrip, if possible.
    - You can use the armrest on your chair, the edge of a mat table/bed, etc.
    - If no handgrip is available, place your hand flat on the surface you are transferring from.
      - → Don't reach for a handgrip in a position that will make you unstable!

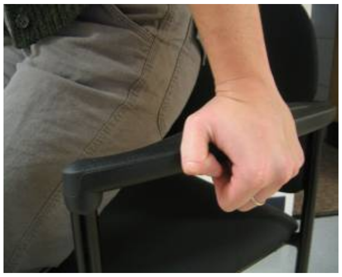

Best!

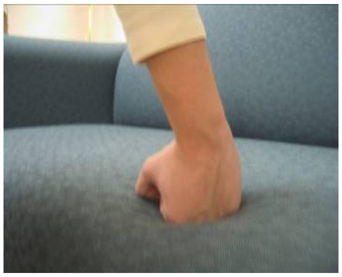

Try to Avoid

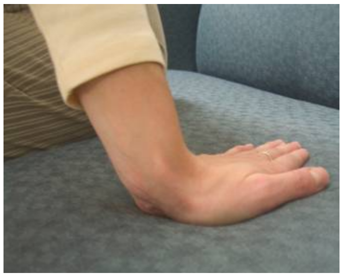

Try to Avoid

Step 5: Care partner Placement
- Instruct your care partner to:
  - Position her feet on either side of your feet
  - Squat down
  - Hold tightly onto gait belt
  - Tell your care partner to keep her back straight and lift with her legs

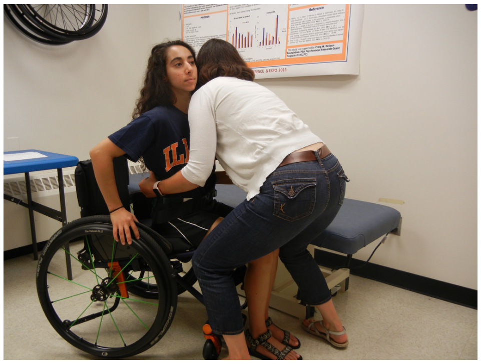

Caregiver Position

<u>FLIGHT PHASE:</u> Movement from point A to point B. If you have taken time to set up the transfer properly, this will be very easy.

- Use the "head-hips" relationship.
  - Move your head in the opposite direction that you want your hips to move. This makes the transfer easier and reduces the potential for falls.

- Make sure your care partner stays out of your way when trying to use the head hips relationship.

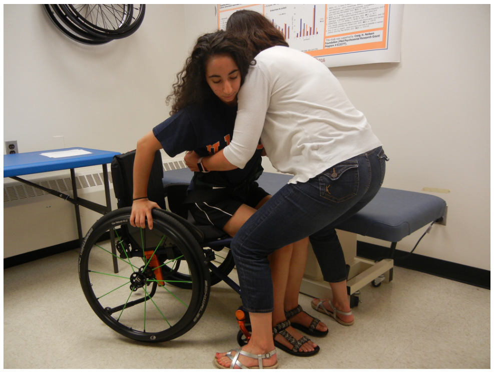

Head Hips Relationship

- Make sure to clearly communicate with your care partner and let him/her know when the movement should begin (counting can help assure everyone is on the same page).
- The flight should feel very smooth and well controlled.

END PHASE: Gently landing on the target surface and adjusting yourself to where you want to be seated.

- When you finish your transfer, you should feel that it was very smooth and well controlled.
- Both of your hands should be in contact with both surfaces at the end of the transfer.
- Make sure your care partner properly positions you before letting go!

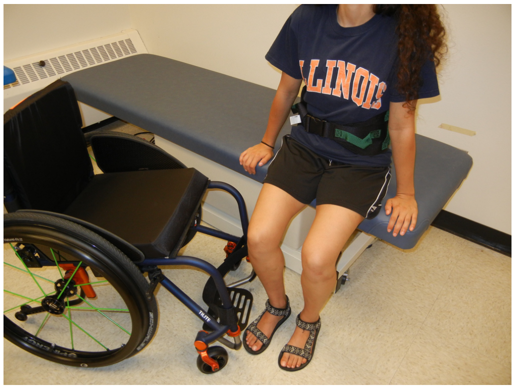

End Phase

Influence of the Environment
GET WISE Section: Environment

Identifying Environmental Hazards

Living with potential fall hazards in the environment is a reality we all have to live with, but there are strategies you can use to manage your fall risk. Falls prevention starts with paying attention, being aware of your surroundings, and identifying potential fall risks around you.

Point of discussion: *Can someone give me one or two examples of an environmental hazard that contributed to a fall they had?*

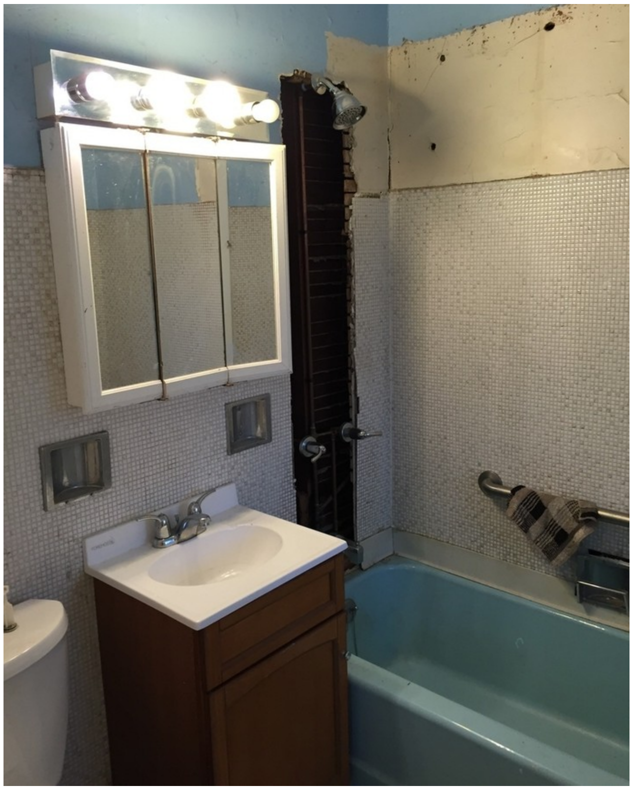

Figure 1 -- Environmental Hazard "Before" Modifications
Source: Rebuilding Together

Point of discussion: What hazards or potentially dangerous situations do you see?

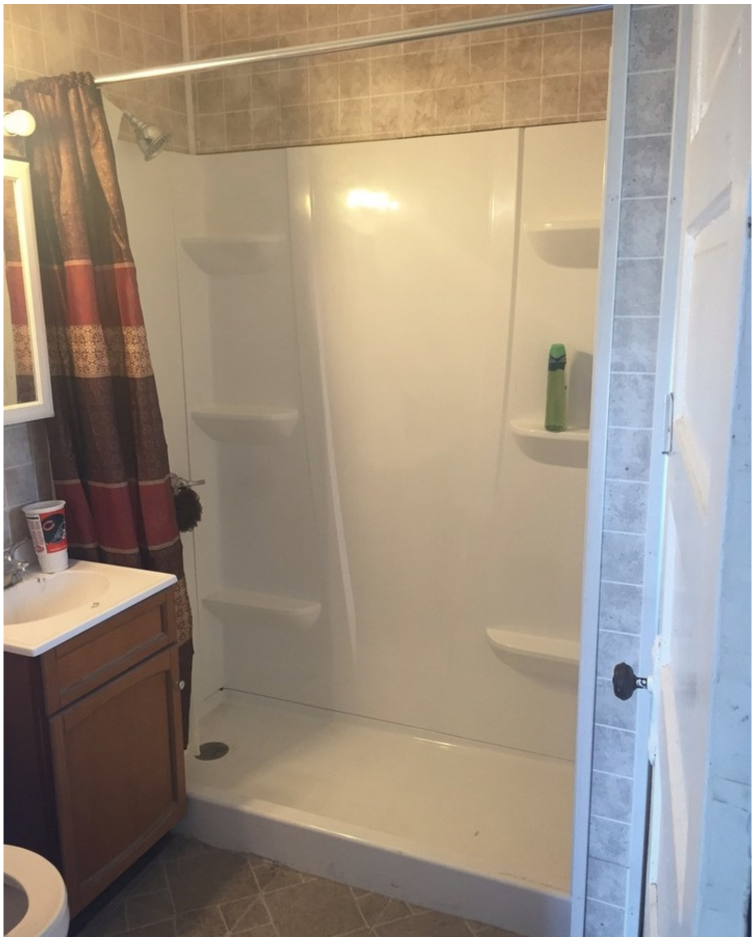
Figure 2 -- Environmental Hazard Picture "After" Modifications

Key changes:

- Low rise to enter.
- Higher shelving for shower items (to avoid bending over).
- This is accessible for the individual it was made for, but there are a number of other modifications that could be made based on personal need. Maybe a shower chair/tub transfer bench, an anti-skid mat on the floor, a handheld shower, a grab bar, a long-handled sponge, or a curbless walk-in shower.

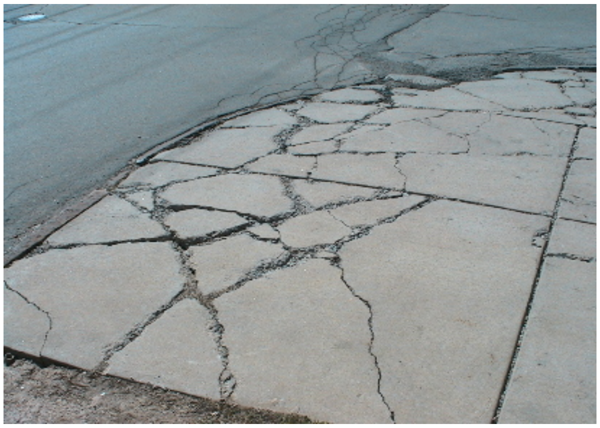

Point of discussion: *Would this be a hazard for you?*

This ability to know your capability, and to compare it to the demands of the environment is an important skill!

Figure 3 – Potential environmental hazard: Cracked sidewalk

When we think about what makes an environmental hazard, it's the combination of the person and the environment.

Person + environment

Now, let's take a look at the steps to <u>manage</u> identified environmental hazards.

<u>Managing Environmental Hazards</u>

Once you have identified fall risks in your surroundings, the next step is to make a plan of action.

Environmental risk factors fall in one of two categories:

1) **Environmental hazards requiring immediate action:** These are hazards you have to manage in the moment.

<u>Example</u>: You are at a friend's house and need to use the bathroom. You typically use a grab bar to help you transfer to the toilet, but there isn't one and the toilet is quite low.

2) **Environmental hazards requiring long-term planning:** These are hazards, typically in your home, that require you to take a series of steps over time to address the problem. We saw an example in the "before and after photo" from the bathroom.

For environmental hazards that require <u>**immediate action**</u>, you can use the "Stop, Plan, Act" strategy.

- <u>Stop:</u> Pay attention, assess the environment, identify situations that could be unsafe.
- <u>Plan</u>: Determine safe options to address the unsafe situation. Select the best plan for you.

- Act: Move forward with your safety plan.

Let's take a look at 2 scenarios that require immediate action.

* Outdoor questionable sidewalk: Let's say that you're approaching the sidewalk pictured below. You notice there is spot that appears uneven and steep. What do you do?

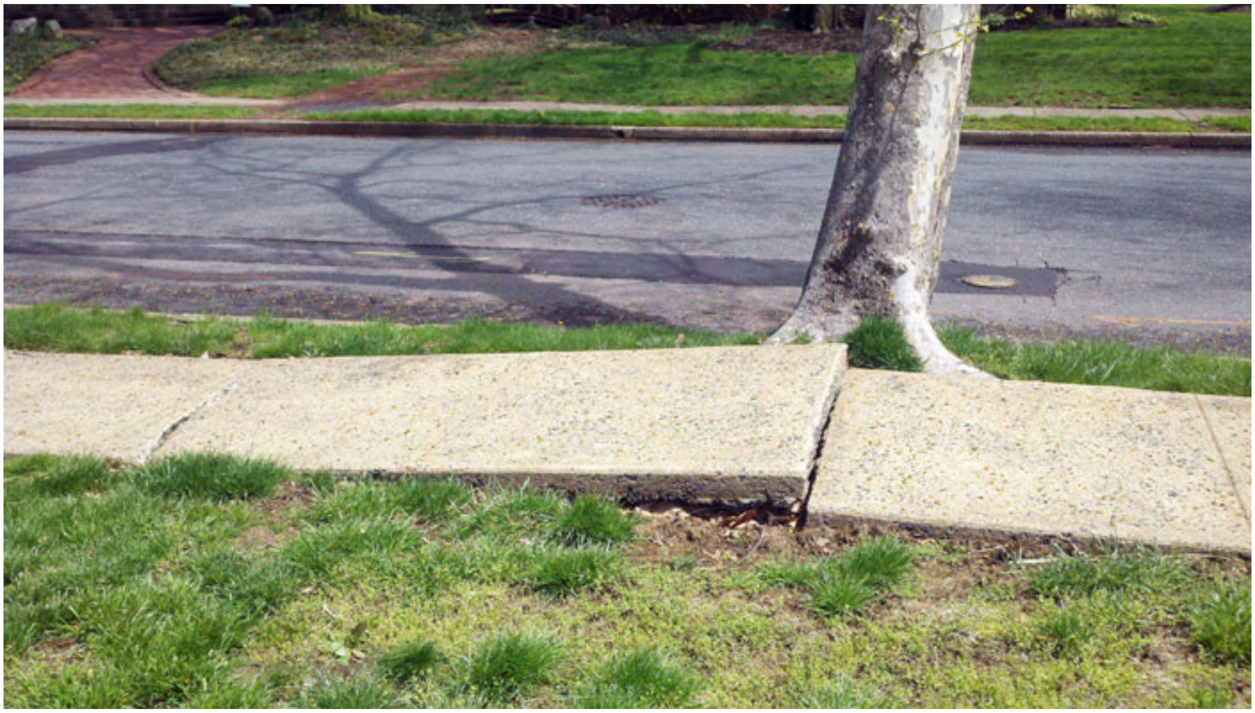

Figure 4 – Potential environmental hazard: Questionable sidewalk

* Indoor questionable toilet: Let's go back to the situation where you are visiting your friend's home and you need to use the restroom. Once in the bathroom, you notice it is quite a low toilet and there are no grab bars. What do you do?

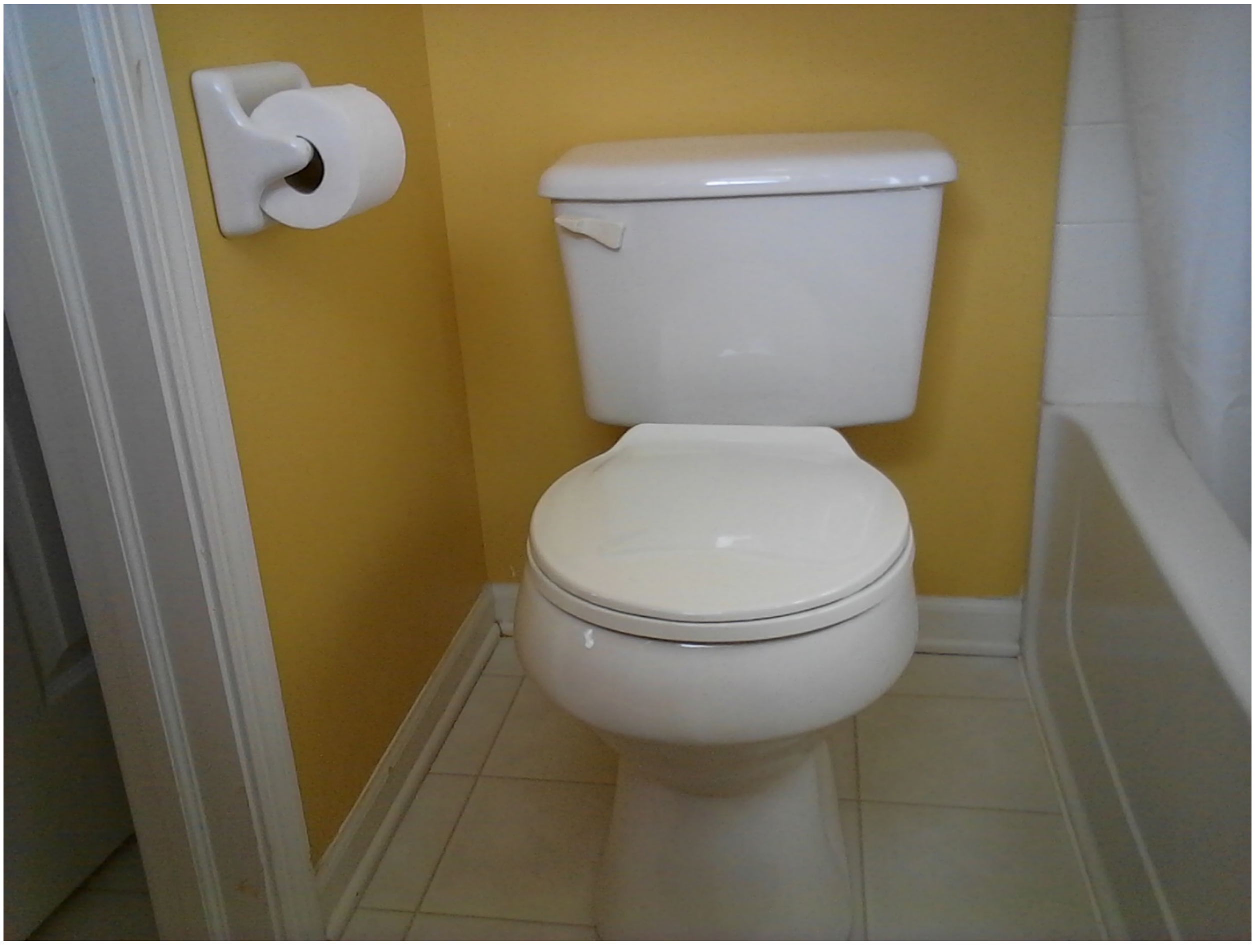

Figure 5 – Potential environmental hazard: Questionable Toilet

For environmental hazards that require **long-term planning**, we are going to learn about a strategy called **action planning**. An action plan is a way to break down a large goal of yours into small, manageable steps.

ACTION PLANNING FORM
SAMPLE FOR TRAINER

Think about what could help you prevent a fall in your home or a place where you have fallen frequently. Think about how you could address this area and minimize the fall risk.

Step 1: What is your environmental modification goal?

Get a ramp to enter the house through front or back door

Step 2: What is one small step you could towards your goal this week?

Investigate funding options

What will you do?

Call local MS chapter to ask if grants are available OR if they can refer me to other funding sources

When will you do it?

Tomorrow

Step 3: Identify what will help you succeed:

Set phone timer to go off at noon tomorrow as a reminder to get this done before 1pm

Step 4: How confident are you that you can succeed in this step in the next two weeks?
1 2 3 4 5 6 7 8 9 10
(Not sure) (very sure)

*You should be an eight or higher on this step. If you're not higher than an eight, let's take a closer look at the goal you have set.
===
Step 5: Assess your progress on step #1.

Step 6: Identify specific, manageable next steps (then repeat Steps 2 through 5 until complete).

Adapted from © Self-Management Resource Center, LLC, 1980 - 2017. All rights reserved. All or portions of this material include copyrighted materials belonging to Self-Management Resource Center. To obtain a license please contact the Self-Management Resource Center. A Self-Management Resource Center Program (SMRC) developed by Dr. Kate Lorig, Virginia González and Diana Laurent.

Key part of the action plan:

- The first step is to consider a manageable goal that you are confident of accomplishing in a week.
- Your level of confidence should be an eight or higher, or you should possibly reconsider the goal.
- The action plan helps you break down a bigger task into manageable, thought out steps.

There are 10 copies of blank action plans in your supplemental folder.If you need more, please feel free to make copies or print one from the study website.

Remember that often simple, inexpensive additions to the environment can make a difference in safety and possibly prevent a fall.

Let's take a look at the "Fall proofing your home and environment" checklist on the "Minimizing Your Risk of Falls" brochure (Page 9).

Note: You can also view the brochure online: https://www.nationalmssociety.org/NationalMSSociety/media/MSNationalFiles/Brochures/Brochure-Minimizing-Your-Risk-of-Falls.pdf (Frankel & Schneider, 2016).

This handy guide supports you as you carefully assess the environment around you for fall risks. Keep in mind, these are just some general safety principles.

We would like you to complete the "Fall proofing your home and environment" checklist on pages 9 through 16 in your booklet over the course of the next week. We hope this activity will support your personal assessment of your environment.

Before Session 3:

- Please complete the "Fall proofing your home and environment" checklist on pages 9-16, based on your review of your home.
- Complete one action plan.

In Session #3 we will share updates on the focus of our action plan and progress to date.

We will be talking about funding options for home modifications/equipment in a future session. In the meantime, please turn to page 50 in your participant manual. There you will find:

- Examples of home modifications
- Home modifications and fall prevention resources

Home Modification Resources:

Example of home modifications. Modified from Sabata & Lowenstein's (2013) book chapter, "Physical Environment" (pp. 487)

Activity type	Examples of home modifications
Moving around	Motion-sensor lighting, manual wheelchair, power wheelchair, scooter (Sabata & Lowenstein, 2013), power chair lift for stairs (Frankel & Schneider, 2016), transfer board
Toileting	Raised toilet set, properly installed grab bars, commode chair (Sabata & Lowenstein, 2013), toilet safety frame
Bathing	Tub transfer bench (Sabata & Lowenstein, 2013), properly installed grab bars, hand-held shower (Trudeau, 2016), non-slip bath mat, long handled sponge
Dressing	Reacher, long handled shoe horn dressing stick (Sabata & Lowenstein, 2013), elastic shoe laces, sock aide
Sleeping	bedside commode, nightlight, lamp within arm's reach at bedside
Leaving the home	Accessible ramp for entrance (Sabata & Lowenstein, 2013), outdoor motion-sensor lighting

Home modifications and falls prevention resources:

Agency	Website	Summary of information
American Occupational Therapy Association, 2014 (AOTA)	https://www.aota.org/~/media/Corporate/Files/Practice/Aging/Resources/Focus-On-Falls-Prevention-Home-Mod-Booklet.pdf	This compilation of articles provides a summary of current information on home modifications and falls prevention.
AOTA (2018)	https://www.aota.org/About-Occupational-Therapy/Patients-Clients/Adults/Falls/prevent-falls-in-home-tips.aspx	This 22-minute video provides tips from an occupational therapist to prevent falls in your home.
Falls Prevention Center of Excellence	http://stopfalls.org/resources/home-modification-tools-programs-and-funding-landingpage/	Under the "Individuals and Families" section, you will find a wealth of information on preventing falls, including assessing the need for and sources for funding home modifications.

National Council on Aging (Trudeau, 2016)	https://www.ncoa.org/blog/falls-prevention-home-18-step-safety-checklist/	This link provides simple changes for your home that reduce your risk of falling.
National Multiple Sclerosis Society (NMSS)	https://www.nationalmssociety.org/Resources-Support/Library-Education-Programs/Free-From-Falls/Staying-Safe-at-Home<br><br>https://www.nationalmssociety.org/NationalMSSociety/media/MSNationalFiles/Resources_Support/Handout-Week-5.pdf<br>{Handout to accompany video}	This video and handout focus on how to reduce your risk of falls in the home. Specifically:<br>• "Explore possible hazards that might contribute to falls inside and outside the home<br>• Understand how simple changes can make your home environment safer<br>• Identify specific changes you can make to your home environment to make it safer" (National Multiple Sclerosis Society, 2018a)
NMSS	https://www.nationalmssociety.org/NationalMSSociety/media/MSNationalFiles/Resources_Support/Handout-Week-5.pdf<br><br>https://www.nationalmssociety.org/NationalMSSociety/media/MSNationalFiles/Resources_Support/Handout-Week-6.pdf<br>{Handout to accompany video}	This handout and video focus on how to reduce your risk of falls in the community. Specifically:<br>• "Recognize common causes of slips, trips, and falls<br>• Be aware of fall hazards in your community and how to navigate the community safely<br>• Employ the Stop, Scan and Plan technique to identify and navigate fall risks in your community" (National Multiple Sclerosis Society, 2018b)

References:

Sung, J., Trace, Y., Peterson, E. W., Sosnoff, J. J., & Rice, L. A. (2017). Falls among full-time wheelchair users with spinal cord injury and multiple sclerosis: A comparison of characteristics of fallers and circumstances of falls. *Disability and Rehabilitation*, , 1-7.

10.1080/09638288.2017.1393111 Retrieved from https://doi.org/10.1080/09638288.2017.1393111

Frankel, D., & Schneider, D. (2016). *Minimizing Your Risk of Falls: A guide for people with MS.* National Multiple Sclerosis Society. Retrieved from: https://www.nationalmssociety.org/NationalMSSociety/media/MSNationalFiles/Brochures/Brochure-Minimizing-Your-Risk-of-Falls.pdf

Sabata, D., Lowenstein N. (2013). Physical Environment. In M. Finlayson (Ed.), *Multiple Sclerosis rehabilitation: From impairment to participation* (pp.477-496). Boca Raton, FL: CRC Press, Taylor & Francis Group.

Tennstedt, S., Peterson, E., Howland, J. & Lachman, M. (1998). *A Matter of Balance: Managing concerns about falls facilitator manual.* Boston, MA: Roybal Center Consortium.

American Occupational Therapy Association (2014). *Focus on: Falls prevention and home modifications.* Retrieved from: https://www.aota.org/~/media/Corporate/Files/Practice/Aging/Resources/Focus-On-Falls-Prevention-Home-Mod-Booklet.pdf

American Occupational Therapy Association (2018). *How to prevent falls in the home: Tips from AOTA and the National Council on Aging.* Retrieved from: https://www.aota.org/About-Occupational-Therapy/Patients-Clients/Adults/Falls/prevent-falls-in-home-tips.aspx

Falls Prevention Center of Excellence (2018). *Home Modifications Resource Inventory.* Retrieved from: http://stopfalls.org/resources/home-modification-tools-programs-and-funding-landingpage/

National Multiple Sclerosis Society (2018a). *From Free from Falls: The National MS Society's Comprehensive Falls Prevention Program. Session 5: Staying safe in your home.* Retrieved from: https://www.nationalmssociety.org/Resources-Support/Library-Education-Programs/Free-From-Falls/Staying-Safe-at-Home

National Multiple Sclersosis Society (2018b*). From Free from Falls: The National MS Society's Comprehensive Falls Prevention Program. Session 6: Preventing slips, trips and falls: Staying safe in the community.* Retrieved from: https://www.nationalmssociety.org/NationalMSSociety/media/MSNationalFiles/Resources_Support/Handout-Week-5.pdf

Trudeau, S. (2016). *18 Steps to Fall Proofing Your Home.* National Council on Aging. Retrieved from: https://www.ncoa.org/blog/falls-prevention-home-18-step-safety-checklist/

ACTION PLANNING FORM
iRoll Falls Prevention Program

Think about what could help you prevent a fall in your home or a place where you have fallen frequently. Think about how you could address this area and minimize the fall risk.

Step 1: What is your environmental modification goal?

____

____

Step 2: What is one small step you could make towards your goal this week?

____

____

What will you do?

____

____

When will you do it?

____

____

Step 3: Identify what will help you succeed:

____

____

Step 4: How confident are you that you can succeed in this step in the next 2 weeks?

1 2 3 4 5 6 7 8 9 10
(Not sure) (very sure)

*You should be an eight or higher on this step. If you're not higher than an eight, let's take a closer look at the goal you have set.

==

Step 5: Assess your progress on Step #1.

____

____

Step 6: Identify specific, manageable next steps (then repeat Steps two through five until complete).

____

____

Adapted from © Self-Management Resource Center, LLC, 1980 - 2017. All rights reserved. All or portions of this material include copyrighted materials belonging to Self-Management Resource Center. To obtain a license please contact the Self-Management Resource Center. A Self-Management Resource Center Program (SMRC) developed by Dr. Kate Lorig, Virginia González and Diana Laurent.

Session #2 Journal Entry

What will be done:

You will be asked to make a journal entry every week after completion of a session. Your journal entry will be tied to items discussed during the previous session. You will typically be asked to respond to a specific question regarding the content of the program from the previous session.

Why:

The journal entries will help you think about the information that was presented during the education sessions and help you integrate what you have learned into your everyday life.

Prompt:

Please describe an environmental hazard that might increase your fall risk in your home. Then, write about your experiences regarding your first action plan. What efforts did you make to complete your first action plan and how did it go? If you could not complete your first action plan, what prevented you from completing the task?

Response:

Wheelchair/Scooter Skills 101

GET WISE Section: Wheelchair Management

The ability to push or drive your wheelchair/scooter effectively (wheelchair/scooter skills) is necessary to allow you to safely and successfully navigate a variety of environments in your home and community. Having good wheelchair/scooter skills is also very important when it comes to preventing falls. There are a variety of wheelchair/scooter skills related to the daily activities you perform, such as pushing or driving your wheelchair/scooter over even and uneven surfaces, navigating narrow environments, going up/down curbs, etc.

Point of discussion: Have you ever experienced a fall while pushing or driving your wheelchair/scooter? How did it occur?

Why is learning how to properly perform wheelchair/scooter skills important?

- Falls often occur while pushing or driving a wheelchair/scooter. Specifically, falls frequently occur when people drive or push their wheelchair/scooter in a hurry, while not paying attention to the surrounding environment, or going up and down a steep ramp.

- Good wheelchair/scooter skills are needed to perform necessary daily activities such as getting to the bathroom, shopping for food, and going to doctor's appointments.

- Good wheelchair/scooter skills will also help you get out into the community and do the activities you wish to do. For example, if you would like to go to the library but are unsure how to navigate a ramp, it might be difficult or scary for you to get into the building. If you learn how to push/drive up a ramp, you might feel more confident going to the library.

- With good wheelchair/scooter skills, you might also be able to do more things by yourself and will not need to rely on a care partner to assist you as often.

- Knowing how to use a wheelchair/scooter correctly will give you the ability to manage unexpected items such as a crack in the sidewalk or other unexpected situations.

General tips for safety

Please see the information below for some tips on safe and efficient wheelchair/scooter skill performance:

- **Fasten your seat belt!** The seat belt will help you to stay in your chair if you accidently hit an unexpected obstacle such as a bump or a curb.

- **Control your speed!** Even if you are in a hurry, push or drive your chair at a reasonable speed. Wheelchair/scooter users frequently report that falls occurred because they were pushing or driving the chair too fast and hit an obstacle.

- **Check the pathways you often travel to make sure they are clear and accessible.** Ask your care partner to arrange the furniture in your house to create a clear path. Also, keep the floors clear of small items. Random obstacle such as a dog, toys or electrical cords can cause a fall.

- **Maintain your wheelchair/scooter regularly!** Wheelchair/scooter malfunctions, such as broken seat belts, wheel locks (brakes) or footplates, are associated with falls. Regular wheelchair/scooter maintenance can help prevent these problems from occurring. This concept will be discussed further during Session #5 (Page 130).

- **Don't drive or push your wheelchair/scooter when you are intoxicated.** It is extremely dangerous. If you have consumed alcohol, ask someone who has not been drinking to take you to where you want to go.

During this education program, you will learn and review how to perform important wheelchair/scooter skills, ranging from basic skills to more complicated tasks. Today, we will start with basic wheelchair/scooter skills. Instruction on the performance of these skills can be found on the following pages:

1) Basic skills: Manual: please see page 58, Power/Scooter: please see page 63

2) Intermediate skills: Manual: please see page 98, Power/Scooter: please see page 101

3) Advanced skills: Manual: please see page 111, Power/Scooter: please see page 116

You can also find videos of the wheelchair/scooter skills on the study website. These videos will help you visualize the techniques.

Please be patient with yourself while learning these skills! Wheelchair/scooter skills can be challenging, and you might be asked to consider changing how you are doing some of your current skills. The trainer will work with you to refine the skills you currently have and learn new ways to navigate your community.

Note: Some of the wheelchair/scooter skills are difficult to learn and require a lot of practice. If you feel that you would like to get additional practice and/or feedback on your skills after the education program is over, please talk to your physician. They may provide a referral to a Physical or Occupational therapist, who will further help you learn these skills.

Manual Wheelchair Skills Education (Basic)

Get WISE Section: Wheelchair Management

Today you will learn and review some basic wheelchair skills to allow you to safely and successfully navigate a variety of environments in your home and community. We will discuss how to push your wheelchair on a flat surface, reach for an object, pick up an object off the floor, and go through a hinged door.

Pushing a wheelchair on a flat surface

The ability to push your wheelchair effectively requires a combination of good propulsion technique and optimal wheelchair design. A physical or occupational therapist who specializes in wheelchair seating can ensure that your wheelchair is set up for optimal propulsion. This topic will also be discussed further during Session #5 (Page 123). Learning how to perform this basic skill correctly will help you to prevent falls and upper extremity injuries from occurring.

Name of video: Pushing a wheelchair on a flat surface
Start: 1:07
End: 2:40

General tips to reduce the frequency of falls and injury:

During pushing

- Focus your attention in the direction of travel, avoiding distractions to either side, but remain alert to potential hazards.
- Control your speed!
- Use good propulsion techniques. Please review the points below.

Good propulsion techniques

- Begin the push with your hands on the upper back part of the hand rim.
- Use long, smooth strokes.
- Do not rush your stroke. Take your time moving the wheel forward and try to keep in sync with the speed of the wheel.
- Release your grip toward the front of the wheel.
- Once you have released the wheel, let your hand gently relax and return to the upper back of the wheel.
- Push less often by using longer pushes.
- The overall shape of your propulsion technique should be an oval, as pictured below.

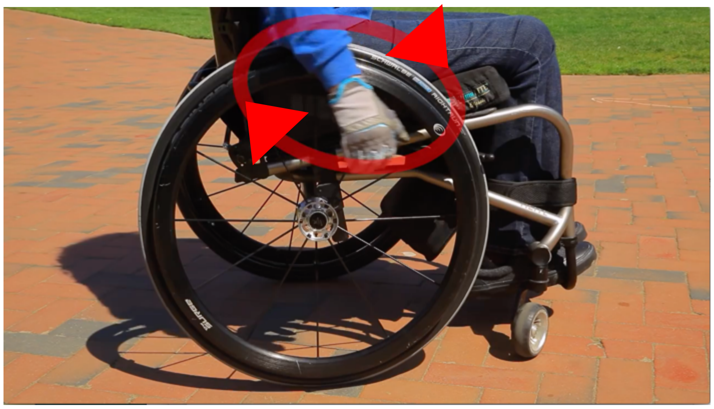

Figure 1: The oval shape propulsion pattern

Reaching for an object out of your range

You may often have to reach upward or forward for a light switch, elevator button, or to get an object out of a cupboard. Reaching and leaning reduce stability and puts you at risk of falling out of the wheelchair.

Name of video:

- Reaching for an object out of your range (no standing)
- Reaching for an object out of your range (standing)

General tips to reduce the frequency of falls and injury

- Position your wheelchair close to the object you are reaching for.
- Reach to the side instead of reaching forward, if possible.
- Secure the seatbelt.
- Engage wheel locks/brakes before you begin reaching.
- Stabilize yourself with one hand holding onto the armrest or wheel as you reach for the object with the other.
- Use a reaching aid (please see picture on page 60) if it is available.

If you stand up

- First apply the brakes and clear the footrests out of the way.
- Please DO NOT stand on your footrest. The extra weight on the footrests may cause the chair to tip and a fall to occur.
- Keep one hand on the wheelchair to maintain your balance.

Picking up an object off the floor

You may need to pick up an object from the floor or ground, such as a piece of paper or a key. Attempting to pick up objects from the floor by reaching down reduces stability and puts you at risk of falling out of the wheelchair.

Name of video: Picking up an object off the floor

General tips to reduce the frequency of falls and injury

- Position your wheelchair close to the object you are reaching for.
- Secure the seatbelt.
- Engage wheel locks/brakes before you begin reaching.
- Stabilize yourself with one hand on the push rim or arm rest as you pick up the object with the other hand.
- If you have weak trunk muscles, move the arms to the thighs one at a time, and then to the feet, placing the chest on the thighs to reach the ground.
- Pull the object up against one of the wheels to increase stability and make the task easier.
- Use a reaching aid if it is available.

If you are hesitant to reach down to pick up an object on the ground or to get an object off a high shelf because of poor sitting balance, a reacher can be a good option (figure 2). A reacher enables you to easily reach an object in high places and pick up things off the ground without having to bend your body. You can buy a reacher at amazon.com, Target or Walmart.

To use a reacher, hold the handle and move the other end of the reacher in the direction of the item you wish to pick up. Once in position, squeeze the handle to grasp the item and move it to wherever you like.

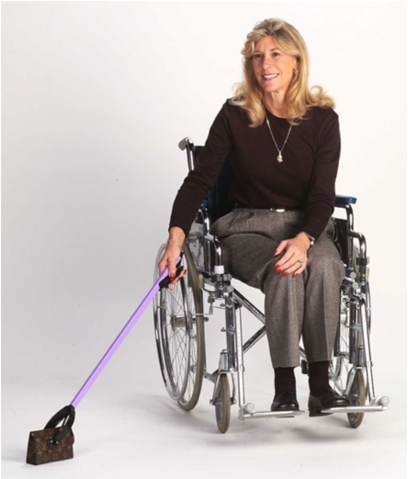

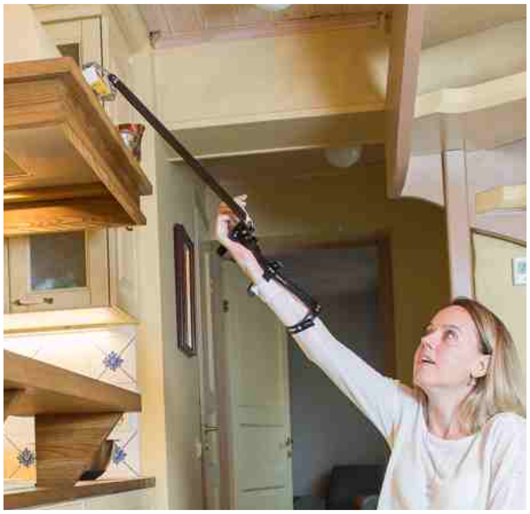

Figure 2: Using a reacher to pick up an object

Going through a hinged door

You often have to open/go through a hinged door to perform necessary and desired activities in your daily life. However, while performing this activity, you can easily lose your balance and fall.

Name of video: Going through a hinged door (Slide 8)
Start: 0:26
Stop: 1:47

General tips to reduce the frequency of falls and injury

- Approach the door at an angle. This brings the door handle into reach and gives you enough space to clear the door with the front of your wheelchair.
- Stabilize yourself with one hand on the hand rim or arm rest as you reach for the door with the other.
- Pay attention if there are any obstacles (e.g. threshold) while passing through the door.

When pulling the door open

- Pull the door open with the hand closest to the door as you balance yourself with your other hand on the wheel.
- Keep the door open with your hand or elbow as you push your wheelchair through. If the door is especially heavy, pull on the door handle with one hand while pushing on the doorframe with the other.

To close the door

- You may gently swing the door closed behind you, moving the wheelchair quickly through the door and out of the way.
- Alternatively, turn around once through the doorway, reach forward, and pull the door towards you while backing away.
- Don’t put your fingers between the door and doorframe for any longer than necessary because you may get pinched when the door closes.

When pushing the door open

- Keep pushing one wheel forward with one hand as you push the door open with the other.
- While moving past the door, pay attention to avoid catching any clothing or body parts on the door handle or the surface of the door.

To close the door

- You may gently swing the door closed.
- Alternatively, turn the wheelchair around and push the door closed with the footrests.
- Back up to close the door using the rear wheel or other wheelchair part to push on the door.

<u>Power Wheelchair and Scooter Skills Education (Basic)</u>

Get WISE Section: Wheelchair Management

Today you will learn and review basic power wheelchair and scooter skills that will allow you to safely and successfully navigate a variety of environments in your home and community. We will discuss how to drive your power wheelchair or scooter on a flat surface, reach for an object, pick up an object off the floor, and go through a hinged door.

<u>Driving Forward and Backwards</u>

The ability to drive your power wheelchair or scooter effectively is necessary to allow you to safely and successfully navigate a variety of environments in your home and community. Learning how to perform this basic skill correctly will help you prevent falls.

<u>Name of video:</u> Power Wheelchair Skills
Start: 2:03
End: 2:25

General tips to reduce the frequency of falls and injury

- Get to know your wheelchair or scooter.
  - How to control the joystick or throttle
  - How to select drive modes and speeds
  - If you have questions about how to operate anything on your chair, please ask the trainer or the vendor that sold you the wheelchair or scooter.
- Make sure that nothing is stuck in your wheels, such as the seat belt or other straps.
- Fasten the seat belt!
- Make yourself as visible as possible by wearing brightly colored clothing and something reflective at night. You might also want to consider putting reflective markers on your chair or scooter.
- Check the pathways you often travel to make sure they are clear and accessible.

During Driving forward

- Move the joystick or throttle forward gradually to achieve a smooth start.
- Pay attention in the direction of travel and remain alert to potential hazards. Avoid distractions whenever possible.
- Control your speed!

During driving in reverse

- Check behind you before you reverse. Consider the presence of moving objects, such as people or cars.
- Move the joystick or throttle backwards gradually to achieve a smooth start.

- Control your speed!
- Don't drive in reverse for a long distance.

Scooter Consideration

- Be ready to drive the scooter before turning the power on.
  - Place both hands on the handlebars (tiller) and position both feet on the platform. Make sure your feet are not hanging off the side of the scooter.
  - Fasten the seat belt!
- Use the same techniques as described above.
- Take some time to learn to use the throttle. Depending on your scooter design, you might use one hand to drive forward and another to drive backwards. Practice using the controls in a safe environment so that you fully understand how to use the device before you are in a challenging situation.
- Driving in reverse may be challenging due to the orientation of the throttle. Take the time to practice in a safe environment.

<u>Turning in place</u>

Learning how to turn in tight spaces can give you confidence to maneuver in small spaces such as elevators and bathrooms.

<u>Name of video</u>: Power Wheelchair Skills
Start: 2:03
End: 2:25

General Tips to reduce the frequency of falls and injury

- To make the tightest turn possible **in a power wheelchair**, point the joystick in the direction you want to turn. If you think of your controller as a clock with '12' being straight ahead, point the joystick to either '3' or '9', depending if you want to turn right or left.
- Know your wheelchair configurations. The location of the drive wheels (e.g. rear-wheel, mid-wheel or front-wheel drive) and seating configurations (e.g. foot rest) can affect the size of the turning radius. Before trying to maneuver in a tight space, become familiar with the turning radius of your chair.
- Control the speed for turning of the chair.
- The vertical axis of rotation for such a turn should be midway between the drive wheels.

Scooter Considerations

- ➢ Scooters cannot turn in place in the same way that a power wheelchair can. To make a tight turn, you will need to move your scooter back and forth several times ("3-point turn") to make a tight turn.
- ➢ Select the slower speed mode before maneuvering in tight spaces.
- ➢ Please note: Scooters can easily tip over when making tight turns. Make sure that you are on a flat surface and moving slowly.

<u>Reaching for an object out of your range</u>

You may often have to reach upward or forward for a light switch, elevator button or to get an object out of a cupboard. Reaching and leaning reduce stability and puts you at risk of falling out of the wheelchair.

<u>Name of video:</u>

- Reaching for an object out of your range (no standing)
- Reaching for an object out of your range (standing)

General tips to reduce the frequency of falls and injury

- ➢ Position your wheelchair or scooter close to the object you are reaching for.
- ➢ Reach to the side instead of reaching forward, if possible.
- ➢ Secure the seatbelt.
- ➢ Turn the power off before you begin reaching.
- ➢ Stabilize yourself with one hand holding onto the armrest or wheel as you reach for the object with the other.
- ➢ Use your wheelchair functions. If your wheelchair has power seat functions (e.g. tilt, recline or seat elevator), this may be helpful.
- ➢ Use a reaching aid if it is available.

If you stand up

- ➢ First turn the power off and clear the footrests out of the way.
- ➢ Please DO NOT stand on your footrest. The extra weight on the footrests may cause the chair to tip and a fall to occur.
- ➢ Keep one hand on the wheelchair to maintain your balance.

Scooter Considerations

- ➢ Use the same techniques as described above but please note that **<u>scooters can tip over very easily.</u>**
- ➢ If possible, rotate your seat towards the object you are reaching for. Thus, you will be reaching forward for the object. Limit how far you reach forward. If you are able, rotate your seat and stand up to reach the object. Leaning while sitting on a scooter can be very dangerous!

- If you need to get out of the scooter, keep at least one hand on the scooter for balance.

Picking up an object off the floor

You may need to pick up an object from the floor or ground, such as a piece of paper or a key. Attempting to pick up objects from the floor by reaching down reduces stability and puts you at risk of falling out of the wheelchair.

General tips to reduce the frequency of falls and injury:

- Position your wheelchair close to the object you are reaching for.
- Secure the seatbelt.
- Turn off the power before you begin reaching.
- Stabilize yourself with one hand on the arm rest as you pick up the object with the other hand.
- If you have weak trunk muscles, move the arms to the thighs one at a time, and then to the feet, placing the chest on the thighs to reach the ground.
- If you have seat functions on your wheelchair, make sure you are fully upright and not tilted backwards.
- Use a reaching aid if it is available.

Scooter Considerations

- Use the same techniques as described above
- If possible, get out of your scooter before attempting to reach for something on the ground. **Remember, scooters tip over very easily!**
- If you need to get out of the scooter, keep at least one hand on the scooter for balance.

If you are hesitant to reach down to pick up an object on the ground or to get an object off a high shelf because of poor sitting balance, a reacher can be a good option (figure 1). A reacher enables you to easily reach an object in high places and pick up things off the ground without having to bend your body.

To use a reacher, you hold the handle and move the other end of the reacher in the direction of the item you wish to pick up. Once in position, squeeze the handle to grasp the item up and move it to wherever you like.

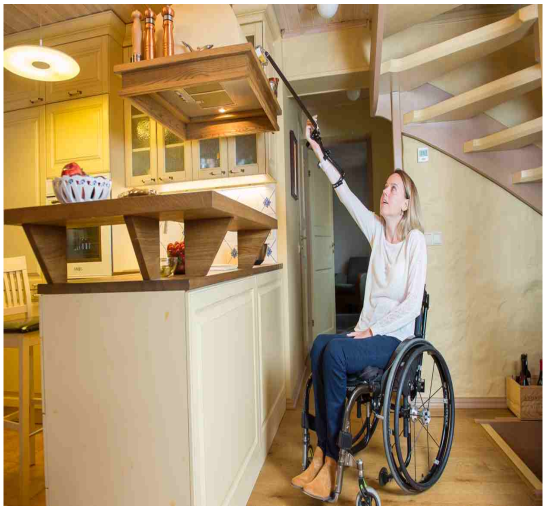
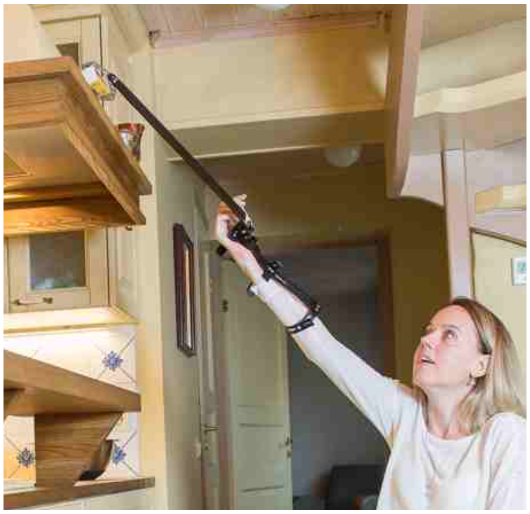

Figure 1: Using a reacher to pick up an object

Going through a hinged door

You often have to open/go through a hinged door to perform necessary and desired activities in your daily life. However, while performing this activity, you can easily lose your balance and fall.

Name of video: Power Wheelchair Skills
Start: 3:35
End: 4:10

General tips to reduce the frequency of falls and injury

- Approach the door at an angle. This brings the door handle into reach and gives you enough space to clear the door with the front of your wheelchair.
- Stabilize yourself with one hand on the arm rest as you reach for the door with the other. It may be easier to simply grasp the door handle with the hand on the side away from the joystick and then back the wheelchair up, rather than doing all of the work with the arm.
- Pay attention if there are any obstacles (e.g. threshold) while passing through the door.

When pulling the door open

- Pull the door open with the hand on the side away from the joystick as you control the joystick with your other hand.
- Keep the door open with your hand or elbow as you drive your wheelchair through.

To close the door

- You may gently swing the door closed behind you, moving the wheelchair quickly through the door and out of the way.
- Alternatively, turn around once through the doorway, reach forward, and pull the door towards you while backing away.
- Don't put your fingers between the door and doorframe for any longer than necessary because you may get pinched when the door closes.

When pushing the door open

- Push the door open with the arm on the side away from the joystick.
- While moving past the door, pay attention to avoid catching any clothing or body parts on the door handle or the surface of the door.

To close the door

- You may gently swing the door closed.
- Alternatively, turn the wheelchair around and push the door closed with the footrests.
- Back up to close the door using the rear wheel or other wheelchair part to push on the door.

Scooter Considerations

- Use the same techniques as described above.
- The length of some scooters can make it difficult to reach door handles. As a result, you may need to get off the scooter to open/close a door.
- If you are off the scooter, don't try to drive the scooter through the door. Use a rubber door stop to keep the door open or ask a care partner/bystander to help you.
- If getting on/off the scooter is becoming challenging, you may want to consider the use of a power wheelchair.

Session #2 Goal Form

GET WISE Section: Goals

Today's session focused on learning fundamental transfer and wheelchair/scooter skills. Please think about a goal that you can set that will help you practice either the transfer and/or wheelchair/scooter skills you learned today. We would like you to set at least one goal but feel free to set both a transfer and wheelchair/scooter skills goal. Don't forget to use the SMART goal format!

S	Specific.
M	Measurable.
A	Achievable.
R	Relevant.
T	Time bound.

Examples:

Over the next week, I will specifically think about using the correct arm position during at least 25% of the level transfers I perform.

Over the next week, I will specifically think about the position of my wheelchair/scooter when reaching for objects outside my base of support at least 25% of the time.

Goal:

Session #3 To Do's

Please complete the following items in the week between Session #2 and Session #3.

- Respond to the journal question (Page 55) regarding modifications to your environment and action planning.
- Complete one action plan (blank sheets are available in your folder).
- Complete the "Fall proofing your home and environment" checklist on pages nine through 16 of the minimizing Your Risk of Falls Brochure based on your review of your home.
- Continue doing the home exercise program three times a week (Page 16).
- Use your exercise log to track your exercises (folder).
- Set a goal regarding practicing transfer skills/practice the skills (Page 69).

How to Fall[1]

GET WISE Section: Environment

Learning "how" to fall is a very important part of managing the secondary problems associated with falls. While the goal of this program is to prevent falls from happening, some falls will still occur. Learning how to manage a fall when it does occur is important to minimize injury.

Using the following techniques will help you to decrease injuries that might occur during a fall:

Name of video: How to fall

- **Tucking your head**: This will help prevent you from hitting your head on the ground or reducing the force of the hit if one occurs. Hitting your head on the ground can cause serious brain injuries or concussions.

- **Turn your head** so that your knees don't hit your face upon impact.

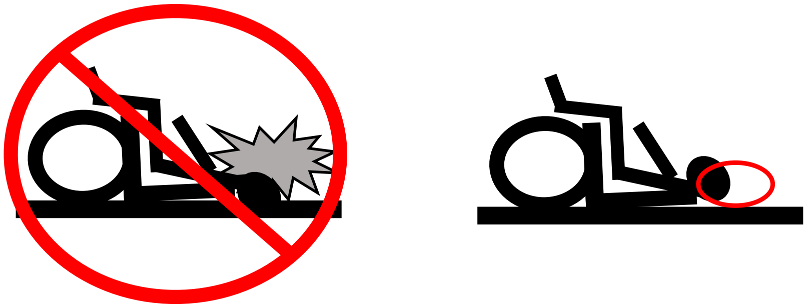

Figure 1. Tucking the head

Manual wheelchair users: If you start to fall backwards and cannot stop the movement, tuck your head and grab the wheels. This will force the chair's push handles or backrest to take the brunt of the fall and protect your head. A rigid (hard) backrest will give you more protection compared to a sling/upholstered back. Also, grabbing the wheels can help slow the descent (see Figure 2).

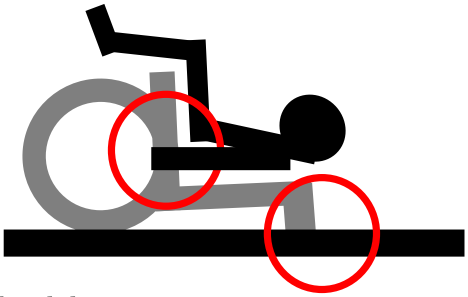

Figure 2. Manual wheelchair

Power wheelchair users: If you start to fall backwards and cannot stop the movement, tuck your head, turn your face and hold onto the arm rests. This will force the chair's push handles or backrest to take the brunt of the fall and protect your head (see Figure 3).

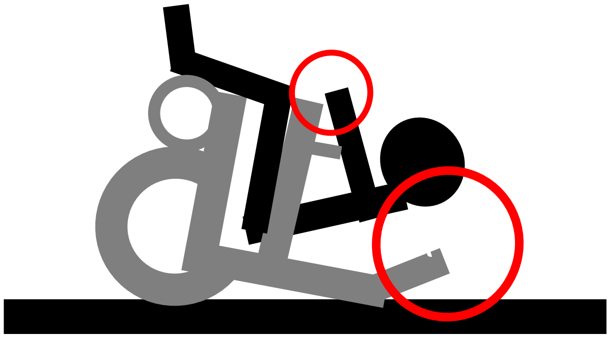

Figure 3. Power wheelchair

See the video **'How to fall'** on the study website to visualize the techniques to safely fall on the floor or the ground.

Please note: The safe fall techniques demonstrated above are difficult and require a lot of practice. If you feel that you would like to work on these techniques after the education program is over, please talk to your physician. He/she may provide a referral to a physical therapist, who will further help you with safe falling techniques.

Reference

1. Somers MF. *Spinal cord injury: functional rehabilitation.* Prentice Hall; 2001.

Fall Recovery Plan

Get Wise Section: Environment

The period of time after a fall occurs can be frightening and a period of uncertainty. Today, we will discuss different ways to help you make your own fall recovery plan and get up after a fall occurs. Developing a fall recovery plan in advance of a fall event can help to take some of the uncertainty away and increase your confidence in recovery.

Points of discussion:

- Can you get back to your wheelchair by yourself or do you need assistance?
- In general, how long does it take you to get up from a fall?
- Is there anything else that influences your ability to get back into your chair?

Why is developing a fall recovery plan important?

- After a fall, remaining on the floor or the ground for over 10 minutes after falling is common in individuals with MS.[1]
- Most wheelchair/scooter users need assistances to get up after a fall occurs.[2]
- Lying on the floor or the ground for an extended period of time is associated with additional physical injuries, a decline in performance of daily activities, an increased chance of a hospital admission and increased fear of falling[3,4].

To develop a fall recovery plan, here are a few tips:

- **Create a "check in" system** with a close friend or family member to make sure that someone is aware of what you are doing and can summon assistance quickly if a fall occurs. Pick a friend or family member that you can trust and develop a plan to call or text him/her on a daily basis at roughly the same time each day. You do not need to make a long phone call, just check in with the person so that he/she knows that you are doing okay. If you have a history of multiple falls, you might want to develop a plan for more frequent check-ins.

- **If you have a smartphone, set up the emergency features**. Many modern smartphones have emergency features that can help notify family or friends if you need assistance recovering from a fall.
  - For **newer iPhones**, press and hold the side button and one of the volume buttons until an "Emergency SOS" slider appears.

  - For **older iPhones**, rapidly press the side button five times. By swiping the "Emergency SOS" slider, local emergency services will be called and your emergency contacts (which you will need to pre-set in your Health app) will automatically be sent a text message with your current location, even if your location feature is off in your general settings.

- For **Samsung Galaxy phones**, you will need to set up "Emergency Messages" prior to this function being accessible. To do this, go to "Settings" and click on "Advanced features". In this menu, click "Send SOS messages" and "agree" to the terms of use. It will prompt you to add contacts to receive your SOS message. Once a contact is added, you can press the power button quickly 3 times in an emergency situation. This will automatically send an alert to your designated emergency contacts.

- For further details on how to set up your specific phone's emergency features, go to your phone provider's website for more information:
  - i-Phone: https://support.apple.com/en-us/HT208076
  - Samsung Galaxy: http://www.samsung.com/nz/support/mobile-devices/samsung-sos-smart-phone-emergency-message-guide/

➢ **Stay connected.** Below is a listing of strategies to make sure that you can always get help if you fall.

- **Consider using a medical alert device.** A medical alert device is a wireless device that can either be automatically or manually triggered to call for assistance when a fall occurs. The device can be worn around your neck or on your wrist to assure that it is always with you. These devices can be expensive, so make sure you shop around before selecting a device. (Please see page 75 for a listing of medical alert systems).

- **Use of a wearable communication device**. Many companies are developing "smart" watches or devices that allow a person to wear a device that allows them to connect to their cellular telephone. These devices can help you call for help if you get separated from your phone. These devices however can be expensive and provide more functionality than is needed to simply communicate.

- **Low tech**: If you would prefer not to use a medical alert system or a wearable communication device, you can develop your own low tech solution to assure you are always connected. You can put a cellular telephone in a waist ("fanny") pack or a small pouch around your neck. When selecting a bag, make sure that it does not affect your ability to transfer, push or drive your power wheelchair/scooter, or perform any other activities of daily living.

➢ **Develop a plan to get off the floor.** It is very important that you have a plan in place to be able to get up off the floor after a fall occurs. Please see the details in following section "Fall Recovery Strategies".

A list of medical alert system		
**Company**	**Device Features**	**Contact Information**
MEDICAL GUARDIAN	Wearable button which connects to landline or Bluetooth enabled phone for easy communication Sensors offered to track activity	Phone<br><br>(888)983-4735<br><br>Website<br>MedicalGuardian.com
MedicalAlert™ Help at the Push of a Button	24/7, 365 day monitoring<br><br>Up to 32 hours of backup battery<br><br>Operators stay on the line until help arrives	Phone<br><br>(888)884-5285<br><br>Website<br>MedicalAlert.com
LifeStation®	Fall detection sensors available<br><br>Mobile GPS option for on-the-go	Phone<br><br>(888)983-1746<br><br>Website<br>LifeStation.com
MobileHelp	Works at home and on-the-go with GPS<br><br>30 hour backup battery<br><br>Water resistant and waterproof devices available	Phone<br><br>(888)473-8297<br><br>Website<br>MobileHelp.com
helpbutton.com	All devices have unlimited talk and call time<br><br>Ally Location option available<br><br>Fall Detection available	Phone<br><br>(855)808-9545<br><br>Website<br>HelpButton.com

ADT HEALTH	Press button on necklace or bracelet and receive help<br><br>Smaller devices for on-the-go are available	Phone<br><br>(866)419-3254<br><br>Website<br>ADTMedicalAlert.com
greatcall.	Cellular phones available for easy communication and device pairing<br><br>Lively Wearable connects to cell phone<br><br>Instant Fall Detection in all devices	Phone<br><br>(888)566-8769<br><br>Website<br>GreatCall.com
LifeFone.<br>MEDICAL ALERT SYSTEM	Fall Detection options available for all devices<br><br>Up to 30 hours of backup battery life<br><br>Includes customized care plan<br>Waterproof Pendant	Phone<br><br>(888)983-2363<br><br>Website<br>LifeFone.com
PHILIPS Lifeline	Select devices include Fall Detection<br><br>Wearable pendant	Phone<br><br>(855)681-5351<br><br>Website<br>LifeLine.Philips.com
QMedic	Alerts when users are more sedentary than usual<br><br>Tracks activity - can be sent to family<br><br>Waterproof<br><br>Active and proactive alerts	Phone<br><br>(877)241-2244<br><br>Website<br>QMedicHealth.com

Note: For informational purposes only. DPQoL has no relation to any of the devices listed.

Fall Recovery Strategies

This section covers different strategies to use to get yourself off of the floor following a fall. Several options are outlined below. However, depending on your specific functional abilities, you may need to modify these suggestions. Please talk with your trainer about your specific needs.

When a fall occurs, please follow these steps:

- Take a deep breath and orient yourself to the situation. Don't try to get up quickly.

- Evaluate the situation to determine if you are seriously injured. If you think you might have a broken bone or have a head injury, please call 911 or ask a bystander to call for you. As much as possible, please try to stay still to prevent further injuries.

- If you are not seriously injured, and you are able to transfer back to your wheelchair/scooter with/without assistance, please perform a floor to chair transfer (Instructions provided below).

- Once you are sitting back in your seat, take a moment to make sure you are correctly positioned in your chair. Take a deep breath before going back to your normal daily activity.

- See your doctor if you are concerned about any injuries. Please see the section 'medical attention after a fall' on page 82 to help you decide if you should call your doctor. **If you hit your head, you must call your doctor.**

Floor to Chair Transfer Skills (Weight Bearing)

Please see the videos **'Floor to Chair Transfers'** on the study website to visualize the techniques to effectively perform a floor to chair transfer.

Name of Video:
- **Floor to Chair Transfers without assistance**
- **Floor to Chair Transfers with assistance**

Floor to chair transfer without assistance (weight bearing)

1) Roll yourself onto your stronger side (if applicable) and place both of your hands on the floor in front of you.

2) Push your torso up by continuing to roll until your chest is facing the floor with your hands in position under you. Shift your weight off of your side and into your hands and knees so that you are in a crawling position.

3) Crawl over to a stable surface, such as a chair, and pull yourself up into a kneeing position by resting your upper body on one of your forearms on the surface and pushing off with the arm remaining on the floor.

4) Once both of your hands are stable on the surface, shift your weight to one knee on the floor, so that you may bring the opposite knee up to a 90 degree angle, planting your foot on the floor.

5) Lean forward onto your hands, pushing down through the foot on the floor. Press your body weight up on one leg until you are able to bring the other leg into a standing position.

6) Shift your hands to the side of the seat. Turn your body and sit on the seat, bed, or flat surface. Alternatively, you can bring your leg under you and continue to a full stand up position.

Floor to chair transfer with assistance (weight bearing)

To help you up from the floor, your care partner will assist you into a crawling position, then a standing position.

1) If you are lying on the floor on your back, have the care partner help you bend your knees up so both of your feet are resting on the floor.

2) Next, have your care partner position your legs to lean across your body in the direction you want to roll. They will help you roll over onto your preferred side. Have your care partner help by gently pushing at your hips and shoulders.

3) Ask your care partner to help you bring the top leg out in front of the bottom leg.

4) Have your care partner place his/her hand under your shoulder that is touching the floor and lift so you can pull out the arm and rest your weight on your hand instead.

5) Have the care partner move their hands to your hips and help lift you up and over, allowing you to get on your hands and knees in a crawling position.

6) Have your care partner assist you in crawling to your wheelchair/scooter or a sturdy secure piece of furniture and place your hands on it. Make sure the wheelchair/scooter is locked if you transfer to the wheelchair/scooter.

7) Pull your knee forward and place your foot directly under the knee. This should be done with the leg you feel is the strongest. Your care partner can assist you with this if needed.

8) Have your care partner come around to the other side of you and help lift you to a standing position by hooking one arm under your arm and placing other hand on your back.

9) When you stand, take a moment to make sure you are steady and slowly turn and sit with assistance. Make sure that your care partner holds you for your balance while turning and sitting.

Please Note: The information above is for someone who can bear weight on their legs. If you cannot weight bear, please see the video 'Floor to Chair Transfers, Non -Weight Bearing'' on the study website to visualize the techniques to effectively perform a floor to chair transfer. You will find three videos that demonstrate three different types of floor to chair transfer techniques: front approach, back approach, and side approach. Please choose one technique that you think you could perform most efficiently. The floor transfer techniques are difficult and require a lot of practice. If you feel that you would like to work on these techniques after the education program is over, please talk to your physician. He/she may provide a referral to a physical therapist, who will further help you with safe fall recovery techniques.

Name of Video:

- Floor to Chair Transfers, Non -Weight Bearing (Front approach)
- Floor to Chair Transfers, Non -Weight Bearing (Back approach)
- Floor to Chair Transfers, Non -Weight Bearing (Side approach)

Two-person total assist floor to chair transfer (Non-Weight Bearing)

If you need total assistance to perform a floor to chair transfer, you should develop a plan with your care partner ahead of time. Make sure to have your care partner practice the steps either with you or a friend/family member who can easily get on/off the floor. You should also practice providing instructions to a bystander incase a fall occurs and one of these trained individuals cannot assist you.

The following steps below demonstrate how two people can assist you to transfer from a floor to the chair. If you need total assistance to perform a floor to chair transfer, having two people help you is necessary for both your safety and the safety of your care partners.

1) Have your care partners communicate with you throughout the whole transfer process. Make sure to have them explain what they are doing and going to do before taking action. For example, the care partner can provide you instructions such as "On the count of three, I will lift you up and help you move to this chair."

2) Ask your care partners to position your wheelchair/scooter as close to you as possible and to lock the brakes or turn off the power.

3) Have your care partners remove the arm rest or any other parts of your wheelchair/scooter that could interfere with the transfer prior to attempting the transfer.

4) If you are lying on the floor, ask your care partners to assist you into an upright position.

5) Have one person position themselves behind you. Then, ask the person behind you to place his/her arms under your armpits and grasp her/his own wrist firmly while you cross your arms on your chest.

6) Have the other care partner position themselves in front of you and ask the front person to lower his/her body to grab your lower extremities. Have the care partner place both arms under your knees and grasp his/her own wrists for a strong hold.

7) When they are ready, ask them to lift you up together on the count of three. Have care partners use a squat technique when they lift you up. They need to bend with their knees, not with their back.

8) Have your care partners walk to the wheelchair/scooter and lower you into the wheelchair/scooter using a squat technique.

9) Ask them to make sure you are secured and stable.

10) Teach your care partners to use assistive devices if appropriate (e.g. gait belt, transfer board, draw sheet, Hoyer lift).

Remember! It is important that you give people clear instructions on how you need to be assisted. Do not let them help you without a plan. Sometimes, improper transfer assistance can aggravate an injury sustained in a fall or otherwise.

Assistive Technology

To make the floor to chair transfer a little easier, you might want to consider the use of assistive technology. Assistive Technology can play an important role in facilitating the performance of a floor to chair transfer. Here are some options that may be beneficial to you:

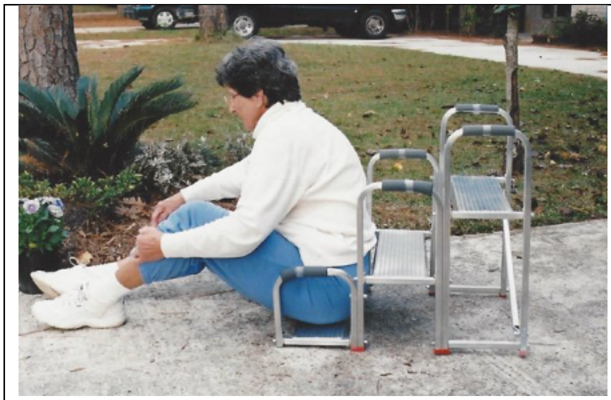	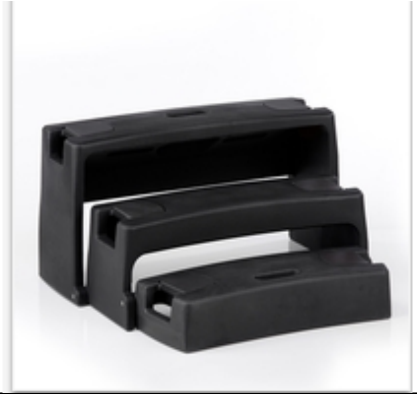
Para Ladder: (price range:$950- $1100)	ResQup: (Price range $350-$400)

Note: DPQoL has no relation to any of the devices listed.

These devices are essentially a series of steps that help to create intermediate surfaces between the floor and your wheelchair/scooter.

- Sit on the bottom seat.
- Push yourself up to reach the next seat.
- Have at least one care giver assist or watch you while using the device.
- Do not use this device if you experience any problems or discomfort.

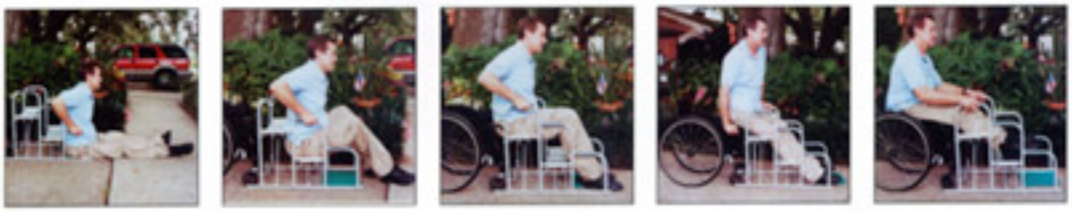

Use of the para ladder to perform a floor to chair transfer.

Medical Attention After a Fall

After a fall occurs it is important to get the appropriate medical attention. Taking the time to speak with your doctor or a medical professional after a fall will help to prevent chronic, longstanding injuries that may have a long term, negative effect on your health and well-being. It is critically important that you call your doctor if you experience a fall where you:

- Hit your head
    - In addition to calling a doctor, you should be monitored closely for signs of a concussion, even if you feel okay. An undiagnosed concussion can put you at risk for brain damage. Some important symptoms of concussion to be aware of include:
        - Loss of consciousness
        - Severe headache, including a headache that gets worse
        - Confusion and saying things that don't make sense
        - Slurred speech
        - Unresponsiveness

- Sustain an injury
- Have significant pain for more than 24 hours

It is also very important to speak with your doctor if you have experienced an increase in your frequency of falls or experience a fall after a recent medication change.

Putting it all together

Let's take a look at a comprehensive fall management plan:

Before a fall occurs:

- Consider:
    - If I fall, who should I contact?
    - How will I contact this person? (Medical Alert Device vs. Wearable Communication)
    - If I fall and no one is available, what would I do?
- Develop a check in plan.
- Practice floor to chair transfer skills with a few care partners.

If a fall occurs:

- Don't panic and take a deep breath. Don't try to get up quickly.

- Evaluate the situation to determine if a serious injury has occurred.
    - If, yes, call 911 first and then family members. Stay as still as possible to prevent making injuries worse.

        - If no, evaluate the situation and figure out what is needed to help you get back up.

- Determine if you are able to get up by yourself.
    - If, yes, perform floor to chair transfer to get back into your chair.
    - If no, ask for help using your established strategy.

- If you are waiting for assistance and not injured, attempt to get into a comfortable position and conserve energy until someone who can help you arrives. If you have limited sensation, make sure you are not putting excessive pressure on any areas.

- Once help has arrives, use your established recovery plan.
    - What are you going to say to helpers assisting you to get back up?

After getting back up to your chair:

- Re-evaluate any injuries and determine if you should call your doctor. **If you hit your head, call your doctor.** If you don't feel you are injured, monitor your symptoms.
- Think about if your fall recovery plan worked as you expected.
    - Do you need to modify anything?

- Think about why you fell.
    - Could the fall have been avoided?
    - Are there any skills you can work on?

References:

1. Bisson EJ, Peterson EW, Finlayson M. Delayed initial recovery and long lie after a fall among middle-aged and older people with multiple sclerosis. *Archives of physical medicine and rehabilitation.* 2015;96(8):1499-1505.
2. Rice LA, Sung J, Peters J, Bartlo WD, Sosnoff JJ. Perceptions of fall circumstances, injuries and recovery techniques among power wheelchair users: a qualitative study. *Clinical rehabilitation.* 2018:269215518768385.
3. Bloch F. Critical falls: why remaining on the ground after a fall can be dangerous, whatever the fall. *Journal of the American Geriatrics Society.* 2012;60(7):1375-1376.
4. Tinetti ME, Liu WL, Claus EB. Predictors and prognosis of inability to get up after falls among elderly persons. *JAMA : the journal of the American Medical Association.* 1993;269(1):65-70.

Session #3 Journal Entry

What will be done:

You will be asked to make a journal entry every week after completion of the session. Your journal entry will be tied to items discussed during the previous session. You will typically be asked to respond to a specific question regarding the content of the program from the previous session.

Why:

The journal entries will help you to think about the information that was presented during the education session and to help you integrate what you have learned into your everyday life.

Prompt:

Please think about your worst fall in the past year. What happened after the fall occurred? Please specifically think about how you got up from the fall, how long you were on the ground and if any assistance was needed. Taking into consideration this information, what aspect(s) of your fall management plan do you think will be most important to develop or revise?

Response:

<u>Fall Management Worksheet</u>

Now that you have learned about various strategies to minimize the negative consequences associated with falling, it is time to work on developing a <u>*specific plan*</u> so that if a fall occurs, you will be prepared.

Before a fall occurs:

- <u>Development of a "check in" system.</u>

**Who will you check in with at least once per day?**	**Name:**	**Phone number/e-mail address:**	**Method of communication** (circle all that apply):<br><br>Call:<br>Text:<br>E-mail:<br>Other, please describe:

When will you talk to the person named above to set up the check in plan?

**Date:**	**Time:**	**How will you remind yourself to do this?** (please circle all that apply)<br><br>Set reminder in phone<br>Write on calendar<br>Other, please describe:

- <u>Select a communication device.</u>

When a fall occurs, it is critically important that you are able to communicate with family and/or friends if you need assistance.

**What type of communication device will be your <u>primary</u> way to communicate?**	**Please circle one:**<br><br>Cell Phone<br><br>Medical Alert System<br><br>Unsure at the current time – preliminary ideas:

Please answer the following questions about your primary communication device:

**When will you investigate what type of technology you want to use?**	Date:<br>Time:<br>How will you remind yourself to do this?<br>• Set reminder in phone<br>• Write on calendar<br>• Other, please describe:
**Once you have decided on the technology, please make a list of tasks that will need to be done.**<br>We have started the list of common items. Please circle all that apply and add additional items:	**To-Do:**<br>• Set up emergency features on SmartPhone<br>• Order a medical alert device<br>• Determine a method of wear or carry your communication device<br>• Talk with care partner about your communication device<br>• Other items:<br>•<br>•<br>•<br>•<br>•<br>•<br>•
**How will you wear the device or make sure that it is with you at all times (including night time)?**	Please describe in detail:

- Practice fall recovery.

To reduce stress after a fall occurs, identifying who will assist you, if necessary. Practicing how you will get off the floor also is helpful. To avoid unnecessary injury, talking through the plan with your care partner and then practicing the movements with another family member/friend who can easily get off the floor is best. Please do not go down to the floor unnecessarily!

**Who will help you recover in the event of a fall?**	Primary person:<br>• Name:<br>• Phone number:<br>Backup #1:<br>• Name:<br>• Phone number:<br>Backup #2:<br>• Name:<br>• Phone number:
**What fall recovery strategy do you think is best for you?**	Please circle what you think is best:<br>• Independent floor to chair, non-weight bearing*<br>• Independent floor to chair, weight bearing*<br>• Assisted floor to chair

*If you chose any of the independent transfers, make sure to also practice an assisted transfer as you could become injured during a fall and may need assistance.

**How will you practice fall recovery?**	Please circle all that apply and add additional information:<br>• Watch videos on iROLL website<br>• Discuss techniques with care partner<br>• Buy assistive technology that can help with recovery.<br>o What assistive technology will you buy? Please describe:<br>• Practice recovery plan with care partners<br>• Other, please describe in detail:

**When will you practice fall recovery?**	Date:<br>Time:<br>How will you remind yourself to do this?<br>• Set reminder in phone<br>• Write on calendar<br>• Other, please describe:

When a fall occurs:

If you make the appropriate plans prior to a fall, all you will need to do if a fall occurs is execute your plan. While a fall will most likely always have stress associated with it, planning helps to take some of the stress away.

Complex Transfers

GET WISE Section: Transfers

During the previous session you learned basic transfer skills. Please feel free to refer back to page 35 (independent)/39 (assisted) of the education material to review these skills as often as necessary. Today, we will discuss how to perform more complex transfers making use of the basic skills you learned.

We will focus today on performing transfers to and from the toilet, a vehicle, and a bed. These are some of the most common transfers you will encounter. In addition, being able to perform these transfers will help you to navigate not only your own home but increase your confidence in your ability to perform transfers necessary to participate in activities you enjoy doing.

Today, we will help you learn the fundamental skills of these transfers. However, if you would like more practice or have a particularly difficult environment in which these transfers must be performed, you might want more practice time. Please tell your doctor you are having difficulty performing these essential transfer skills and ask for him/her to order Physical or Occupational Therapy services.

If you need assistance to transfer, please refer to the basic assisted transfer education materials on page 39 of the manual. You can use the same general techniques described in this section while performing complex transfers. During the practice sessions, you can ask your trainer specific questions on how assistance can be provided in your unique environment.

Point of discussion:

- What are some of the most difficult transfers you have encountered?
- Are any of your desired activities limited due to trouble performing complex transfers?

Toilet Transfers

Setting up your environment:

Toilet transfers are often difficult due to space limitations. Because toilet transfers are performed frequently, modify your bathroom at home, if possible, to give you enough space to perform a transfer using your preferred method. The US Access Board has detailed information on ideal bathroom set ups at www.access-board.gov. If you are doing major reconstruction, it is best to work with a contractor who has experience modifying homes for wheelchair or scooter users. You should also take some time to determine if you have any unique space requirements above and beyond the standard guidelines.

Any transfer in the bathroom (toilet, shower) can become more dangerous due to wet and slippery surfaces. Please take care to make sure that your shower has a good drainage system that prevents water from pooling on the floor. If you notice water on the floor, please wipe it up as soon as possible or ask your care partner to assist you. Even a little bit of water can be very dangerous!

When you are away from your home, transfers can be more challenging because the area hasn't been set up specifically for you. After reviewing the tips for success, your trainer can help you problem solve through unique challenges you might face.

Tips for success

- Seat height:
    - Investigate assistive technology that allows for performance of a level transfer.
    - Take measurements of your chair and compare to the toilet. Items such as elevated toilet seats or padded seats can help make the transfer level.
    - Measure before you buy! Standard toilet seats are often fairly level with wheelchairs for people performing sitting pivot transfers. However, if you perform a stand pivot transfer, an elevated seat will be very helpful.

- Grab bars:
    - Grab bars can make the transfer easier and provide a good handgrip.
    - Caution: if the grab bar is in the wrong place, it might make things worse. You should not have to lean excessively forward or to the side to grab the bar. The bar should be close to where your hand naturally falls when you transfer.
    - Use of grab bars that can be adjusted to your needs and lowered down next to the toilet allows you to modify the environment to your specific needs.

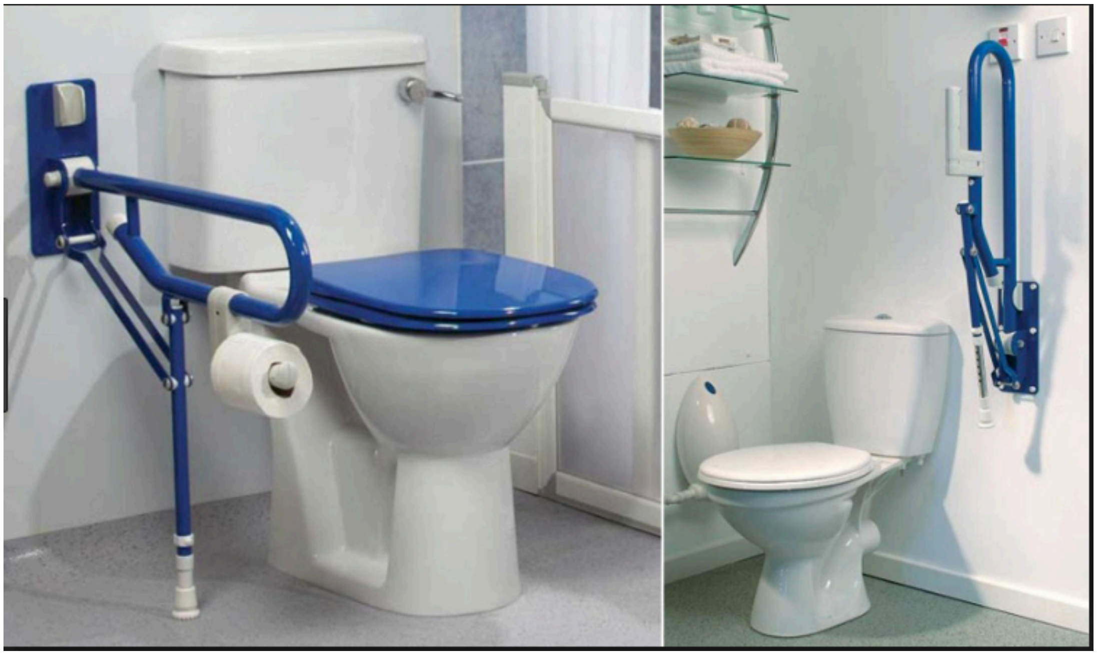

Note: **<u>DO NOT</u>** use towel bars as a grab bar. Towel bars cannot support more than about five pounds and will break if you put your weight on the device. This could result in a serious fall with substantial injuries. If you choose to use a grab bar on the wall, it should be installed by a professional contractor familiar with the process. For example, they have to be secured to the studs within the walls or will pull out from the wall at first stress.

- Clothing management:
    - Depending on your specific toilet routine, clothing management can add an additional challenge to toilet transfers.
    - If you pull down your pants, make sure that you are on a stable surface that you can safely shift your weight.
    - If you perform the transfer after pulling down your pants, make sure that your pants are positioned so that you have freedom of movement and won't get caught on your wheelchair or the toilet.
    - If clothing is difficult to reach from the floor following bathroom usage, you can consider a reaching aide (e.g. reacher or dressing stick) placed in close proximity to the toilet.

Name of video: Bathroom Transfers
Start at 3:38
End at 5:09

Performing the transfer

You can use many of the same techniques learned during Session #2 (Page 35/39), especially if you are able to set up the transfer to be level. Generic suggestions are provided

below for a sitting pivot transfer. Please talk with the trainer if you have questions about your own environment.

Key points:

- Approach the toilet by angling your wheelchair/scooter so that the front corner of your chair is next to the front of the toilet. If you can't get beside the toilet, try to get as close as possible.
- If possible, remove the armrest and/or sideguard that is closest to the toilet.
- Lock your brakes.
- Put your feet on the floor.
- Scoot forward.
- Place one hand on the far side of the toilet seat. Place the other hand on your cushion/armrest/wheelchair frame as described during Session #2. Don't forget to use the correct handgrip!
- Once you feel the set-up is stable, use the head-hips relationship to pivot yourself onto the toilet.
- If the transfer is uneven, the head-hips relationship can help you to get additional upward lift.

If you perform a standing pivot transfer to the toilet:

- Many of the same techniques will apply, however you may need additional space depending if you take a step during your pivot transfer.
- If you don't have a raised toilet seat, grab bars can be very helpful.
- Make sure you either feel the toilet on the back of your legs (or see that you are in close proximity) before lowering down.

<u>Vehicle Transfers</u>

Performing a transfer to/from a vehicle can be one of the most challenging transfers to perform but is very important to allow you to participate in your community. Because there are so many different vehicle types, the specific techniques will vary. However, we will provide general tips on methods to make the transfer safe and easy. If you would like additional, individual practice, please ask your doctor to order Occupational Therapy services for you.

Setting up your environment:

Vehicle transfers are challenging due to space limitations, a large gap between your chair and the seat and height changes. When possible, attempt to perform a level transfer into your vehicle. If you are able to buy a new vehicle, you should consider the following items:

- Modified vehicle: The safest option is to buy a vehicle with a ramp in which you can push/drive your chair into the vehicle. You can either stay in your chair (either to

drive or ride as a passenger) or transfer to a seat in the vehicle. Please talk to your wheelchair/scooter vendor about resources to modify a vehicle. The National Disability Institute provides low interest loans for modified vehicles or other assistive technology. Details can be found at the following website:

https://www.realeconomicimpact.org/assets/site_18/files/alternative%20financing%20programs%202-2-18.pdf

- In general, larger vehicles, lower to the ground make transfers easier. Transferring into a sports utility vehicle (SUV) or truck can be very challenging and put you at a high risk for falling and upper limb impairments. Station Wagons or crossover utility vehicles can give you the needed space but are also low to the ground. Mini-vans also work fairly well.

Tips for success:

- Create a solid base of support. Consider different foot placements and hand holds to create a solid foundation to perform your transfer to/from. Using the car seat or dashboard are good options. You might have to try out a few different options to figure out what is best.

- Consider how you will load your chair into your vehicle. Working with a therapist can help you to problem solve through a different method to load your chair independently to reduce your dependence on a care partner.

Name of video: Car Transfers
Start: 1:45
End: 5:22

Performing the transfer:

Key points:

- Angle your chair to get as close to the vehicle as possible.
- Move the seat back as far as possible to give you more leg room. You may also want to recline the seat to open up the space.
- Align the backrest of your wheelchair with the back of the vehicle seat.
- Lock your brakes.
- Scoot forward in your chair.
- Place your feet in a position that will be stable through the course of the transfer. You may need to put one foot on the ground and one in the vehicle so that you can maintain a solid foundation. You may have to try this a few times to figure out the optimal placement.
- Place your hands on a solid surface that will not move during the course of the transfer. Avoid using the car door as a support surface.
- Using the head-hips relationship, pivot yourself into the vehicle.

If you perform a stand pivot transfer:

- The same principles will apply. However, you will leave both feet outside of the car during the transfer.
- The dashboard and back of the seat are good locations to place your hands to slow your descent.

Note: The MS society has a nice resource for vehicle modification needs:

https://www.nationalmssociety.org/nationalmssociety/media/msnationalfiles/brochures/brochure-driving-with-multiple-sclerosis.pdf

<u>Bed Transfers:</u>

Transferring from your bed to and from your chair is a critical skill that will be performed multiple times on a daily basis. Many of the same techniques learned during the basic transfer skills session (Page 35/39) will be applied here.

Setting up your environment:

- When possible, give yourself enough space to allow you to position your wheelchair/scooter in your desired position.
- Attempt to set up your bed so that you can perform a level transfer. You may need to remove part of the bed frame or simply put your box spring and mattress directly on the floor. Platform beds can also be purchased which are often low to the ground.

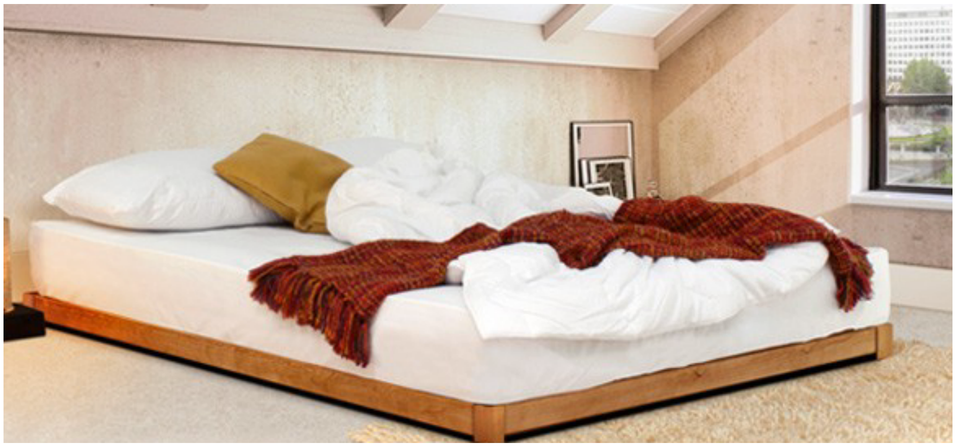

- Chose a mattress that will provide sufficient support and pressure management (if necessary) but isn't so soft that it makes the transfer difficult. You might want to go to a store and feel the mattress yourself to help make a decision.
- Consider the use of a motion activated light so that if you need to use the bathroom in the middle of the night you can easily turn on a light and won't need to reach for a light switch.

- If you need to use the bathroom frequently, you might want to consider the use of a bedside commode that can sit next to your bed. For males, a urinal can also be a convenient option.
- Make sure that you have enough space to park your wheelchair or scooter next to your bed. Prior to performing the transfer, make sure the brakes are locked/power turned off to enhance safety during your transfer and the device is ready to go when you get out of bed.
- You may also want to consider the use of a grab bar that can be attached to the edge of your bed. One can be purchased from rehabmart.com.

Tips for success:

- Take care when getting out of bed immediately after waking. People are often disoriented and unsteady if they quickly move from a lying to sitting position. After sitting up, take a moment to orient yourself before transferring into your wheelchair/scooter.

Name of video: Bed Transfer
Start: 1:09
End: 3:25

Performing the transfer:

Many of the same techniques described in the "basic transfer skills section" are used here. Please refer back to the more detailed instructions on page (Page 35/39).

- Set up your wheelchair/scooter at about a 45 degree angle to the bed.
- Lock your brakes.
- Scoot forward.
- Put feet on the floor.
- Place one hand on the bed and the other on a secure part of your wheelchair/scooter. Remember to use a stable handgrip!
- Use the head-hips relationship to help you pivot onto the bed.
- When you land, remember that the surface will be soft. Be careful when repositioning.

If you perform a stand pivot transfer:

- Many of the same principles will apply.
- Before sitting, make sure you can feel the bed on the back of your legs. If you have limited sensation, look to make sure the bed is behind you.

Complex Transfer Discussion

Now that we have learned the "ideal" way to perform complex transfers, let's take a minute to discuss how you are performing these transfers.

- How do you typically perform a transfer to the toilet, a vehicle, and your bed?
- What part of the transfer is most difficult for you?
- How is your transfer different from the "ideal" method?

Session #3 Goals

During today's session, complex transfer to a toilet, vehicle and bed were discussed. Please write a goal, using the SMART goal framework, describing how you will practice the skills learned today.

S	Specific.
M	Measurable.
A	Achievable.
R	Relevant.
T	Time bound.

Example: *I will use the seat elevator on my power wheelchair/scooter to perform a downhill transfer to my bed during at least 50% of the transfers over the next week.*

Goal:

Manual Wheelchair Skills Education (Intermediate)

Get WISE Section: Wheelchair Management

Today, we will learn and review intermediate wheelchair skills to allow you to safely and successfully navigate a variety of environments in your home and community.

Pushing on a soft surface

There are many soft surfaces (e.g. carpet, dirt, grass, gravel, or sand) with increased rolling resistance. Pushing your wheelchair is more difficult on such surfaces because the wheels tend to sink into the surface.

Name of video:
Pushing on a carpet (no pop-up)
Pushing on gravel (using pop up techniques)

General tips to reduce the frequency of falls and injury:

- When approaching a soft surface, look ahead and plan a route that will minimize the amount of time you must push on a soft surface.
- When pushing from a smooth level surface onto a soft surface, be aware that your wheelchair can suddenly slow down or stop because of the increased rolling resistance. It is a good idea to slow down when approaching a soft surface for a smooth transition.
- Continue to use good propulsion techniques (e.g. use long, smooth strokes)(see page 58). You will need to use more force due to the increased rolling resistance on soft surfaces.
- Only move in a forward direction when pushing over soft surfaces so that you can see any unexpected hazards in your path.
- Keep as much weight as possible over the rear wheels without leaning backwards. This technique prevents the front casters from digging into the soft surface.
- If possible, perform a wheelie to lift the front casters off the ground. If this is not possible, consider popping up the front wheels(casters) off the surface before each push. This keeps the front wheels (casters) free from sinking or dragging into the soft surface. Lift the casters off the surface during each push, letting them touch the surface as the hands recover for the next push. Please review the points below to perform a wheelchair pop-up.

Name of video: Wheelie pop-up

General tips to reduce the frequency of falls and injury:

- A wheelchair pop-up involves lifting your front wheels(casters) off the ground briefly.
- Push the wheelchair forward to do a pop-up by positioning your hands on the upper back of your hand rims and applying a rapid short push forward to lift your front wheels (casters) off the ground. Then allow your front wheels (casters) to return to the ground.

NOTE: Make sure you have a spotter with you when learning and practicing this task. Until you are able to perform the skill correctly consistently, there is a high potential that you could fall.

Getting over an obstacle

You will often encounter obstacles (e.g. door thresholds) that you may not be able to simply roll over. Alternative strategies may be needed. For example, you might need to pop the casters over the obstacle.

Name of video:
Getting over an obstacle

General tips to reduce the frequency of falls and injury:

- Search for an alternate route when you approach obstacles. The best method is to avoid them, steering around them or straddling them if possible.
- Approach an obstacle at a slow speed. It is easier to pop the front wheels (casters) when moving.
- Don't lean forward when you approach the obstacle, it can increase the weight on the front wheels (casters) and make it difficult to pop the casters.
- Approach an obstacle straight forward. Approaching the obstacle to the side can make it more difficult to get over the obstacle.
- Pop the front wheels (casters) up off the floor once you are close to the obstacle you wish to overtake. Be sure to lift the front wheels (casters) just high enough to clear the obstacle.
- Use both of your hands to propel yourself forward, applying forward forces of moderate intensity to the hand-rims to pop the front wheels (casters).
- Lean forward slightly when the casters have landed beyond the obstacle and the rear wheels strike the obstacle.

Getting over a gap

A gap in surface support is a common barrier (e.g. potholes on the road). A gap can cause a wheel to become stuck or tip your wheelchair as you pass over, both of which can cause of falling. Even if no tip or fall occurs, it can still be challenging to get your wheelchair out of a gap if a wheel becomes stuck. Therefore, it is important to learn the proper technique to avoid these situations.

Name of video:
Getting over a gap

General tips to reduce the frequency of falls and injury:

- Search for an alternate route when you approach gaps. The best method is to avoid them, steering around them or straddling them, if possible.
- Use the same techniques that you use when getting over an obstacle to get over a gap (See the "getting over an obstacle" skill).
- If the front wheels (casters) drop into the gap and turn sideways (a common problem if the wheelchair is moved forwards and backwards repeatedly in an attempt to get the casters out of the gap), it can be very difficult or impossible to proceed without assistance.
- Consider using the full wheelie technique to cross over a larger gap (if applicable).

<u>Power Wheelchair and Scooter Skills Education (Intermediate)</u>

Get WISE Section: Wheelchair Management

Today, we will learn and review intermediate wheelchair/scooter skills to allow you to safely and successfully navigate a variety of environments in your home and community.

<u>Driving on a soft surface</u>

There are many soft surfaces (e.g. carpet, dirt, grass, gravel, or sand) with increased rolling resistance. Driving your wheelchair/scooter is more difficult on such surfaces because the wheels tend to sink into the surface.

<u>Name of video:</u> Power Wheelchair Skills
Start: 9:25
End: 9:38

General tips to reduce the frequency of falls and injury:

- When approaching a soft surface, look ahead and plan a route that will minimize the amount of time you must drive on a soft surface.
- When driving from a smooth level surface onto a soft surface, be aware that your wheelchair can suddenly slow down or stop because of the increased rolling resistance. It is a good idea to slow down when approaching a soft surface for a smooth transition.
- If possible, select the driving option that provides more power. Sometimes a chair has an "outdoor" mode that provides more power.
- Only move in a forward direction when driving over soft surfaces so that you can see any unexpected hazards in your path.
- On soft a soft surface, drive fast enough to maintain forward movement but not so fast that you lose control.
- Be aware that your wheelchair can get stuck on a soft surface (e.g. sand, gravel, or mud). As much as possible, avoid these surfaces. If avoidance is not possible, maintaining a moderate speed can help you from getting stuck.

Scooter Considerations

- Use the same techniques as described above.
- Do your best to avoid these surfaces. Most scooters are not designed to maneuver over these surfaces.

Getting over an obstacle

You will often encounter obstacles (e.g. door thresholds) that you may not be able to simply go over.

Name of video: Power Wheelchair Skills
Start: 9:46
End: 9:52

General tips to reduce the frequency of falls and injury:

- Search for an alternate route when you approach obstacles. The best method is to avoid them, steering around them or straddling them, if possible.
- Elevate your footrests (if necessary/possible) to give you more clearance.
- Approach an obstacle at a slow speed and drive forward. Smooth continuous forward movement is the most successful method of traversing an obstacle.

Scooter Considerations

- Use the same techniques as described above
- Do your best to avoid these surfaces. Most scooters are not designed to maneuver over rough terrain.
- Very carefully consider the height of the obstacle before attempting to go over it. Scooters are very low the ground and often get caught on obstacles.

Getting over a gap

A gap in surface support is a common barrier (e.g. potholes on the road). A gap can cause a wheel to become stuck, or tip your wheelchair as you pass over, both of which can cause a fall. Even if no tip or fall occurs, it can still be challenging to get your wheelchair out of a gap if a wheel becomes stuck. Therefore, it is important to learn the proper technique to avoid these situations.

Name of video: Power Wheelchair Skills
Start: 9:40
End: 9:44

General tips to reduce the frequency of falls and injury:

- Search for an alternate route when you approach gaps. The best method is to avoid them, steering around them or straddling them, if possible.
- If avoidance is not possible, use the same techniques as described in the "getting over an obstacle" section. Maintaining a controlled, forward motion will give you the necessary momentum to transverse the obstacle.
- If the small wheels (casters) get stuck sideways in the gap (a common problem), move your wheelchair forwards and backwards repeatedly to get the casters out of the gap.
- Ask for assistance to get out of the gap if the wheelchair gets stuck and you cannot manage it.

Scooter Considerations

- Use the same techniques as described above
- When possible, avoid going over a gap in your scooter. Due to the low clearance and instability of a scooter, you are at high risk of falling.

Session #4 To Do's

Please complete the following items in the week between Session #3 and Session #4.

- Continue doing the home exercise program 3x/week (Page 16).
- Use your exercise log to track your exercises (folder).
- Work toward completion of fall management plan (Page 85).
- Goal form: Practice either a toilet, vehicle, or bed transfer (Page 97).
- Complete your journal entry (Page 84).

Influence of MS Symptoms on Falls

GET WISE Section: Symptom Management

In this session, we will talk about how some of the symptoms associated with MS increase the potential for falls. Because there are so many symptoms associated with MS, we will not be able to cover everything. Some symptoms that you experience may not be discussed today. However, just because we don't talk about them, it doesn't mean that they are not important! If you have questions about how additional symptoms can influence a fall, please speak with your trainer.

Many of the symptoms associated with MS can influence the occurrence of a fall.

Point of discussion: *What MS symptom(s) do you think contributed to a recent fall you experienced?*

While many symptoms contribute to a fall, some of the most common are:

- fatigue
- spasticity
- muscle weakness

The most important thing to remember is that you should **LISTEN TO YOUR BODY!**

You are an expert on how your body reacts to various medications, experiences, etc. Only you know how you feel at any given time of the day. Be aware of how your body feels, especially when you plan on participating in activities that are not a part of your daily routine. You may want to consider reorganizing your activities or ask for additional assistance during times you do not feel your best.

Below is a listing of how common MS symptoms can contribute to a fall. To read more about the influence of other symptoms, please look at the National Multiple Sclerosis Society's website:

https://www.nationalmssociety.org/Symptoms-Diagnosis/MS-Symptoms

**MS symptoms**	**How this symptom can contribute to a fall(s)**<br>**Source: National Multiple Sclerosis Society**
Fatigue	Fatigue is a very common MS related symptom. About 80% of individuals with MS experience fatigue. MS fatigue is commonly described as a feeling of exhaustion that is unrelated to an individual's level of exertion. This is different from muscle fatigue that results from physical activity.<br><br>Fatigue has been found to be a common factor associated with falls among wheelchair/scooter users. Wheelchair/scooter users with MS often reported that while performing a transfer or attempting to walk short distances they felt very tired and simply did not have the energy to complete the activity.<br><br>Therefore, if you are feeling fatigued, take a brief "time out" before engaging in an activity that you know you will need a lot of energy to perform. Think about if you have enough energy to do that activity and if there is another way that it could get done. For example: if you were planning on transferring to a tub bench to take a shower, consider taking a sponge bath in the short term and getting into the shower later when you have more energy. Alternatively, you may want to ask a care partner for additional assistance when doing energy-demanding activities.<br><br>The National Multiple Sclerosis Society has developed an extensive guide related to management of fatigue. It can be accessed at: https://www.nationalmssociety.org/NationalMSSociety/media/MSNationalFiles/Brochures/Brochure-Fatigue-What-You-Should-Know.pdf
Spasticity	Spasticity is one of the most common symptoms associated with MS and is a major contributor to falls. The term spasticity refers to feelings of stiffness and a reduced ability to maintain smooth, controlled movements of a limb. It may be as mild as a feeling of tight muscles, or may be so severe as to produce painful, uncontrollable spasms.<br><br>In falls related to spasticity, wheelchair/scooter users often reported that they were unable to control the movement in the affected extremity which resulted in loss of balance. If you frequently experience spasticity, be aware that this increases your risk of falling. If you need to carry out a strenuous task, such as performing a transfer or doing a complex

	wheelchair skill, try to think about other ways that you might want to do your desired activity.
Muscle Weakness	Muscle weakness is common in people with MS. Weakness in the muscles can cause shoulder, elbow, knee, hip, or ankle instability, which in turn can cause a fall while trying to stand, sit, or transfer. It is reported that wheelchair/scooter users with MS fell during daily activities, such as transfers, because their arms were weak.<br>More importantly, in previous studies, it was found that many wheelchair/scooter users with MS could not get up from a fall or move themselves to a communication device in order to seek help (e.g. pulling a cord, pushing an alarm button, or using a phone) due to muscle weakness.<br><br>The good news is that exercise can help you improve and maintain muscle strength. Further, research has found that exercise can help manage falls by improving muscle strength.

If any of these symptoms are causing significant problems for you, such as increasing the frequency of your falls, making it difficult for you to perform your activities of daily living, or limiting your engagement in desired activities, you might want to consider seeking help from a Physical or Occupational Therapist. To do so, call your doctor and make an appointment. Tell him/her that you have had a “decline in function” and what specific activities you can no longer perform. Your doctor will help you to decide if it would be best for you to see a Physical or Occupational Therapist.

Below is a listing of additional MS symptoms and how they can influence a fall. Please take a few additional minutes to look over these symptoms and how they might influence a fall. Your trainer will be happy to discuss these items further.

**Additional MS Symptoms**	**How this symptom can contribute to a fall(s)**<br>**Source: National Multiple Sclerosis Society**
Sensory deficits	Numbness and other sensory disturbances are often one of the first symptoms of MS. Numbness can make it difficult to be aware of the position of one's body parts in space. For example, it is reported that wheelchair/scooter users often fell when they reached forward to grab an object because numbness made it difficult for the wheelchair/scooter users to detect their body positions. As a result, they reached further than were able to and sustained a fall.<br><br>If you need to reach for an object but you don't feel confident in your balance, you may want to use an assistive device, such as a reacher, or ask a care partner to grab it for you.
Bladder and Bowel Dysfunction	Although they don't directly affect mobility, bladder and bowel dysfunction may cause an individual who has problems with urgency and/or frequency to rush to the bathroom.<br><br>As a result of bladder and bowel dysfunction, individuals with MS often reported that they fell while rushing to the bathroom because they were in a hurry and less attentive to fall hazards such as a slippery floor in the bathroom or clutter on the pathway to the bathroom.<br><br>If you frequently rush to the bathroom, especially at night, be aware that this increases your risk of falling. These are some tips that you can follow:<br><br>• Develop a habit of cleaning out the pathway from your bed to bathroom before you go to bed.<br>• Consider the use of a portable commode to keep beside your bed or in an area you are frequently in during the day.<br>• Turn on the light first when getting out of bed or install a motion activated light.<br>• Reduce your fluid intake (~1 hour) before bed.<br>• Consider the use of a protective pad (such as Depends)<br>• Ask your doctor about additional bladder management programs.

Cognitive Changes	The term cognition refers to a range of high-level brain functions. Cognitive changes are common in people with MS; approximately 50% may experience some difficulty with their cognition over the course of their disease. These problems may include difficulty focusing, maintaining and shifting attention, processing information quickly, and learning and remembering new information. Changes may also be seen in organization, planning and problem-solving skills, as well as the ability to accurately perceive one's environment.<br><br>In falls related to cognitive changes, wheelchair/scooter users with MS often reported that they were distracted during transfers, and that this resulted in falls. In addition, the chance of falling is increased when cognitive challenges interfere with a person's ability to focus on driving or propelling a wheelchair/scooter. For example, in previous studies it was found that participants often reported that they misjudged road conditions, and they were unable to detect obstacles on the street such as curbs, cracks or bumps.<br><br>It is important to focus on the activities that you are doing to reduce fall risks. For example, if your cell phone is ringing during a transfer, try not to answer the phone while transferring. Also, when you are driving your wheelchair/scooter on the street, especially in an unfamiliar environment, focus on the road conditions.

Session #4 Journal Entry

What will be done:

You will be asked to make a journal entry every week after completion of the session. Your journal entry will be tied to items discussed during the previous session. You will typically be asked to respond to a specific question regarding the content of the program from the previous session.

Why:

The journal entries will help you to think about the information that was presented during the education session and to help you integrate what you have learned into your everyday life.

Prompt:

Please think about one or two transfers that you performed last week. What type of transfer did you perform? Did you use any of the new techniques you learned? If so, did you feel any differences? If not, what prevented you for using the new techniques you learned?

Response:

<u>Manual Wheelchair Skills Education (Advanced)</u>

Get WISE Section: Wheelchair Management

Today, during the final wheelchair education session, we will learn and review some advanced wheelchair skills to allow you to safely and successfully navigate a variety of environments in your home and community.

<u>Going up/down a curb</u>

Level changes (e.g. curbs, steps, home entries, uneven sidewalk sections) are common obstacles found in everyday life and can be challenging to navigate. Appropriate technique to manage level changes is important to prevent a fall from occurring.

<u>Name of videos:</u>

- Going up a curb (MWC)
- Going down a curb in a wheelie (MWC)
- Going down a curb (MWC)

General tips to reduce the frequency of falls and injury:

When going up a curb

- ➢ Reposition or remove the rear anti-tippers, if necessary.
- ➢ Approach the curb at a slow but consistent speed. It is easier to pop the front wheels (casters) when moving.
- ➢ Don't lean forward when you approach the curb, it can increase the weight on the front wheels (casters) and make popping them up more difficult.
- ➢ Approach the curb with your front wheels (casters) pointing straight forward. Approaching the curb at an angle can make the skill more difficult.
- ➢ Pop the front wheels (casters) up from the ground, just high enough to clear the curb. It may take a few tries to get your timing down. Please see the instructions on how to "pop-up" the front wheels (casters) in Session #3 (Page 99).
- ➢ Lean forward slightly once your rear wheels have made contact with the curb. This will bring your front wheels (casters) back down.
- ➢ Continue to push and apply forces of moderate intensity to help your wheelchair climb up the curb.

When going down a curb

- Approach the curb while facing forward, as this allows you to watch for traffic while you complete the movement.
- When descending a curb, ideally you should perform the skill in a wheelie position to keep your body level and avoid falling forward. To perform the skill, put your chair in a wheelie position (as described below) and slowly roll forward, down the curb. You should **ONLY** attempt this skill if you feel confident in your ability to perform a wheelie on a level surface.
- If you are not comfortable performing a wheelie, push your wheelchair forward over the curb, shifting your weight slightly backwards to avoid falling forward.

Going up/down a slope

Pushing a wheelchair up ramps or hills are common obstacles found in everyday life. Although common, they can be difficult to navigate. Appropriate techniques are needed to avoid a fall.

Name of videos:
- Going up a ramp (MWC)
- Going down a ramp (MWC)

General tips to reduce the frequency of falls and injury:

When going up a slope (ramp)

- If you have a backpack or other gear on the back of your wheelchair, give it to a friend to carry (preferred), or if you are alone, put it on your lap. This will help prevent tipping backwards.
- Push forward onto the slope.
- Lean forward while going up the slope and use shorter, more frequent strokes. This will help keep your forward momentum going as you move up the incline.
- Place your feet on the footrest to avoid catching your foot on the ground when navigating the ground-slope transition at the start of the incline. Catching your foot on the ground could lead to a lower limb injury or a fall.
- If you get tired on the way up the incline, hold onto a handrail (if applicable) or ask for assistance.
- Consider using a “hill holders” device. This device allows the rear wheels to roll forwards, but not backwards.
- When you find a steep ramp that you can’t go up independently, ask for assistance.
    - Have your care partner walk behind you and ask him/her to push your wheelchair using the push handles or the back of your wheelchair.
    - Push forward on your hand rims at the same time.

When going down a slope

- Always check the slope for obstacles, such as cracks, before going down.
- Push your wheelchair forward onto the slope, shifting your weight slightly backwards to avoid falling forward.
- Proceed slowly to maintain control. Be prepared to stop at any time.
- Apply light but consistent pressure on the hand rims to control your speed. Gloves are helpful to avoid injuries.
- Place your feet on the footrest to avoid catching your foot on the ground when navigating the slope-ground transition at the bottom of the ramp.
- Use handrails if available.
- When you find a steep ramp that you can't go down independently, ask for assistance.
    - Have your care partner step behind you and hold onto the push handles.
    - Have your care partner roll the wheelchair down backward.
    - Apply pressure on the hand rims to help reduce your speed.
    - If possible, lean forward

<u>Traversing a side-slope</u>

Side-slopes or cross–slopes are common obstacles found in everyday life and can be challenging to navigate. For example, many sidewalks are sloped toward the street to allow water to run off. Side-slopes are also often found where sidewalks cross drive ways. Appropriate technique to manage side-slope is important to prevent a fall from occurring.

<u>Name of Video:</u>
Traversing a side-slope (MWC)

General tips to reduce the frequency of falls and injury:

- Push your wheelchair forward across the slope.
- Shift your weight slightly uphill-side to avoid falling sideways on steep cross-slopes.
- To avoid your wheelchair turning down hill, push harder on the downhill wheel.
- Different push frequencies may be required for each hand to keep the wheelchair moving straight. For example, when moving across a side-slope with the right side downhill, the right hand may push 2-3 times for every 1 push on the left.

<u>Performing a wheelie</u>

A wheelie is a method of lifting your front wheels (casters) off the ground. It is an essential skill for being able to independently navigate a variety of surfaces and challenging environments in a manual wheelchair. Performing a wheelie requires a lot of practice. If you are unable to master the skill during this education session, please consider asking your physician to order Occupational or Physical Therapy services.

Name of Video: Wheelie in place
Start: 0:18
End: 2:22

General tips to reduce the frequency of falls and injury:

You MUST have a friend or care partner spot you during practice.

Finding your balance point

- The key to doing a wheelie in place is finding your balance point.
- To find your balance point, please follow these steps
  - Lock your brakes and have a spotter tilt you and your wheelchair back to the point where you feel balanced between tipping forward and tipping backward. This may be farther back than you expect.
  - Look at how high your front wheels are off the ground to get a sense of your balance point.
  - Repeat this exercise with your brakes unlocked.
  - Once a spotter tilts back to the balance point with your brakes unlocked, gently rock forward and back around your balance point by pushing and pulling on your hand rims.
  - Try to relax your neck and shoulders and avoid controlling your balance by leaning with your head and shoulders.

After you've experienced your balance point

- Apply the wheelchair pop–up techniques discussed previously to tilt your wheelchair back into a wheelie position (see page 99).
- If you are having difficulty getting tipped far enough backwards to reach the balance point, push forward more forcefully.
- Try to maintain your balance in the wheelie position.
- To land, pull back on the wheels to gently bring the front wheels (casters) to the ground.

Care partner(s) spotting during wheelie practice

- Your care partner's (spotter) role is to prevent you from falling while performing this skill. Care partners and wheelchair users must communicate clearly before and during the practice.
- Make sure both you and your care partner are ready before starting a wheelie and talk through each step as you go.
- Have your care partner place a sturdy strap on the bar behind your wheelchair backrest and grasp firmly with one hand.
- If your wheelchair has anti-tippers that prevent wheelies, your care partner will need to remove them during wheelie practice.

- Your care partner should stand close in a slight lunge position behind the wheelchair, so that they can block the wheelchair from tipping back with their body as well as the strap.
- Your care partner should keep a hand free to grasp your shoulder if it looks like you might fall forward.

Power Wheelchair and Scooter Skills Education (Advanced)
Get WISE Section: Wheelchair Management

Today, during the final power wheelchair/scooter skill education session, we will learn and review some advanced wheelchair skills to allow you to safely and successfully navigate a variety of environments in your home and community.

Going up/down a curb

Level changes (e.g. curbs, steps, home entries, uneven sidewalk sections) are common obstacles found in everyday life and can be challenging to navigate. Appropriate technique to manage level changes is important to prevent a fall from occurring.

Name of video: Power Wheelchair Skills
Going up:
Start: 9:54
End: 10:08

Going down:
Start: 10:11
End: 10:17

General tips to reduce the frequency of falls and injury:

When going up a curb

- Search for an alternate route or a curb cut when you approach curbs.
- Elevate your footrest (if necessary) to increase ground clearance.
- Approach the curb at a slow speed and drive forward. Smooth, continuous forward movement is the most successful method of traversing an obstacle (As you see in the video, when the participant goes too slow, he cannot get up the curb).

When going down a curb

- Approach the curb while facing forward, as this allows you to watch for traffic while you complete the movement.
- Drive your wheelchair forward over the curb, shifting your weight slightly backwards to avoid falling forward.

Scooter Considerations

- Use the same techniques as described above.

- Scooters have smaller wheels compared to power wheelchairs and are not able to traverse high curbs. Very carefully consider the height of the curb before attempting to go/down. Have a care partner stand close to you when attempting a new curb.

<u>Going up/down a slope (ramp)</u>

Ramps or hills are common obstacles found in everyday life. Although common, they can be difficult to navigate. Appropriate techniques are needed to avoid a fall.

<u>Name of video:</u> Power Wheelchair skills
Going up a ramp:
Start: 8:30
End: 8:38

Going down a ramp:
Start: 8:40
End: 8:46

General tips to reduce the frequency of falls and injury:

Going up a slope (ramp)

- Use the tilt and/or leg-elevation functions to avoid scraping the footrests at the bottom of the ramp.
- Drive fast enough to maintain forward movement but not too fast that you lose control.
- Place your feet on the footrest to avoid catching your foot on the ground when navigating the ground-slope transition at the start of the ramp. Catching your foot on the ground could lead to a lower limb injury or a fall.

Going down a slope (ramp)

- Always check the slope for obstacles, such as cracks, before going down.
- Drive your wheelchair forward onto the slope, shifting your weight slightly backwards to avoid falling forward. If you have power tilt in space on your chair, put yourself in a little bit of tilt. This will help keep your body level when going down the ramp.
- Control your speed. Be prepared to stop at any time.
- Place your feet on the footrest to avoid catching your foot on the ground when navigating the slope-ground transition at the bottom of the ramp.

Scooter Considerations

- Scooters can tip over very easily, especially on ramps.
- You should only attempt minimal ramps in a scooter and have a care partner with you when attempting a new ramp.
- Do not make a sharp turn while going up/down a ramp.

Traversing a side-slope

Side-slopes or cross–slopes are common obstacles found in everyday life and can be challenging to navigate. For example, many sidewalks are usually sloped toward the street to allow water to run off. Side-slopes are also often found where sidewalks cross drive ways. Appropriate technique to manage side-slope is important to prevent a fall from occurring.

Name of Video: Power Wheelchair Skills
Start: 9:02
End: 9:25

General tips to reduce the frequency of falls and injury:

- Before traversing a slide-slope, check for wet or slippery surfaces (such as wet leaves) that will decrease traction. If these items are noted, take extreme caution and avoid the area if possible.
- Drive your chair at a moderate speed. You should use enough speed to keep you moving but not so much that you lose control.
- Drive slightly up-hill, if space allows.
- Shift your weight slightly uphill-side to avoid falling sideways on steep cross-slopes.

Scooter Considerations

- Use the same techniques as described above.
- Do your best to avoid steeper side-slopes. Scooters can tip over sideways very easily, especially on steeper side-slopes due to the relatively narrow width and high center of gravity.

Home and Community Participation

Get WISE Section: Individualized Activity in the Home and Community

Active participation in your home and community is important to the health and well-being of wheelchair and scooter users. This section with focus on helping you to think about activities you enjoy doing and how to develop the skills to do those activities.

Engaging in activities is important to your identity, to fulfilling roles and responsibilities, and to satisfaction with life. Research confirms that being actively involved in family and the community is vital to health and wellbeing[1] and has an important link to quality of life[2]. Being an active member of society is a key factor in sustaining health.[3] Considering your activity goals is the first step to ensuring ongoing participation in what matters to you.

Points of discussion:

- *What activities do you like to do for fun?*
- *Do you wish you could be involved in other activities?*
- *What type of activities would you like to be involved?*
- *What do you feel is holding you back from engaging in these valued activities?*

References

1. Liptak GS. Health and well being of adults with cerebral palsy. *Current Opinion in Neurology.* 2008;21:136-142.
2. Ravenek KE, Ravenek MJ, Hitzig SL, Wolfe DL. Assessing quality of life in relation to physical activity participation in persons with spinal cord injury: a systematic review. *Disability and health journal.* 2012;5(4):213-223.
3. Trieschmann RB. *Spinal Cord Injuries: Psychological, Social, and Vocational Rehabilitation.* 2nd ed. New York: Demos Publication; 1988.

Session #4 Goal

**Trainer Material:**
Please review the following materials with study participants and give them some time to work on their goal and action plans (~5-8 minutes).

During today's session, a discussion was held on safe participation in desired activities in your home and community. Goal setting is a way to help us focus and be strategic in life. Please write a goal related to how you will get started or progress in participating in an activity that is meaningful to you in the home or community. Please use the SMART goal format when writing your goal. This is a skill that takes practice; let's give it a try.

S	Specific.
M	Measurable.
A	Achievable.
R	Relevant.
T	Time bound.

Here is an example: *During the next week, I will ask my friend, Mary Smith, to go out to lunch at Applebee's, my favorite restaurant.*

Goal:

Think about some potential barriers to achieving your goal.
In Session 2, we introduced a strategy called action planning. I'm sure you remember, but as a refresher an action plan is a way to break down a large goal into small, manageable steps. This is a strategy or a tool to support you in achieving your goals.

Let's review again the key points to an action plan:

- Be sure your goal is manageable and you are confident of accomplishing it in a week.
- Your level of confidence should be a 7 or higher, or you should possibly reconsider the goal.
- The action plan helps you to break down a bigger task into manageable, thought out steps

ACTION PLANNING FORM

Think about the goal you have in mind related to how you will get started or progress participating in an activity that is meaningful to you in the home or community.

Step 1: What is your participation goal?

Step 2: What is one small step you could towards your goal this week?

What will you do?

When will you do it?

Step 3: Identify what will help you succeed:

Step 4: How confident are you that you can succeed in this step in the next 2 weeks?
1 2 3 4 5 6 7 8 9 10
(Not sure) (very sure)

*You should be a 7 or higher on this step. If you're not higher than a 7, let's take a closer look at the goal you have set.
==
Step 5: Assess your progress on step #1

Step 6: Identify specific, manageable next steps (then repeat Steps 2 thru 5 until complete).

Adapted from ©Self-Management Resource Center, LLC, 1980 - 2017. All rights reserved. All or portions of this material include copyrighted materials belonging to Self-Management Resource Center. To obtain a license please contact the Self-Management Resource Center. A Self-Management Resource Center Program (SMRC) developed by Dr. Kate Lorig, Virginia González and Diana Laurent.

Please take a moment to complete your action plan with the goal you have identified for participation.

Session #5 To Do's

Please complete the following items in the week between Session #4 and Session #5.

- Continue doing the home exercise program 3x/week (Page 16).
- Use your exercise log to track your exercises (folder)
- Goal form: Development of a plan for participation in desired activities (Page 120).
- Complete your journal entry reflecting on transfer skills (Page 110).

Wheelchair Set-Up

GET WISE Section: Wheelchair Management

The way that your wheelchair is set up can have a big influence on falling. Today, we will discuss some of the most common components that can influence the occurrence of a fall. When you are selecting a new wheelchair, please consider these various items. If possible, work with a therapist who is a certified Assistive Technology Professional (ATP). You can find an ATP in your region using the following website:

www.resna.org/member-directory/individual

Manual wheelchair rear axle position

On some manual wheelchairs, the axle associated with the large rear wheels can be moved back and forth. Getting this set up correctly can influence how easily your chair can tip over.

- When the axle is moved forward (towards the small wheels) the chair is less stable
- Moving the axle backwards, (further away from the small wheels) makes the chair more stable.

It is very important to strike a balanced position. It may be tempting to put the wheel in the most stable position to prevent a fall, however this can make the chair more difficult to push.

When the rear axle is moved further forward, the shoulder joint is in better alignment to push the chair, preventing the development of upper limb injuries. It also makes it easier to do several of the wheelchair skills that have been taught to you during this course.

Therefore, you want to try to move the rear axle as far forward as you feel comfortable, but you should also make sure that the chair is stable enough that it doesn't easily tip over backwards. As you become more proficient with your wheelchair skills, you can work to move the axle position further forward with the assistance of your wheelchair vendor.

In addition, adding heaving items on the back of your manual wheelchair, such as a heavy backpack, can make it easier for the chair to tip over backwards.

Manual wheelchair caster size

The size of the wheelchair front wheels(casters) can also have an influence on falls. Smaller casters allow for more mobility. They make it easier to do quick, small turns and are useful in tight locations. However, small casters can get caught in the sidewalks more easily. Larger casters don't get caught as easily but can be more difficult to maneuver.
Again, reaching a balance between functionality and stability is necessary! There are plenty of "mid-sized" options available!

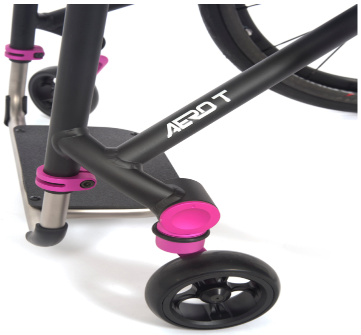

Figure 1: Mid-sized caster wheel*

<u>Wheel locks:</u>

Wheel locks or brakes are an important component of a manual wheelchair to increase the stability of the chair while doing activities. You can find a wide variety of wheel locks to fit your specific needs. For example, some wheel locks you push to engage while others pull to engage. Please talk with your therapist and/or wheelchair vendor to find the device that can work best for you.

For a wheel lock to provide stability, it must be engaged! Before performing a transfer or any other activities that requires your chair to be stable, take a moment to think if your wheel locks are engaged.

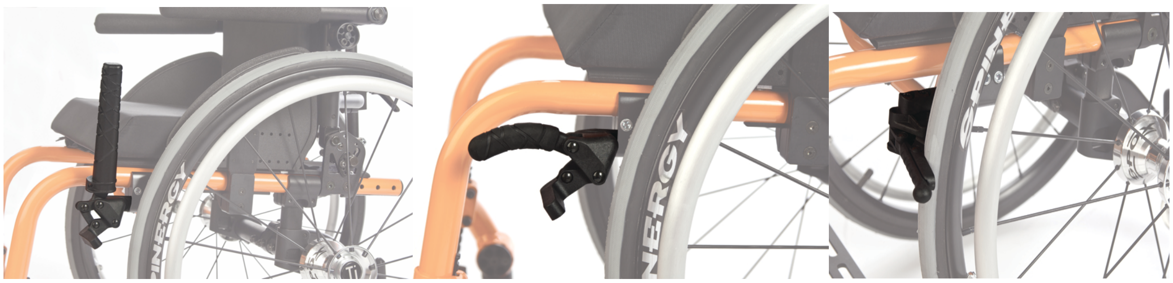

Figure 2: Various wheel locks (brakes)*

<u>Power wheelchair seat elevators:</u>

The use of a device that elevates the seat of a power wheelchair can be beneficial for prevention of falls. The seat elevator allows an individual to raise the height of the chair, which can make performing a transfer easier by allowing a user to transfer in a downward direction. A wheelchair user can also use the elevation feature to more easily reach for items above shoulder level. Some power wheelchairs also have the ability to tilt the seat of the chair in an anterior (forward) direction. This further helps a wheelchair user to safely reach necessary items.

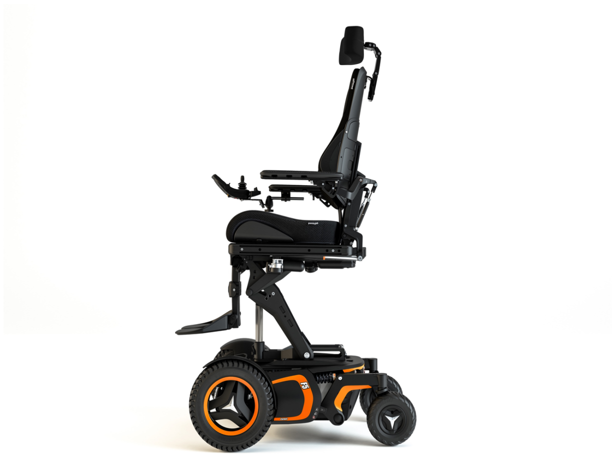

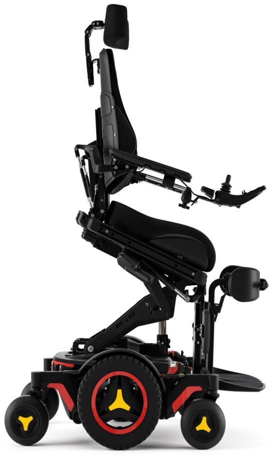

Figure 3: Seat Elevator*

Figure 4: Seat Elevator and Anterior Reach*

<u>A note about scooters:</u>

While a scooter can be very convenient and break down to fit into a car, the stability of the device is limited. When using a scooter be very careful when making sharp turns or reaching for items. Even small shifts in body weight can cause the device to fall over. If you are getting a new device, please consider the use of a power wheelchair. We recognize that there are a lot of changes that will need to be made, particularly associated with transportation. If you use a scooter, please talk with the trainer further about the benefits of a power wheelchair and how to make the transition as easy as possible.

After learning more about how wheelchair/scooter set up can influence a fall, take a few minutes to think about your wheelchair or scooter. Please answer the following questions below:

Has any aspect of your wheelchair/scooter set-up been associated with a fall?	**Circle one:**<br><br>Yes No	**Please describe the aspect of your wheelchair/scooter-set up that has been associated with a fall:**
What aspect of your wheelchair/scooter set-up to would you like to change to help prevent a fall?	**Please describe:**	
How will you go about making this change?	**Please describe:**	

* Photos courtesy of Permobil, AB

<u>Funding of Fall Prevention Equipment</u>

GET WISE Section: Environment

Getting the necessary equipment, such as wheelchair/scooters, reachers, tub benches, etc. to help prevent falls can often be challenging due to limitations in insurance coverage. Unfortunately, as a result of declines in budgets that fund both private and government-based insurance policies, the problem will likely get worse.

To have the best chance of getting insurance coverage for necessary equipment to prevent falls, it is essential that you work with a knowledgeable clinician who has experience writing letters of justification for equipment.

A good place to start is to work with a therapist who is a certified Assistive Technology Professional (ATP). You can find an ATP in your region using the following website:

www.resna.org/member-directory/individual

The National Muscle Sclerosis Society also has a database of healthcare professionals that are familiar with providing care to individuals with MS.

www.nationalmssociety.org/Treating-MS/Find-an-MS-Care-Provider

Despite the best efforts of therapists, there are often situations in which insurance companies simply do not provide funding for equipment that can be beneficial for the prevention of falls. For example, it is often very difficult to get funding for a seat elevator. Here is a listing of some resources that can help you to get the equipment that you need.

<u>National Multiple Sclerosis Society</u>: The National Multiple Sclerosis Society has some resources for equipment loan programs in which an individual can borrow a piece of durable medical equipment. You can contact the main number: 1-800-344-4867 or contact your local chapter directly. Information on local chapters (and other support resources) can be found here:

www.nationalmssociety.org/Resources-Support/Find-Support

<u>Centers for Independent Living</u>: The Administration for Community Living oversees a nationwide program to promote independent living for individuals with disabilities. These Centers for Independent Living (CIL) are nationwide and provide a variety of resources including durable medical equipment, care partners, etc. free of charge or at very reasonable rates. They can also help you to navigate insurance benefits. You can find a Center for Independent Living near you at:

www.acl.gov/programs/aging-and-disability-networks/centers-independent-living

www.ilru.org/projects/cil-net/cil-center-and-association-directory

Assistive Technology Loan Programs: The National Disability Institute supports an Assistive Technology Loan program in which loans are provided for Assistive Technology with very low or no interest. You can find out more about the programs at:

www.realeconomicimpact.org/asset-development/assistive-technology-loan-program

If you need a piece of equipment, don't give up! While the process can be long, there are several resources available to support living well with MS.

Please take a moment to think about a piece of equipment that might be useful to you and what resources you would use to get that equipment.

**What piece/pieces of equipment would be helpful to you to prevent falls?**	**Equipment:**
**What steps will you take to get this equipment?**	**Therapist:**<br><br>**Alternative Resources:**
**When will you start this process?**	**Specific date and time:**

Session #5 Journal Entry

What will be done:

You will be asked to make a journal entry every week after completion of the session. Your journal entry will be tied to items discussed during the previous session. You will typically be asked to respond to a specific question regarding the content of the program from the previous session.

Why:

The journal entries will help you to think about the information that was presented during the education session and help you integrate what you have learned into your everyday life.

Prompt:

Please think about your current wheelchair or scooter. How do you like it? Have you had any problems with the device? If yes, what kinds of problem did you have and how did you think you could solve the problems? Are there any new options you would like to get on your next wheelchair or scooter?

Response:

Wheelchair/Scooter Maintenance

Get WISE Section: Wheelchair Management

Properly maintaining a wheelchair/scooter is an important component of fall prevention. A recent study of power wheelchair/scooter users[1] found that wheelchair/scooter malfunctions were often associated with falls. Taking an active role in maintaining your chair or scooter will not only help prevent falls but it will also make pushing/driving your wheelchair or scooter easier. Below is a listing of items you should examine to assure your wheelchair is in good working order. To get more information about wheelchair maintenance, please visit the website: http://www.upmc-sci.pitt.edu/node/924.

Manual Wheelchairs:

- Tire pressure:
  - Name of video: Manual Wheelchair Maintenance
    - Start: 0:43
    - Stop 1:20
  - Check tire pressure on a weekly basis to assure tires are properly inflated. Note: Many power wheelchairs and scooters have solid tires that do not need to be inflated. Also, manual wheelchair casters (front wheels), often do not need to be inflated. Please talk with your wheelchair vendor or the trainer to determine what tires need to be inflated.
  - **How:** Press down on the tire with your thumb. If the tire presses down more than 5 mm, add additional air with an air compressor. The recommended tire pressure is often printed right on the rim of the tire. Using an air compressor, fill the tire to the specified level.
  - **Why?** Low tire pressure can:
    - make pushing a manual wheelchair more difficult.
    - make maneuvering a manual or power wheelchair or scooter more difficult.
    - make engaging the wheel locks of a manual wheelchair difficult and limit their ability to stabilize the chair when you transfer.

- Wheel locks
  - Name of video: Manual Wheelchair Maintenance
    - Start: 2:10
    - Stop 2:30
  - Wheel locks are used as "brakes" on a manual wheelchair to stop the wheels from rolling when you are in a stationary position. When performing a transfer or other functional activities, it is important that your wheelchair does not move.
  - **How:** Engage your wheel locks and try to spin your rear wheel. If more than a little movement is noted, you need to make an adjustment. First, check to make sure your tires are fully inflated (as described above). If they are, you will need to adjust the lock mechanism. Please talk with your wheelchair

vendor to learn how to adjust the wheel locks. Depending on the design, the adjustment may be complicated -- feel free to reach out to a care partner or the vendor to help you do the actual adjustments.
- **Why:** Wheel locks will provide a stable foundation for you to perform transfers from, reach for items and perform necessary activities in your home. If your chair is constantly sliding around, it will not only be difficult for you to perform these activities, but also puts you at a high risk of falling.

Manual Wheelchairs, Power Wheelchairs, and Scooters

- Casters (small wheels):
  - Name of video: Power Wheelchair Maintenance
    - Start: 1:40
    - Stop: 1:46
  - Check to make sure your small wheels (casters) are able to spin freely and don't "flutter" while your wheelchair/scooter is moving. Think about a shopping cart at the grocery store that is difficult to push. This is often due to problems with the small caster wheels. These problems will have a similar effect on your wheelchair/scooter. Hair, dirt and/or debris can easily get caught in these small wheels and affect their movement.
  - **How:** Have a care partner watch you push or drive your chair on a smooth, flat surface. The casters should roll smoothly and not "flutter," or quickly move side to side.
  - **Why:** A caster that does not spin freely or flutters will make maneuvering the wheelchair/scooter more difficult and decreases traction. In a worst-case scenario, a caster wheel can abruptly lock up and quickly stop your wheelchair/scooter. If you are not using your seat belt, you might fall out of the chair.

- Positioning belt (seatbelt)
  - Name of video: Power Wheelchair Maintenance
    - Start: 2:33
    - Stop: 2:46
  - A positioning belt, or seatbelt, will help keep you secure if your chair unexpectedly hits an obstacle.
  - **How:** Visually inspect the seatbelt to assure there are no rips in the fabric, cracks in the plastic or other damage. Also, check to make sure the buckle engages and stays secure when you pull on it. If you note a problem, call your wheelchair vendor immediately to get it fixed.
  - **Remember:** A positioning belt will only work if it is engaged! Don't forget to buckle up!

- Footplate attachment:
  - Check on a monthly basis that the bolts holding the footplate are secure and no excessive movement is noted when weight is put on the footplate.
  - **How:** Pull/push on various points of the footplate and watch for excessive motion.
  - **Why:** A loose footplate can:
    - Provide limited foot support and stability in your chair. This makes it more difficult to maintain your balance if you hit an unexpected object.
    - Scrape on the floor or ground which can make maneuvering the chair more difficult.
    - If the footplate abruptly falls down while you are moving, it can quickly stop the chair and potentially cause you to fall out.

In addition to the items noted above, check monthly for any loose nuts and bolts or cracks in the frame.

If you notice any problems with your wheelchair or scooter it is **VERY** important to notify the wheelchair vendor who sold you your wheelchair/scooter as soon as possible. He or she can perform repairs. At times, a loaner wheelchair or scooter can be provided to keep you going while your chair is being repaired. The vendor can also help you submit the cost of the repairs to your insurance company.

1. Rice LA, Sung J, Peters J, Bartlo WD, Sosnoff JJ. Perceptions of fall circumstances, injuries and recovery techniques among power wheelchair users: a qualitative study. *Clinical rehabilitation.* 2018:269215518768385.

Session #5 Goals

During today's session, a discussion was held on the importance of wheelchair and scooter maintenance.

It is therefore important that you know the contact information of your wheelchair or scooter vendor in case any repairs need to be done to the chair. Please take a few minutes to locate the name and phone number of your vendor. Often, the vendor will place a sticker directly on your chair/scooter with the company's phone number. If you need help, please ask the trainer to look over your chair/scooter to see if he/she can help you locate the phone number.

During the next week set a goal to find out the name and phone number (and be sure this information is accurate) of your vendor. Please write as much information as possible in the table below.

Name of Wheelchair/Scooter Vendor Company	
Name of Wheelchair/Scooter Vendor	
Phone number of Wheelchair/Scooter Vendor (if you have the vendor's cell phone number or direct office number, you may be able to speed up the process)	
E-mail address of the Wheelchair/Scooter Vendor	
Other important information	

If you cannot locate the information right now, please look for the information this week and complete the table. Think about a specific day and time that you will look for this information. Put the information in your calendar so you don't forget!

If you don't have regular contact with the vendor, please call the vendor to introduce yourself and make sure he/she is able to do repairs on your chair/scooter. If not, ask him/her to give you information of who can do the repairs.

If you received the wheelchair or scooter from a friend or family member or cannot find the information of the vendor, you can also contact the manufacture. Below are the customer service numbers of some of the major wheelchair/scooter manufactures. If you are having

trouble, please talk to the trainer. Please circle the number of the manufacture of your wheelchair or scooter.

Permobil/TiLite: 1-800-736-0925

Pride Mobility (Jazzy, Go-Go Scooters, Quantum Rehab): 1-800-800-4258

Invacare (TopEnd, Kuschall): 1-800-333-6900

Sunride Medical (Quckie, Sterling Scooters):

Hoveround: 1-800-96-HOVER

Other:

Name:

Phone number:

<u>Session #6 To Do's</u>

Please complete the following items in the week between Session #5 and Session #6.

- Continue doing the home exercise program 3x/week (Page 16).
- Use your exercise log to track your exercises (folder).
- Continue to work on your fall management plan (Page 85).
- Continue to work toward developing a plan to do a desired activity in your home or community (Page 120).
- Goal form: Identify the name of a person to contact if wheelchair/scooter maintenance is needed (Page 133).
- Complete your journal entry reflecting on your current wheelchair or scooter (Page 129).
- Think about how you will maintain your new skills in the long-term.

Sustaining Your Skills

Congratulations! You have worked your way through the i-ROLL program and have learned a lot of new information on ways to manage falls.

We have discussed:

- A variety of potential fall risk factors
- Consequences of falling
- Strategies to both prevent falls from occurring and manage a fall if it does occur

You also:

- examined your own risk factors of falls
- set up your own goals to manage a fall
- integrated skills into your daily routine.

Remember! This process does not stop at the end of the program. The process of managing fall risk is on-going.

For you to maintain the benefits of this program in the long term, it is necessary for you to work to sustain your newly learned skills and continue to engage in the exercise program.

You've already taken action to manage falls and fall risk since you have participated in the program. Here are a few tips to help you maintain your skills:

- **You are not alone!**
    - Talk to your peers, friends, family and care partners about the program and discuss the ways that they can help you maintain your skills. Your supporters can give you feedback on how you are performing your various skills and help to encourage you to use those skills to get out into the community and engage in your desired activities.
- **Be active!**
    - Your supporters can help you change, but they cannot change you. The most important thing is your willingness to change.
- **Set goals!**
    - Continue to set both short-term and long-term goals. These goals can help you get motivated to make changes or maintain a newly developed skill or habit.
- **Start it again!**
    - Often when people experience a life change, such as changing jobs, new romantic episode or break up, or moving to a new house, they may fall back into their old ways. If you find yourself getting off track and forgetting about

the skills you learned, do not worry. Pick up your program manual and look back over the items discussed.

- Think of the issues and problems.
- Discuss it with your peers, friends, family, and care partners.
- Start slowly re-integrating the items you learned and soon you will be back on track!

Maintaining Exercise Habits

Throughout the program we've discussed the benefits of exercise in management of MS related symptoms and managing fall risk. To review this information, please see the exercise program description in session 1, page 16.

As you work through this exercise program and others, please remember that you don't need to have full mobility to experience the health benefits of exercise. There are still plenty of ways you can engage in exercise to manage your symptoms, strengthen your muscles, and prevent falls

Here are tips to maintain the exercise habits.

- **Exercise with a friend(s).**
    - Ask your friends or family members to exercise with you. Your exercise partner can help keep you on track and motivate you to get moving.

- **Make it fun.**
    - Start with an activity you enjoy, go at your own pace. It's easier to stay motivated if you enjoy what you're doing, so find ways to make exercise fun. Talk to your local gym or community center (e.g. YMCA) about the fitness course that they offer which could be modified to meet your needs. Even if they don't offer any specific programs only for wheelchair/scooter users, many exercises/programs can be modified. Many fitness instructors will be willing to modify the exercise for you if you discuss your specific needs with him/her.

- **Try to Learn something new:**
    - Many sports and exercise are now accessible through adaptions or modifications. Include a variety of exercises in your routine and change it up from time to time. To read about the adaptive sports and exercise, please look at the National Multiple Sclerosis Society's website:

https://www.nationalmssociety.org/Living-Well-With-MS/Work-and-Home/Recreation/Finding-Another-Sport-to-Love

- **Set up realistic goals!**
    - Exercise is not magic and you can't expect immediate dramatic change. Listen to your body and do what you can. Start slow and gradually increase your activity level and keep your goals manageable. Success is accomplished by achieving a series of small goals. Accomplishing even the smallest fitness goals will help you gain confidence and keep you motivated.

- **Expect ups and downs.**

- Don’t be discouraged if you get off track. Pick up your program manual, review the exercise program and slowly build up your routine.

- **Reward yourself**
  - When you achieve your goals, reward yourself for achieving them. Think about something you like to do, write it down and go do it after you have achieved your goal. Also, a friendly competition with your exercise partner can help to motivate you to maintain your exercise routine.

- **Make exercise part of your daily life.**
  - Plan to exercise at the same time every day and stick with it. Life can be complicated and schedules vary. However, even if you can’t get your full work out in, try to do a few reps of all your exercises or do some type of alternative activity.
  - It takes time for a new activity to become a habit. To help keep you going, write down your reasons for exercising and a list of goals. Post them somewhere visible to keep you motivated.

Activity:
Please take a few minutes to think about your favorite skill you learned during the iROLL education session. Please write down 1-2 of your favorite new skills or something that has made a big impact on you.

Favorite Skills:

1. ____
2. ____
3. ____

After you have made your list of favorites, please think about how you will maintain these skills and integrate them into your daily life.

Methods to integrate:

1. ____
2. ____
3. ____

Session #6 Goal

During today's session, a discussion was held on maintaining the skills developed during the program in the long term. Please write a goal, using the SMART goal framework, to maintain the skills you learned during the iROLL program.

S	Specific.
M	Measurable.
A	Achievable.
R	Relevant.
T	Time bound.

Example: *I will review one section of my program manual each week for 15 minutes to keep the concepts discussed during this program fresh in my mind.*

Goal:

Session #6 Journal Entry
GET WISE Section: Goals

What will be done:

You will be asked to make a journal entry every week after completion of the session. Your journal entry will be tied to items discussed during the previous session. You will typically be asked to respond to a specific question regarding the content of the program from the previous session.

Why:

The journal entries will help you to think about the information that was presented during the education session and to help you integrate what you have learned into your everyday life.

Prompt:

Please write about your overall experience of the program. Please think about both positives and negatives of the program and what skills you want to continue to work on in the future (In addition to this journal entry, you will receive a follow up phone call in the next 48 hours to get your feedback on the program. Your training will give you additional details).

Response:
